# Multi-omics characterization of radiation-induced cerebellar remodeling and tumorigenic transcriptional programs

**DOI:** 10.1016/j.neo.2026.101333

**Published:** 2026-06-29

**Authors:** Fratini Emiliano, De Stefano Ilaria, Leonardi Simona, Pasquali Emanuela, Casciati Arianna, Tanno Barbara, Antonelli Francesca, Duchrow Lukas, Cemmi Alessia, Di Sarcina Ilaria, Mancuso Mariateresa, Kadhim Munira, Moertl Simone, Pazzaglia Simonetta

**Affiliations:** a Biotechnology Division, ENEA - Italian National Agency for New Technologies, Energy and Sustainable Economic Development, Rome, Italy; b Federal Office for Radiation Protection, Oberschleissheim, Germany; c Radiation Systems and Application Division, Nuclear Department, ENEA-Italian National Agency for New Technologies, Energy and Sustainable Economic Development, Rome, Italy; d Oxford Brookes University, Oxford, UK

**Keywords:** Cerebellum, Methylome, Proteome, Transcriptome, MB, Radiation doses, Patched1

## Abstract

Early-life exposure to ionizing radiation is a recognized risk factor for pediatric brain tumors, including medulloblastoma (MB), but the dose-dependent mechanisms linking radiation to tumor initiation remain poorly understood. Using the *Ptch1*⁺/⁻ mouse model of Sonic Hedgehog (SHH)-driven MB, mice were exposed at postnatal day 2 to 0.1 or 2 Gy γ-rays and monitored longitudinally, combined with multi-omics analyses to characterize early cerebellar responses and tumor-associated transcriptional programs. Radiation increased MB incidence in a dose-dependent manner, with 2 Gy significantly increasing tumor burden and reducing latency, whereas 0.1 Gy elevated tumor risk without affecting onset. Genome-wide methylation profiling revealed progressive dose-dependent epigenetic remodeling, with broader developmental and transcriptional regulatory changes after high-dose irradiation and limited signaling-associated alterations after low-dose exposure. Proteomic analyses identified common proliferative programs together with distinct dose-specific biological contexts, with low-dose irradiation preferentially associated with regulatory, transport, and selected immune-related pathways and high-dose exposure with chromatin organization, DNA replication, nucleotide metabolism, and RNA-associated processes. Radiation-induced MBs exhibited transcriptional architectures distinct from spontaneous tumors and differed according to radiation dose. Although involving largely distinct genes and pathways, independent methylomic, proteomic, and transcriptomic analyses converged on the identification of qualitatively distinct dose-associated biological programs. Notably, cross-omics functional concordance increased with radiation dose, revealing greater integration of molecular responses following high-dose irradiation. Together, these findings define dose-specific molecular states associated with radiation-induced MB and provide a biological framework linking early cerebellar remodeling to tumor heterogeneity, with implications for pediatric radiation risk assessment and therapeutic stratification.

## Highlights

• Radiation dose defines oncogenic trajectories in medulloblastoma.
• Early-life irradiation induces dose-specific molecular priming in the cerebellum.
• Low-dose exposure engages immune-modulated and microenvironmental pathways.
• High-dose irradiation drives replication stress and chromatin remodeling programs.
• Dose-imprinted molecular programs are retained in radiation-induced tumors.

## Introduction

Medulloblastoma (MB) is the most common malignant pediatric brain tumor and remains a major indication for craniospinal irradiation, despite efforts to reduce treatment-related toxicity. Molecular classification has identified distinct MB subgroups; among them, Sonic Hedgehog (SHH)–dependent MBs arise from granule cell progenitors (GCPs) of the developing cerebellum and rely on developmental signaling pathways [1,2]. Lineage-tracing studies have shown that transformation of GCPs during postnatal cerebellar development is sufficient to initiate SHH-driven MB [3,4]. Alterations in PTCH1, a key negative regulator of SHH signaling, define this subgroup and increase susceptibility to aberrant cerebellar growth and tumorigenesis [5].

Ionizing radiation is a well-established risk factor for pediatric brain tumors, including MB, as demonstrated by epidemiological studies of children exposed to therapeutic, diagnostic, or environmental radiation [6,7]. Importantly, the developing brain exhibits increased radiosensitivity, and radiation exposure during early life is associated with a disproportionately elevated risk of second malignant neoplasms compared with exposure in adulthood. This concern is particularly relevant in pediatric radiotherapy, where long-term survival has shifted clinical focus toward late effects, including radiation-induced secondary cancers [8,9].

Contemporary pediatric radiotherapy strategies increasingly emphasize dose reduction, highly conformal photon techniques, and proton therapy to minimize normal tissue exposure, particularly in very young children and genetically susceptible populations [[10], [11], [12]]. Despite these advances, long-term toxicity and second cancer risk remain major clinical challenges, underscoring the need for improved biological understanding of radiation effects in the developing brain [10,11].

While the biological consequences of high-dose radiation are relatively well characterized, the effects of low-dose radiation exposure, including diagnostic imaging, scattered dose, and out-of-field exposure, remain incompletely understood [7]. This knowledge gap is especially relevant for the cerebellum, where extensive postnatal proliferation and differentiation of GCPs coincide with windows of heightened radiosensitivity [13,14].

From a radiobiological perspective, high-dose radiation is classically associated with direct genotoxic effects, including DNA double-strand breaks and chromosomal instability, leading to cell-intrinsic oncogenic transformation [15]. In contrast, accumulating evidence indicates that low-dose radiation may engage qualitatively distinct mechanisms, including epigenetic modulation, altered intercellular signaling, immune and stromal interactions, and persistent changes in the tissue microenvironment, which may indirectly promote tumorigenesis [[16], [17], [18]]. Distinguishing between cell-intrinsic damage to the target cell population and non–cell-autonomous effects mediated by the microenvironment is therefore critical for accurate radiation risk modeling and for refining pediatric dose constraints [16].

Genetically engineered mouse models provide a powerful platform to investigate these questions in a controlled setting. Mice heterozygous for *Patched1* (*Ptch1*⁺/⁻) spontaneously develop MBs, a cerebellar tumor, that closely recapitulate the molecular, histopathological, and transcriptional features of human SHH-dependent MBs and show marked sensitivity to radiation-induced tumorigenesis [13,14]. This model is therefore particularly well suited to studying gene–radiation interactions, dose dependence, and the temporal evolution of radiation-induced molecular alterations in the developing cerebellum. In fact, compared to the rest of the brain, the cerebellum shows prolonged postnatal development, with neurogenesis continuing for up to two weeks in mice, and remarkable progenitor plasticity, enabling near-complete growth and functional recovery after acute granule cell depletion [19,20].

In this study, we combine tumour incidence analysis with integrated DNA methylome, proteomic and transcriptomic profiling to investigate the impact of low- and high-dose γ-irradiation during a critical window of susceptibility to MB in *Ptch1⁺/⁻* mice [13,14]. By profiling early cerebellar molecular alterations and comparing them with transcriptional programs of established MBs, we delineate dose-dependent biological programs across independent molecular layers, providing an integrated framework linking early cerebellar remodeling to radiation-induced MB development.

## Materials and methods

### Animals

Female CD1 mice were mated with males heterozygous for the *Ptch1*-null allele (CD1.129-Ptch1tm1Zim/Enea; hereafter referred to as *Ptch1^+/−^*). Offspring were irradiated at postnatal day 2 (P2) and genotyped as previously described [21]. Only *Ptch1^+/−^* mice were included in the present study. Detailed experimental procedures and bioinformatic analyses are described in the Supplementary Methods.

### Irradiation

At postnatal day 2 (P2), mice were exposed to γ-irradiation at the Calliope facility (ENEA Casaccia Research Center, Rome, Italy). Irradiation was performed at doses of 0.1 Gy or 2 Gy, delivered at dose rates of 7.33 mGy/min and 116 mGy/min, respectively. SHAM-irradiated controls underwent identical handling without radiation exposure. Absorbed dose was verified by Fricke dosimetry.

### Sample collection and tumor assessment

**Fig. 1:**
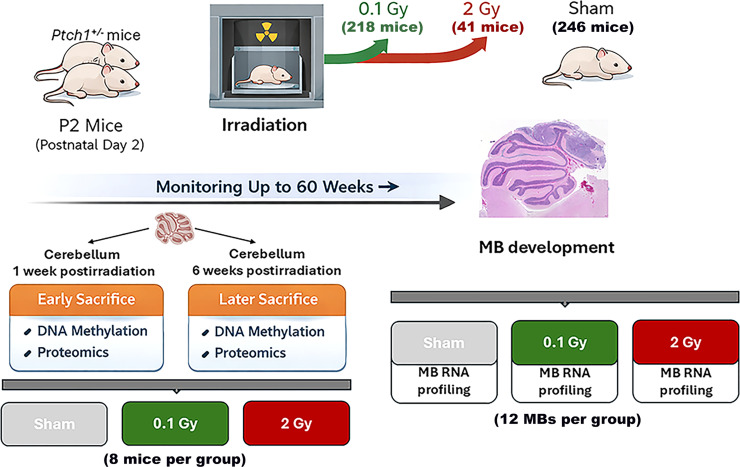
**Overview of the experimental workflow**. Schematic representation summarizing all key steps of the experimental design from sample size and preparation to final analysis.

Cerebella were collected at 1 and 6 weeks post-irradiation and snap-frozen for molecular analyses (n = 8 per group). A total of 505 mice (246 SHAM, 218 exposed to 0.1 Gy, and 41 exposed to 2 Gy) were monitored for up to 60 weeks for MB development. A schematic representation summarizing all key steps of the experimental design is shown on Fig. 1. Survival was defined as the average survival time, irrespective of the cause of death. Tumor latency was defined as the average time to the first appearance of MB. Tumor incidence was expressed as the percentage of mice with a histologically confirmed MB.

### DNA methylation analysis

Genomic DNA was extracted from frozen cerebellar tissue and analyzed by reduced representation bisulfite sequencing (RRBS; Active Motif). Reads were aligned to the reference genome using Bismark, and methylation levels at CpG sites were quantified. Differential methylation analysis was performed using the methylKit R package.

### Proteomic analysis

Proteins were extracted from frozen cerebellar tissue from four biological replicates per condition, each consisting of the cerebellum of one individual mouse, digested, and analyzed by DIA-PASEF mass spectrometry. Data were processed using Spectronaut against the SwissProt mouse database. Differential protein expressions were defined based on q-value < 0.05, at least two unique peptides, and fold change ≥1.5 or ≤0.6.

### Transcriptomic analysis

Total RNA was isolated from MB tissues and subjected to stranded total RNA sequencing. Libraries were sequenced on the SURFSeq 5000 platform. Sequencing data were processed using the nf-core/rnaseq pipeline. Reads were aligned with STAR and quantified with Salmon to generate gene-level count matrices.

### Differential expression and functional enrichment

Differential gene expression analysis was performed using DESeq2. Genes with adjusted p-value ≤ 0.05 and |log2FC| ≥ 1.5 were considered significantly differentially expressed. Functional enrichment analysis was conducted using g:Profiler, including Gene Ontology, KEGG, Reactome, and CORUM databases. Enrichment results were visualized and clustered using Cytoscape and the EnrichmentMap plugin.

### Network analysis

Gene interaction networks were generated in Cytoscape using RNA–RNA interactions from NPInter and protein–protein interactions from the STRING database.

### Quantitative real-time PCR (qPCR)

RNA from cerebella was reverse transcribed, and gene expression was quantified by real-time PCR using SYBR Green chemistry. Relative expression levels were calculated using the ΔΔCt method with *Gapdh* as reference gene.

## Statistical analysis

Statistical analyses were performed using GraphPad Prism software (GraphPad Software, San Diego, CA, USA). Kaplan–Meier survival analysis was used to estimate overall survival and tumor latency, and differences between groups were assessed using the log-rank (Mantel–Cox) test. A p-value < 0.05 was considered statistically significant. Statistical significance was indicated as follows: p ≤ 0.05 (*), p ≤ 0.01 (**), p ≤ 0.001 (***), and p ≤ 0.0001 (****).

## Results

### Early postnatal γ-irradiation induces dose-dependent MB development

**Fig. 2:**
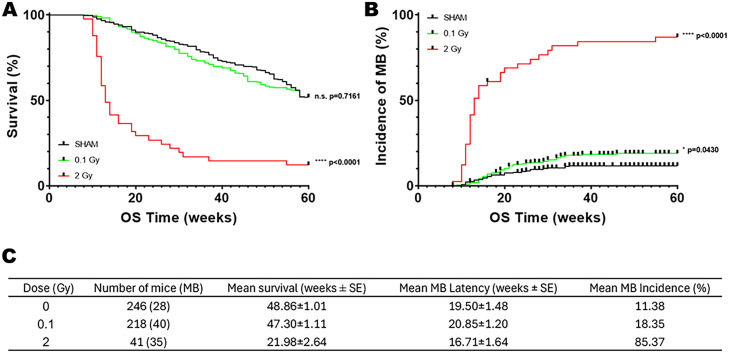
**Mouse survival and MB incidence**. (**A)** Life expectancy graphed as Kaplan-Meier percental survival for *Ptch1^+/−^* mice exposed to 0.1 Gy (green), 2 Gy (red) and SHAM (black); (**B)** MB incidence in *Ptch1^+/−^* mice exposed to 0.1 Gy (green), 2 Gy (red) and SHAM (black). Tick marks indicate censored animals. Significant differences were observed between groups (log-rank test, * p = 0.05; **** p < 0.0001).

To determine the impact of early-life γ-irradiation on MB development, *Ptch1⁺/⁻* mice were exposed at postnatal day 2 (P2) to 0.1 Gy or 2 Gy and followed longitudinally up to 60 weeks (Fig. 2A). Irradiation resulted in a clear dose-dependent increase in MB incidence compared with non-irradiated controls (Fig. 2B).

In the SHAM group, MBs developed in 28 of 246 mice (11.38% incidence) with a latency of 19.50 ± 1.48 weeks (Fig. 2C). Exposure to 0.1 Gy significantly increased tumor incidence (40/218 mice, 18.35%; *p* *=* *0.0430*) without substantially affecting latency (20.85 ± 1.20 weeks) or overall survival (47.30 ± 1.11 weeks vs 48.86 ± 1.01 weeks in SHAM). In contrast, 2 Gy exposure markedly increased tumor incidence (35/41 mice, 85.37%; *p* *<* *0.0001*) and significantly reduced latency (16.71 ± 1.64 weeks) and overall survival (21.98 ± 2.64 weeks).

These findings indicate that radiation dose influences not only tumor probability but also disease kinetics, providing a quantitative framework for subsequent molecular analyses.

### Dose-dependent DNA methylation remodeling in the cerebellum

To investigate early molecular events preceding tumor development, we performed genome-wide methylome profiling of cerebellar tissue at 1 and 6 weeks post-irradiation (Table S1).

**Fig. 3:**
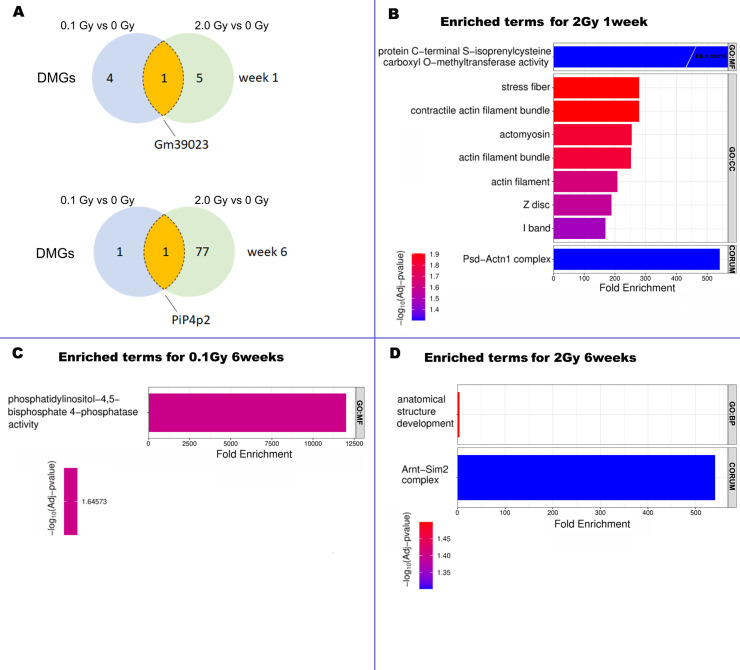
**Radiation-induced changes in the cerebellum methylome at 1 and 6 weeks after irradiation. (A)** Venn diagram showing number of DMGs identified in cerebella of 0.1 Gy and 2 Gy irradiated mice vs SHAM; Differential methylation was defined as a change in CpG methylation of >20% combined with a false discovery rate (FDR)-adjusted q-value < 0.05. Analyses were performed using four biological replicates (individually derived from four mice) per treatment condition. Functional enrichment analysis was performed using g:Profiler to identify enriched biological terms among Differentially Methylated genes (DMGs) at **(B)** 1 week after 2 Gy irradiation and, at 6 weeks after **(C)** 0.1 Gy and **(D)** 2 Gy, respectively. Statistically significant enrichment was determined at P-adj < 0.05. Enriched categories include Gene Ontology (GO) terms: Biological Process (BP), Cellular Component (CC), Molecular Function (MF); as well as protein complexes from CORUM. Bar colors represent statistical significance (red = lower P-adj, blue = higher P-adj). Fold enrichment values are shown on the x-axis; when a value exceeds the plotting range, it is displayed directly on the corresponding bar.

Differential methylation analysis revealed a clear dose- and time-dependent response. Pairwise comparisons between irradiated and SHAM groups (0.1 Gy vs SHAM and 2 Gy vs SHAM) were visualized using Venn diagram analysis to evaluate overlap among differentially methylated genes (DMGs). Analysis showed minimal overlap in DMGs between 0.1 Gy and 2 Gy conditions (Fig. 3A), indicating that cerebellar epigenetic responses are largely dose specific.

At 1 week post-irradiation, the number of DMGs was limited for both doses (5 at 0.1 Gy and 6 at 2 Gy). Nevertheless, DMGs identified after 2 Gy exposure were associated with pathways related to cellular organization and regulatory processes (Fig. 3B), whereas no significant enrichment was observed at 0.1 Gy.

By 6 weeks, differences between doses became more pronounced. High-dose irradiation induced a marked expansion of DMGs, accompanied by enrichment of developmental and transcriptional regulatory pathways (Fig. 3D). In contrast, low-dose exposure remained associated with very limited methylation changes and restricted pathway enrichment, primarily involving signaling-related processes (Fig. 3C).

Consistent with these findings, shared methylation events across doses were rare, with only one gene commonly affected at each time point. Notably, Pip4p2, a regulator of phosphoinositide signaling, was altered at 6 weeks, highlighting a potentially limited set of shared signaling nodes linking radiation exposure to neuroinflammatory regulation, in line with the established role of phosphoinositide pathways in microglial activation [22,23].

At the gene level, high-dose irradiation preferentially affected regulators of cerebellar development and neuronal differentiation, including *Zfp423* and *Cux2*, suggesting modulation of developmental programs involved in cerebellar progenitor differentiation [24,25]. In contrast, low-dose exposure mainly involved genes associated with intracellular signaling and cell–cell interactions, such as *Rap1gap*, indicating modulation of the neural microenvironment [[26], [27], [28]].

Together, these data indicate that cerebellar DNA methylation responses to irradiation follow distinct dose-dependent trajectories, with high-dose exposure inducing broader epigenetic alterations and low-dose exposure resulting in minimal and selective changes. Although involving relatively few differentially methylated genes, these findings indicate that radiation dose influences the biological nature rather than simply the extent of early epigenetic remodeling, with high-dose irradiation preferentially affecting developmental regulatory programs and low-dose exposure remaining restricted to signaling-associated processes.

### Dose-dependent proteomic responses in the irradiated cerebellum

Quantitative proteomic profiling revealed pronounced dose- and time-dependent alterations following early postnatal γ-irradiation (Table S2-S3).

**Fig. 4:**
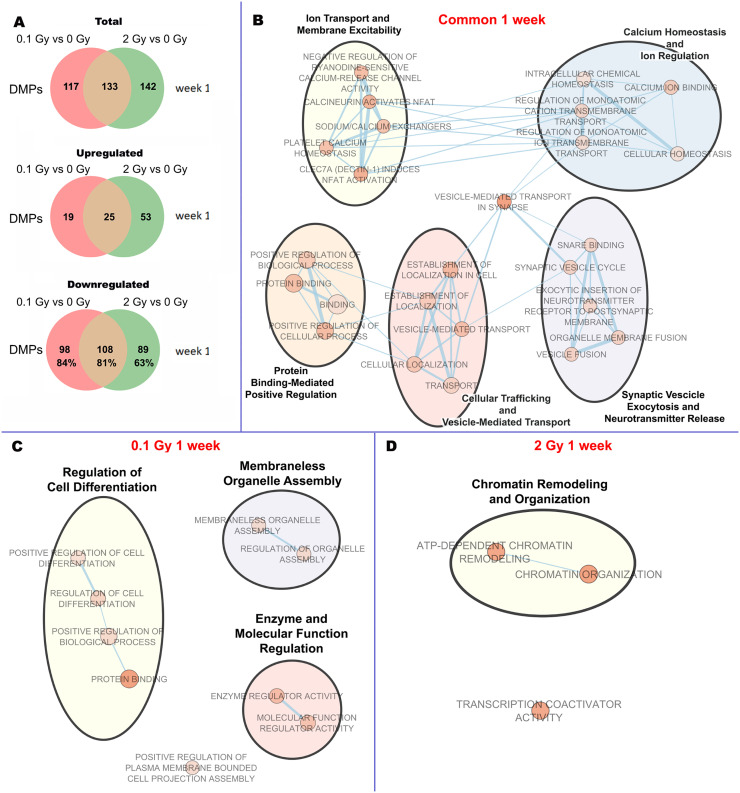
**Radiation-induced changes in the cerebellum proteome at 1 week after irradiation. (A)** Venn diagram showing number of modulated proteins identified in cerebella of 0.1 Gy and 2 Gy irradiated mice vs SHAM respectively (n = 4 cerebella for each dose); Differential protein expressions were defined based on q-value < 0.05, at least two unique peptides, and fold change ≥1.5 or ≤0.6. **(B-D)** Functional enrichment networks were generated via g:Profiler and EnrichmentMap to identify and cluster biological terms among proteins that were **(B)** commonly modulated between 0.1 Gy and 2 Gy or, **(C)** specifically modulated by 0.1 Gy, and **(D)** 2 Gy. Statistically significant enrichment was defined at Padj < 0.05. The plots in B-D display up to the top 5 deregulated pathways or less if a fewer significant deregulated pathways were identified; all the remaining statistically significant enriched pathways are shown in Table S4. The networks integrate data from Biological Process (BP), Molecular Function (MF), KEGG, and REACTOME (REACT) databases. In these maps, nodes represent enriched terms and edges represent gene overlaps between sets. Node color intensity reflects statistical significance of the gene set.

At 1 week post-irradiation, Venn analysis identified 133 commonly deregulated proteins (DEP), together with distinct dose-specific signatures (117 at 0.1 Gy and 142 at 2 Gy; Fig. 4A), indicating early divergence of molecular responses. Protein deregulation at this stage was predominantly characterized by downregulation across both doses.

Functional enrichment analysis showed that commonly deregulated proteins were primarily associated with neuronal signaling, ion transport, and vesicle-mediated processes (Fig. 4B; Fig. S1A), consistent with an early perturbation of cerebellar physiology. Beyond this shared component, dose-specific programs clearly emerged. Low-dose irradiation (0.1 Gy) preferentially affected pathways related to differentiation, molecular regulation, and cellular organization (Fig. 4C), consistent with selective modulation of differentiation and regulatory pathways. In contrast, high-dose irradiation (2 Gy) selectively disrupted chromatin organization and transcriptional regulation pathways (Fig. 4D, Table S4), consistent with nuclear and chromatin-associated remodeling. The complete pathway enrichment analysis reported in Table S4 further indicates that these biological programs extend beyond the top-ranked terms displayed in the network representation, reinforcing the segregation between low- and high-dose responses. STRING network analysis further highlighted dose-dependent differences in proteomic organization. Low-dose exposure was characterized by multiple small and distributed interaction clusters, whereas high-dose irradiation showed increased connectivity within STRING interaction networks (Fig. S1B–C).

**Fig. 5:**
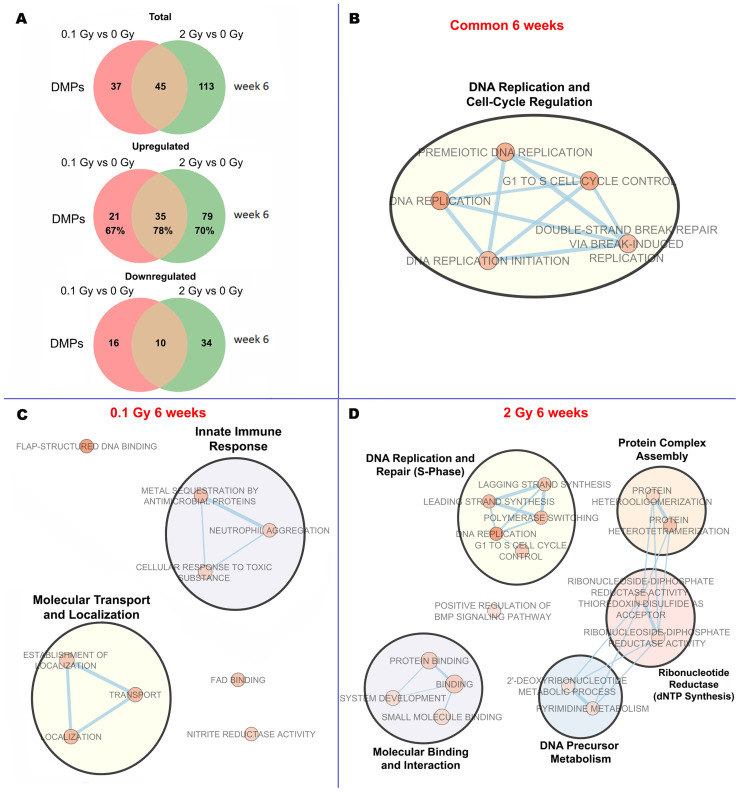
**Radiation-induced changes in the cerebellum proteome at 6 weeks after irradiation. (A)** Venn diagram showing number of modulated proteins identified in cerebella of 0.1 Gy and 2 Gy irradiated mice vs SHAM respectively (n = 4 cerebella for each dose); Differential protein expressions were defined based on q-value < 0.05, at least two unique peptides, and fold change ≥1.5 or ≤0.6. **(B-D)** Functional enrichment networks were generated via g:Profiler and EnrichmentMap to identify and cluster biological terms among proteins that were **(B)** commonly modulated between 0.1 Gy and 2 Gy or**, (C)** specifically modulated by 0.1 Gy, and **(D)** 2 Gy. Statistically significant enrichment was defined at Padj > 0.05. The plots in B-D display up to the top 5 deregulated pathways or less if a fewer significant deregulated pathways were identified; all the remaining statistically significant enriched pathways are shown in Table S5. The networks integrate data from Biological Process (BP), Molecular Function (MF), KEGG, and REACTOME (REACT) databases. In these maps, nodes represent enriched terms and edges represent gene overlaps between sets. Node color intensity reflects statistical significance of the gene set.

By 6 weeks post-irradiation, proteomic responses remained strongly dose dependent, although a larger overlap in deregulated proteins emerged between irradiation conditions compared with the early time point (Fig. 5A). Shared alterations were primarily associated with DNA replication and cell-cycle–related pathways (Fig. 5B), consistent with persistent engagement of proliferative and cell-cycle–associated programs in the irradiated cerebellum. As shown in Table S5, the complete enrichment profile confirms that common proliferative programs coexist with dose-specific functional contexts involving transport/localization pathways and immune response pathways after low-dose irradiation and replication- and RNA-associated pathways after high-dose exposure. STRING network analysis of commonly deregulated proteins revealed interconnected clusters associated with nuclear organization, DNA replication, and cell-cycle regulation (Fig. S2A). Despite this partial convergence, distinct biological programs remained evident between doses. Low-dose exposure was associated with enrichment of immune-related, transport, and localization pathways (Fig. 5C, Table S5), while the corresponding STRING network showed a more distributed organization with smaller interaction clusters (Fig. S2B). In contrast, high-dose irradiation, maintained enrichment of pathways linked to DNA replication, nucleotide metabolism, and RNA-associated functions (Fig. 5D, Table S5), with STRING analysis revealing larger and more interconnected protein interaction networks (Fig. S2C).

Overall, proteomic analyses indicate that low- and high-dose irradiation differ not simply in the magnitude of protein deregulation but in the biological programs engaged. Although 0.1 Gy and 2.0 Gy share common pathways, the surrounding functional context remained dose specific with additional unique signatures, supporting the existence of qualitatively distinct molecular states linked to radiation dose.

### Dose-specific transcriptional programs in the cerebellum and radiation-induced MBs

To investigate whether early irradiation-induced transcriptional alterations preceded the emergence of dose-specific tumor programs, we first analyzed gene expression changes in the cerebellum 1 week after irradiation. Expression analysis showed no major changes in cerebellar lineage markers or inflammatory mediators following irradiation (Fig. S3). These findings indicate that early irradiation-induced cerebellar alterations occur in the absence of widespread transcriptional reprogramming detectable at the whole-tissue level.

**Fig. 6:**
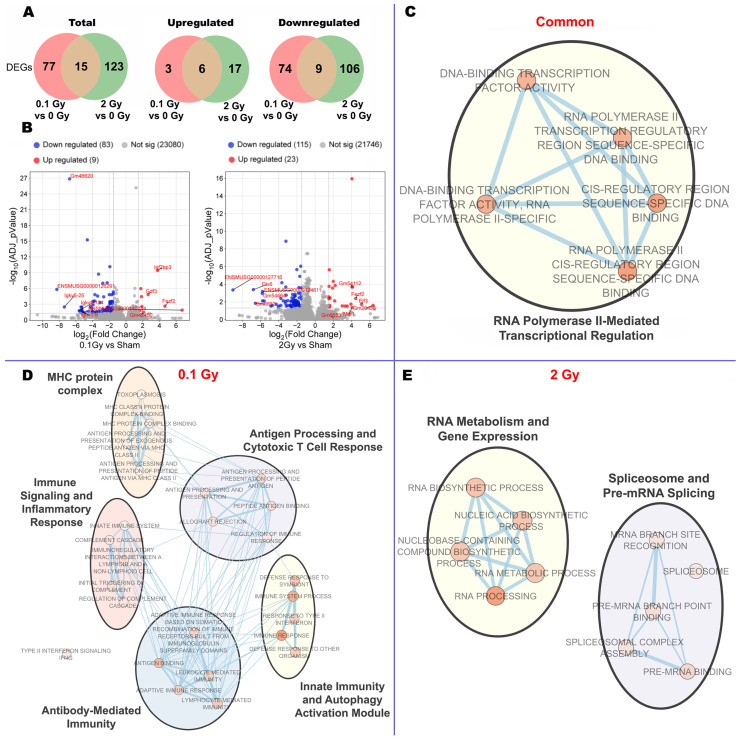
**Transcriptomic analyses of 0.1Gy- and 2Gy-induced MBs. (A)** Venn diagram showing number of DEGs identified in MB of 0.1 Gy (n = 12) and 2 Gy (n = 12) irradiated mice vs SHAM (n = 12); Four pooled samples were generated for each experimental condition, each comprising three MBs; **(B)** Volcano plots of DEGs between 0.1 Gy (left) and 2 Gy (right) irradiated vs. SHAM. Each plot shows the changes in MB gene expression in the respective irradiated compared to SHAM. The red dots indicate the upregulated DEGs with log_2_(FC) > 1.5, whereas the blue dots indicate the downregulated DEGs with log_2_(FC) < −1.5. Padj < 0.05; **(C-E)** Functional enrichment networks generated via g:Profiler and EnrichmentMap to identify and cluster biological terms among genes **(C)** commonly modulated between the two doses, or, **(D)** specifically modulated in MB induced in 0.1 Gy irradiated mice, or **(E)** 2 Gy irradiated mice. Statistically significant enrichment was defined at Padj < 0.05; the plots in C-E display up to the top 5 deregulated pathways or less if a fewer significant deregulated pathways were identified; all the remaining statistically significant enriched pathways are shown in Table S6. The networks integrate data from Biological Process (BP), Molecular Function (MF), KEGG, and REACTOME (REACT) databases. In these maps, nodes represent enriched terms and edges represent gene overlaps between sets. Node color intensity reflects statistical significance of the gene set.

Despite the limited transcriptional alterations detectable in the early irradiated cerebellum, we next investigated whether distinct dose-dependent molecular programs emerged during tumor development by performing transcriptomic profiling of spontaneous and radiation-induced MBs. Differential expression analysis identified distinct dose-specific transcriptional programs (Padj < 0.05; |log2FC| ≥ 1.5; Table S4). A total of 77 DEGs were detected in 0.1 Gy–induced tumors and 123 in 2 Gy–induced tumors, with only a small subset shared between conditions (Fig. 6A), indicating largely divergent transcriptional architectures. In both groups, gene repression predominated, particularly in high-dose tumors.

Volcano plot analysis further highlighted the broader and more pronounced transcriptional remodeling induced by 2 Gy irradiation (Fig. 6B), consistent with stronger perturbation of tumor regulatory programs. Despite these differences, a small set of genes commonly deregulated in radiation-induced tumors defined a shared regulatory interaction network identified in radiation-induced MBs (Fig. 6C; Fig. S4A; Table S6), indicating that radiation-induced tumors share a limited set of regulatory features despite otherwise distinct dose-associated transcriptional architectures.

At the pathway level, dose-specific programs clearly emerged. Low-dose tumors were enriched for immune-related and inflammatory pathways (Fig. 6D; Table S6). Network analysis of 0.1 Gy–specific DEGs revealed a limited interaction structure, composed mainly of mRNA nodes with few coding–noncoding connections (Fig. S4B), complementing the immune-related enrichment observed at the pathway level. However, key components of antigen presentation and interferon-associated signaling were broadly downregulated (Tables S7 and S8), suggesting suppression of immune-related signaling pathways.

In contrast, high-dose tumors were dominated by RNA metabolism and gene expression pathways, including spliceosome activity and transcriptome remodeling (Fig. 6E, Table S6). Network analysis revealed extensive interaction networks involving coding and noncoding RNAs in 2 Gy–induced tumors (Fig. S4C), indicating broad disruption of RNA-associated regulatory programs.

Overall, radiation-induced MBs arising after low- and high-dose exposure displayed distinct transcriptional architectures. Together with the dose-dependent epigenetic and proteomic remodeling observed in the developing cerebellum, these findings indicate that independent molecular layers consistently discriminate low- and high-dose irradiation through distinct biological programs, despite limited overlap at the individual gene level.

### Convergent dose-dependent molecular programs across omics layers

Viewed together, the independent methylomic, proteomic, and transcriptomic analyses consistently discriminated low- and high-dose irradiation through distinct biological programs. Although involving largely non-overlapping genes and enriched pathways, all three molecular layers independently supported the same biological conclusion, indicating that radiation dose is associated with qualitatively distinct molecular states rather than different magnitudes of a common response. The complete pathway enrichment analyses (Tables S4–S6) further demonstrate that this dose-dependent divergence extends across the broader functional landscape identified by each omics platform.

Some overlap was observed between early cerebellar proteomic alterations and transcriptomic changes in radiation-induced MBs at the individual-gene level. *Crb2* and *Col1a1* were the only shared differentially regulated genes. *Crb2* showed no consistent directional change, whereas *Col1a1*, which encodes a major extracellular matrix protein involved in tissue remodeling and cell–matrix interactions and is associated with extracellular matrix programs in SHH MB [29], was consistently downregulated in both datasets. This suggests that extracellular matrix remodeling may be one of the few biological processes linking early cerebellar responses to the molecular organization of radiation-induced MBs.

**Fig. 7:**
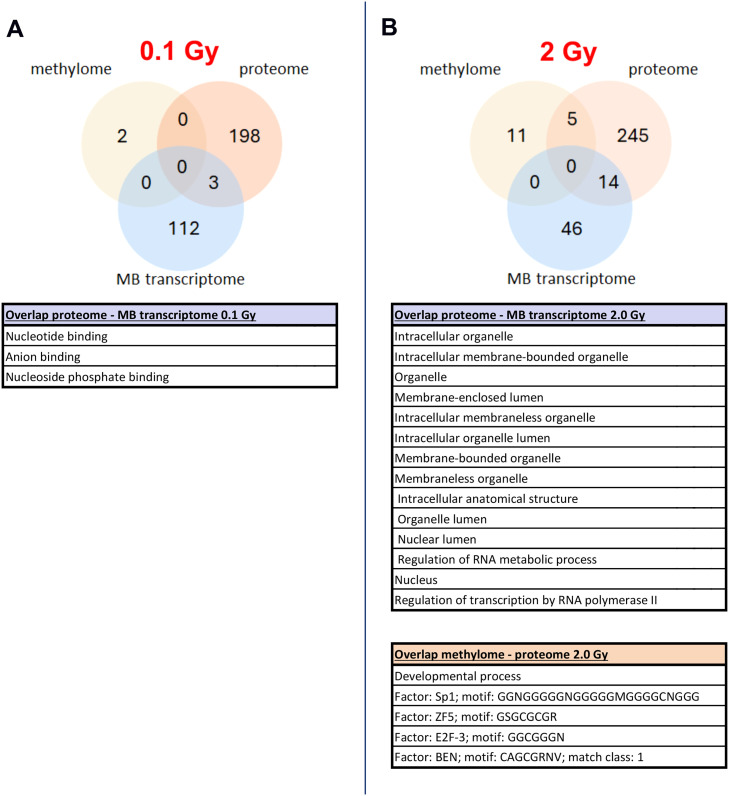
**Pathways overlap between omics datasets**. Venn diagrams illustrate the overlap of significantly enriched functional categories identified from radiation-induced alterations in the DNA methylome and proteome of normal cerebellar tissue and radiation-associated transcriptomic changes in MB. (A) shows the overlap following exposure to 0.1 Gy, whereas (B) depicts the overlap after exposure to 2.0 Gy. The overlap includes all statistically significant functional enrichment terms obtained by g:Profiler using the complete sets of significantly radiation-altered methylation sites, differentially expressed proteins, and radiation-associated transcripts identified at the respective dose. Enrichment categories comprised Gene Ontology (GO) Biological Process (BP), Molecular Function (MF), and Cellular Component (CC) terms, KEGG pathways, Reactome pathways, transcription factor (TF) targets, Wiki pathways (WP) and CORUM protein complexes. The tables associated with the Venn diagrams report the shared pathways.

At the pathway level, overlap across molecular layers was strongly dose dependent. Following 0.1 Gy irradiation, shared pathways were detected exclusively between cerebellar proteomic alterations and MB transcriptomic changes, with only three common pathways identified, of which only one was also shared with the corresponding 2 Gy dataset. In contrast, 2 Gy irradiation showed substantially greater concordance across omics layers, with five shared pathways between the methylome and proteome and fourteen shared pathways between the proteome and MB transcriptome. These common biological programs were predominantly associated with intracellular and nuclear organization, including intracellular and membrane-bound organelles, organelle lumen, membraneless organelles, nuclear lumen, and intracellular anatomical structures. Additional concordant pathways involved regulation of RNA metabolic processes, RNA polymerase II-dependent transcription, and other nuclear-associated functions. Overall, these findings indicate that cross-omics convergence increases with radiation dose and emerges primarily at the level of biological organization rather than shared molecular components. This integrative framework is summarized in Fig. 7.

## Discussion

In this study, we investigated how radiation-dose influences tumor development in *Ptch1⁺*^/^*⁻* mice, a model predisposed to SHH-driven MB. γ-irradiation during a critical neonatal window increased tumor risk in a dose-dependent manner. High-dose exposure (2 Gy) markedly increased tumor incidence and shortened latency, whereas low-dose exposure (0.1 Gy) significantly elevated tumor risk without accelerating tumor onset. Multi-omics analyses revealed dose-dependent epigenomic and proteomic alterations in the normal cerebellum, together with distinct transcriptional programs in radiation-induced MBs.

The principal finding of this study is that radiation dose influences not only MB risk but also the biological programs through which tumor development proceeds. Methylomic, proteomic, and transcriptomic analyses consistently identified dose-specific biological programs across independent molecular layers that emerge during cerebellar development and remain reflected in radiation-induced MBs.

**Fig. 8:**
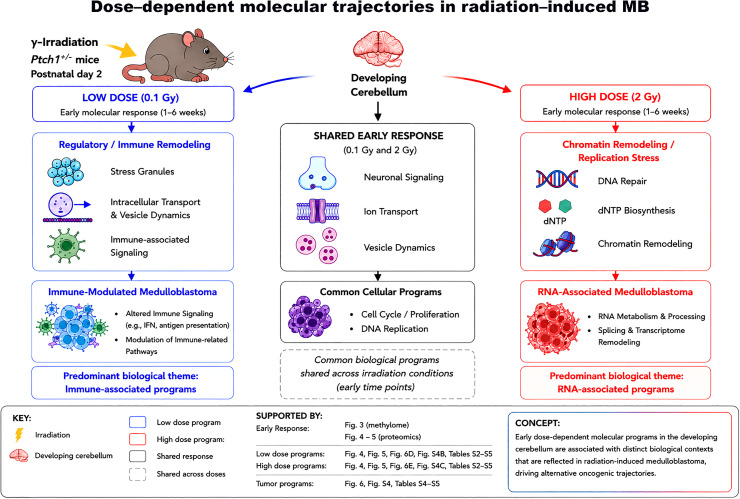
**Conceptual model summarizing dose-associated molecular trajectories in radiation-induced MB**. Schematic representation of the dose-dependent biological programs identified following early-life γ-irradiation in *Ptch1^+/−^* mice. Irradiation at postnatal day 2 induces distinct molecular responses in the developing cerebellum and MBs that diverge according to radiation dose. Low-dose exposure (0.1 Gy) was more closely associated with regulatory and selected immune-related programs, whereas high-dose irradiation (2 Gy) preferentially engaged chromatin remodeling, replication stress, and RNA-associated pathways. Although both exposure conditions ultimately led to MB formation, the molecular contexts associated with tumor development were distinct between doses.

Current models of radiation carcinogenesis primarily attribute tumor initiation to DNA damage and error-prone repair. While these mechanisms undoubtedly represent the initiating event, our findings suggest that radiation dose also determines the developmental and molecular environment in which transformed cells evolve. This observation suggests that radiation dose contribute not only to tumor initiation but also to the establishment of different biological contexts that may influence subsequent tumor evolution, providing the conceptual basis for the integrated model proposed in Fig. 8.

The model illustrates that dose-dependent molecular divergence is progressively established during postnatal cerebellar development, becoming more pronounced between 1 and 6 weeks after irradiation and remaining reflected in the molecular architecture of radiation-induced MBs. Following 0.1 Gy exposure, cerebellar remodeling is characterized by relatively selective molecular changes involving regulatory and selected immune-associated pathways, stress granule formation, and modulation of intracellular transport and vesicle dynamics. In contrast, 2 Gy irradiation induced broader molecular alterations dominated by chromatin remodeling, DNA repair activation, nucleotide biosynthesis, and replication stress–associated programs consistent with a more extensive genome maintenance response. Despite these distinct early trajectories, both irradiation conditions ultimately converge on proliferative programs involving DNA replication and cell-cycle progression, indicating that sustained proliferative pressure represents a common feature of radiation-induced tumorigenesis. This convergence occurs within different biological contexts: low-dose exposure retains regulatory and selected immune-related programs, whereas high-dose irradiation remains dominated by replication stress, DNA repair, and RNA metabolism. Radiation-induced MBs arising after low-dose exposure exhibit transcriptional programs enriched for regulatory and immune-associated processes, whereas tumors induced by high-dose irradiation display extensive coding and noncoding RNA interaction networks together with pathways related to RNA metabolism and transcriptome remodeling. Together, these observations suggest that early dose-dependent molecular perturbations are progressively reinforced during postnatal cerebellar maturation and remain reflected in tumor transcriptomes, although direct mechanistic relationships cannot be established from the present data. Thus, radiation dose appears to shape the biological context of tumor evolution rather than simply modulating the intensity of the initial radiation response.

High-dose irradiation induced early chromatin- and nucleus-centered alterations associated with transcriptional regulation and DNA damage responses that were already detectable at 1 week and became substantially more pronounced by 6 weeks, consistent with sustained epigenetic remodeling. In contrast, low-dose exposure produced markedly fewer methylation changes throughout the observation period, supporting the existence of qualitatively distinct epigenetic responses rather than different magnitudes of a common radiation effect. The shared deregulation of *Pip4p2* suggests the persistence of common signaling nodes potentially linking radiation exposure to neuroinflammatory regulation [22,23].

Representative gene-level alterations identified by the methylomic analysis further exemplify these distinct biological programs. High-dose irradiation preferentially affected developmental regulators such as *Zfp423* and *Cux2*, transcription factors involved in cerebellar progenitor identity and neuronal differentiation [24,25], consistent with the broader engagement of developmental regulatory networks after 2 Gy exposure. Conversely, low-dose irradiation was associated with methylation changes affecting signaling-associated genes, exemplified by *Rap1gap*, a key regulator of RAP1-dependent cell adhesion and intracellular signaling [[26], [27], [28]].

Importantly, this early dose-dependent molecular divergence was also reflected in the transcriptomic architecture of radiation-induced MBs. Tumors arising after low-dose exposure preferentially exhibited regulatory and selected immune-related programs, despite the downregulation of several genes involved in antigen presentation and interferon signaling, including *Tap1, Cd74, H2-Aa, Cxcl9, and Nlrc5*. In contrast, high-dose tumors showed stronger enrichment of RNA metabolism, spliceosome-associated functions, and transcriptome remodeling pathways, accompanied by distinct coding and noncoding RNA interaction networks. Together, these findings indicate that the molecular divergence established during cerebellar development remains preserved in radiation-induced tumors.

Interestingly, a major insight from the cross-omics analysis is that the functional concordance across molecular layers increased with radiation dose. The stronger convergence observed after 2 Gy exposure does not reflect a larger number of deregulated pathways, which was broadly comparable between doses, but rather a higher degree of functional alignment across molecular layers. At 0.1 Gy, pathway deregulation was detectable but showed limited cross-omics overlap, mainly involving broad binding-related categories and suggesting a diffuse, poorly integrated response. In contrast, 2 Gy exposure produced a coherent multilayered signature, linking methylomic, proteomic, and transcriptomic alterations to intracellular and nuclear organization, RNA metabolism, RNA polymerase II-dependent transcription, and developmental/proliferative regulatory programs, including Sp1- and E2F-3-associated motifs. This interpretation is reinforced by STRING analyses, which showed more interconnected functional modules after high-dose irradiation and smaller, more dispersed clusters after low-dose exposure. Thus, radiation dose does not simply determine which biological processes are affected, but also how strongly these processes become integrated across omics layers, with 2 Gy aligning early cerebellar remodeling with tumor-associated transcriptional programs and 0.1 Gy producing a more fragmented and context-specific response.

These findings are particularly relevant in the context of cerebellar development, which is characterized by prolonged postnatal maturation and marked progenitor plasticity. During the early postnatal period, GCPs undergo rapid proliferation under SHH signaling, creating a developmental window of heightened vulnerability to oncogenic perturbations. Radiation exposure during this stage may therefore disrupt regulatory programs controlling proliferation and differentiation, increasing the likelihood that susceptible progenitor populations acquire tumorigenic potential [19,20]. Within this framework, *Ptch1⁺^/^⁻* mice provide a biologically relevant experimental system. Haploinsufficiency of *Ptch1* results in constitutive SHH pathway activation in GCPs, the cells of origin of SHH-driven MB. Irradiation during the neonatal proliferative phase may therefore alter the balance between progenitor expansion and differentiation, either increasing the pool of susceptible cells or modifying developmental programs associated with tumor susceptibility [13,14,30,31].

The widespread use of ionizing radiation in medicine, together with occupational and environmental exposure, has intensified concerns regarding the biological consequences of low-dose irradiation. Although epidemiological findings remain heterogeneous, increasing evidence suggests that even low radiation doses can induce measurable biological alterations across multiple tissues. According to UNSCEAR, low-dose exposure is generally defined as ≤100 mSv, with organ doses from X/γ radiation typically below 100 mGy [32]. Importantly, our study analyzed an unusually large cohort of mice, including 218 animals exposed to 0.1 Gy, providing sufficient statistical power to detect biologically meaningful effects at low dose. These findings support the concept that early-life low-dose irradiation can influence tumor susceptibility in genetically predisposed individuals.

These observations are relevant to the ongoing debate surrounding the linear no-threshold (LNT) model at low radiation doses [[33], [34], [35], [36], [37]]. While the LNT framework assumes that biological effects scale proportionally with dose, increasing evidence suggests that low-dose responses may involve qualitatively distinct mechanisms. Our findings are consistent with this view, showing that low- and high-dose irradiation are associated with different molecular responses in the developing cerebellum and with distinct tumor-associated transcriptional programs.

Together with recent observations in the irradiated non-human primate hippocampus [38], these findings support the concept that low-dose irradiation is not simply an attenuated version of high-dose exposure but represents a biologically distinct response. Rather than contradicting the linear no-threshold model, these data suggest that similar dose–risk relationships may arise through different molecular mechanisms.

The main limitations of this study are that molecular analyses were performed on bulk cerebellar tissue and established tumors, precluding cell type–specific resolution and direct mechanistic inference, and that findings obtained in the *Ptch1⁺^/^⁻* model may not fully recapitulate sporadic human MB. Future integration of single-cell, spatial, and functional approaches will be required to define the cellular basis of the dose-specific molecular programs identified here.

The identification of distinct dose-associated molecular programs raises the possibility that radiation dose influences not only tumor risk but also tumor biology, potentially generating different therapeutic vulnerabilities while providing a biological framework for future risk assessment models.

In conclusion, rather than simply increasing the probability of malignant transformation, radiation dose appears to establish distinct biological trajectories that progressively emerge during cerebellar development and remain reflected in radiation-induced MBs. The increasing systems-level concordance across independent molecular layers further suggests that higher radiation doses elicit progressively coordinated biological remodeling, providing a mechanistic framework linking developmental radiation exposure to tumor heterogeneity and offering a foundation for biologically informed risk assessment and dose-specific therapeutic strategies. Collectively, these findings support a model in which radiation dose shapes distinct biological contexts during cerebellar development that remain reflected in radiation-induced MBs, providing a framework that extends beyond DNA damage alone to explain dose-dependent tumor evolution.

## Disclosures

The authors declare no potential conflicts of interest.

## Funding

This work was supported by DISCOVER project, funded by the Euratom Programme PIANOFORTE (Grant Agreement No 101061037).

## Data sharing statement

Research data are stored in an institutional repository and will be shared on request to the corresponding author.

## CRediT authorship contribution statement

**Fratini Emiliano:** Writing – original draft, Visualization, Software, Methodology, Investigation, Formal analysis, Data curation. **De Stefano Ilaria:** Resources, Methodology. **Leonardi Simona:** Validation, Methodology, Investigation. **Pasquali Emanuela:** Validation, Methodology, Investigation. **Casciati Arianna:** Writing – review & editing, Methodology, Investigation, Data curation. **Tanno Barbara:** Methodology, Investigation, Data curation. **Antonelli Francesca:** Writing – review & editing, Methodology, Investigation, Data curation. **Duchrow Lukas:** Software, Methodology, Investigation. **Cemmi Alessia:** Resources, Methodology. **Di Sarcina Ilaria:** Resources, Methodology. **Mancuso Mariateresa:** Resources, Project administration. **Kadhim Munira:** Writing – review & editing. **Moertl Simone:** Writing – review & editing, Writing – original draft, Supervision, Project administration, Funding acquisition, Conceptualization. **Pazzaglia Simonetta:** Writing – review & editing, Writing – original draft, Supervision, Project administration, Funding acquisition, Conceptualization.

## Declaration of competing interest

The authors declare that they have no known competing financial interests or personal relationships that could have appeared to influence the work reported in this paper.

## Appendix Supplementary materials

Image, application 1

Image, image 2 dummy alt text

Image, image 3 dummy alt text

Image, image 4 dummy alt text

Image, image 5 dummy alt text

Image, application 6

Image, application 7

Image, application 8

Image, application 9

Image, application 10

Image, application 11

Image, application 12

Image, application 13

Image, application 14
