# Supplementary material for: Multi-omics characterization of radiation-induced cerebellar remodeling and tumorigenic transcriptional programs
Source: Neoplasia. 2026 Jun 29;79:101333. doi: 10.1016/j.neo.2026.101333 (PMC13330529; doi:10.1016/j.neo.2026.101333)
Supplement: Supplementary file 1 [file mmc1.docx]

## **Supplementary Material and Methods**

**Animals**

All animal procedures were conducted in accordance with European Community Council Directive 2010/63/EU. The study protocol was approved by the ENEA Animal Ethics Committee and authorized by the Italian Ministry of Health (authorization no. XXX/XXX-XX). To promote animal welfare, environmental enrichment, including nesting paper and mouse tubes, was provided in all cages. All experiments were performed in compliance with ARRIVE guidelines.

**Irradiation procedure**

Irradiation was performed at the Calliope facility (ENEA Casaccia Research Center, Rome, Italy), a pool-type gamma irradiation plant equipped with a ^60Co radioisotopic source array with a mean photon energy of 1.25 MeV [1]. At postnatal day 2 (P2), mice were immobilized in a custom honeycomb polystyrene support to minimize movement and ensure homogeneous frontal irradiation. Animals were positioned inside dedicated lead bunkers designed to achieve very low dose-rate conditions. Irradiation was carried out at doses of 2 Gy and 0.1 Gy, delivered at dose rates of 116 mGy/min and 7.33 mGy/min, respectively, with all values expressed in water. Sham-irradiated controls underwent identical handling and positioning procedures without exposure to ionizing radiation. The absorbed dose was determined using Fricke dosimetry according to ASTM Standard Practice (51026:2023, Standard Practice for Using the Fricke Dosimetry System, [www.astm.org](http://www.astm.org/)).

**Sample collection and histopathology**

Eight biological replicates per experimental group (SHAM, 0.1 Gy, and 2 Gy) were used for molecular analyses. Cerebella were collected at 1 and 6 weeks after irradiation, snap-frozen in liquid nitrogen, and stored at −80°C until further processing.

For tumorigenesis, mice were monitored daily up to 60 weeks. Animals were euthanized upon signs of morbidity, including severe weight loss, paralysis, ruffled fur, or reduced activity, and subjected to necropsy. Whole brains and any visible masses were collected. Brain tissues were fixed in 10% neutral buffered formalin, embedded in paraffin, sectioned at 4 μm thickness, and stained using standard histological procedures. Medulloblastoma diagnosis was confirmed by microscopic examination.

**Reduced Representation Bisulfite Sequencing (RRBS)**

Fresh frozen cerebellum tissue was ground into a powder in liquid nitrogen and total DNA was isolated with the Wizard Genomic DNA kit according to the manufactures protocol (Promega, Germany). For quality control an agarose gel electrophoresis was performed and 5 µg per sample were analyzed by Reduced Bisulfite Sequencing (RRBS) at Active Motif.

Data analysis steps included a FASTQ Processing where Single-end 75 nucleotide sequencing reads are generated by Illumina sequencing. By Bismark Alignment, reads are mapped to the genome using Bismark and Bowtie 2 allowing for no mismatches (-N 0) and a seed substring length of 20 (-L 20). Alignment information of the remaining reads is stored in BAM files. Methylation at CpG sites is then extracted from the BAM files using Bismark and reported in bedGraph, coverage, and CpG report formats. Using MethylKit Analysis CpG reports from the Bismark alignment are processed with the methylKit R package, and only CpG sites covered with at least 10 reads are retained for the downstream analyses. The average methylation at each chromosome and at each annotated transcript are determined on a per-sample basis. For the region-based analysis, the genome is tiled into 1 kilobase regions and significantly differentially methylated tiles are identified (methylation difference > 20%). For pairwise comparisons a Fisher’s exact test is used to identify differentially methylated sites, while a Chi-squared test is used if there are more than two groups. Differential methylation was defined as a change in CpG methylation of >20% combined with a false discovery rate (FDR)-adjusted q-value < 0.05. Analyses were performed using four biological replicates per treatment condition [2].

**Proteomic analysis**

Fresh frozen cerebellum tissue was ground into a powder in liquid nitrogen and lysed in iST lysis buffer (Protein Tech, Germany). 5 µg protein were further processed with the Preomics preparation kit (Protein Tech, Germany) according to the manufactures protocol.

For mass spectrometry, peptides were loaded on Evotips (one Evotip for each injection). They were placed in the Evosep (Evosep, Odense, Denmark) autosampler until analysis. The 20 samples per day whisper method employing a 27-minute gradient with solvents A (0.1% FA, H_2_O) and B (0.1% FA, MeCN) was chosen and a 15 cm column (PepSep C18, 1.9µm beads, 75µm ID) used for separation of peptides. The samples were measured on a TIMS quadrupole TOF mass spectrometer (Bruker tims TOF Ultra2) in DIA-PASEF mode with TIMS on. The DIA-PASEF method covered a mass range from 300 to 1,250 m/z and a mobility range from 0.64 to 1.30 1/ko. Ion-charge control (ICC 2.0) was switched on with 95% target of the total ion chromatogram (TIC). Precursor peptides were isolated with 27 variable windows. Collision energy for 0.6 1/ko was set to 20 and for 1.6 1/ko to 50. Estimated cycle time was 1.28 seconds. For data analysis files were processed with Spectronaut (Version 19, Biognosys) as direct DIA (data independent analysis against a SwissProt mouse database (Release 2020_02, 17061 sequences) using BSG factory settings for Pulsar search. For DIA analysis, default settings were applied. For quantification, precursor filtering was set on Q value, with cross run normalization. Quantity MS level was set to MS2; Quantity type was area, and protein quantification is based on the summed-up peptide intensities. Protein-group specific peptides were allowed for quantification. Among the quantified proteins, the expression of proteins was considered as significantly changed by a q-value <0.05, identification by at least two unique peptides and a deregulation of 1.5- or 0,6-fold.

**RNA sequencing (RNA-seq)**

Total RNA was isolated from MB tissues using the miRNeasy Micro Kit (QIAGEN, Hilden, Germany). For each experimental condition, four pooled samples were generated, each comprising three MBs. RNA integrity and quality were assessed by capillary electrophoresis, and RNA concentration was measured using a Qubit fluorometer (Thermo Fisher Scientific Inc., Waltham, MA, USA).

Libraries for stranded total RNA sequencing were prepared using the Stranded Total RNA Prep with Ligation Kit (Illumina Inc., San Diego, CA, USA). Library quality was evaluated using an automated electrophoresis system (Agilent Technologies Inc., Santa Clara, CA, USA). RNA sequencing was performed using sequencing-by-synthesis (SBS) technology on the SURFSeq 5000 platform (GeneMind, Shenzhen, Guangdong, China).

Standard RNA-sequencing data analysis was performed using the nf-core/rnaseq pipeline (version 3.19.0, Tungsten Turtle). Briefly, raw read quality was assessed using FastQC, followed by adapter and low-quality base trimming with Trim Galore. Filtered reads were aligned to the reference genome using STAR, and transcript-level quantification was performed using Salmon. Quality control metrics and alignment statistics were aggregated and visualized using MultiQC. The resulting gene count matrices were subsequently used for downstream analyses, including differential gene expression analysis.

**Differential gene expression and functional enrichment**

Differentially expressed genes (DEGs) between irradiated and SHAM groups were identified from transcriptomic data using DESeq2. Raw gene counts were normalized using the median-of-ratios method, and dispersion estimates were calculated according to the DESeq2 statistical model. Differential expression was assessed using a negative binomial generalized linear model, and p-values were adjusted for multiple testing using the Benjamini–Hochberg procedure. Genes with an adjusted p-value ≤ 0.05 and an absolute log2 fold change (|log2FC|) ≥ 1.5 were considered significantly differentially expressed.

Functional enrichment analysis was performed using g:profiler (<http://biit.cs.ut.ee/gprofiler/gost>, accessed on 5-23 December 2025) querying Gene ontology categories (Biological Process, BP, Molecular Function, MF; Cellular Component, CC), as well as KEGG, Reactome (REACT) and CORUM protein complexes databases. To visualize and interpret the enrichment results, the EnrichmentMap app within Cytoscape (version 3.10.4) was employed. Specifically, enrichment data from BP, MF, KEGG, and REACTOME were integrated into a network where nodes represent enriched terms and edges represent gene overlap between sets. This approach allowed for the clustering of redundant functional categories into major biological themes, facilitating a more intuitive interpretation of the results.

**Network analysis**

Network analysis was conducted using Cytoscape (version 3.10.4, University of California, San Diego, California) [3]. For RNA-RNA interaction analysis, murine RNA–RNA interaction subnetworks were generated by selecting interactions involving DEGs specifically modulated in 0.1 Gy– or 2 Gy–induced MBs, as well as DEGs commonly modulated across all radiation-induced MBs. These subnetworks were derived from a database of experimentally validated RNA–RNA interactions (NPInter V5). Protein–protein interaction (PPI) networks were constructed using *stringApp* to import functional associations and physical interactions from the Search Tool for the Retrieval of Interacting Genes (STRING) database [4]. A minimum confidence score of ≥ 0.4 was applied, and the maximum number of additional interactors was set to zero.

**Overlap between omics datasets**

To investigate common and platform-specific biological processes across the methylome, proteome, and transcriptome, g:Profiler enrichment analyses were conducted using the complete sets of significantly radiation-affected methylation sites, differentially expressed proteins, and differentially expressed transcripts identified following exposure to 0.1 Gy or 2.0 Gy. For the methylomic and proteomic datasets, significantly altered features from the 1- and 6-week time points were combined for each dose. All significantly enriched functional categories with a multiple testing–corrected q-value < 0.05 were retained for further analysis. Overlaps among enriched terms obtained from Gene Ontology (GO) Biological Process (BP), Molecular Function (MF), and Cellular Component (CC) terms, KEGG pathways, Reactome pathways, transcription factor (TF) targets, Wiki pathways (WP) and CORUM protein complexes were identified and visualized using Venn diagrams.

**Quantitative real-time PCR (qPCR)**

Total RNA was isolated from cerebella collected 1 week after irradiation (n=24), with a miRNeasy Mini Kit (QIAGEN, Hilden, Germany). Two micrograms of RNA was reverse transcribed using High-Capacity cDNA Reverse Transcription Kit (Applied Biosystems, Foster City, CA, USA), and qPCR reactions were performed in triplicate from each biological replicate in the QuantStudio™ 5 Real-Time PCR System (Applied Biosystems) using the Power up SYBR® Green PCR Master Mix (Applied Biosystems). Relative quantification was carried out using the ΔΔCt method, with Glyceraldehyde-3-phosphate (*Gapdh*) as the endogenous housekeeping control. The oligonucleotide primers used for quantitative RT-PCR of genes analyzed are listed in Table 1.

**Table I: Primers Sequence**

| **Gene** | **Forward Primer** | **Reverse Primer** |
| --- | --- | --- |
| *Math1* | 5′-AAAGCCCTGCAGAAGGAGCTAGAA-3′ | 5′-AACACCTTTCCAAAGAGAACGCCC-3′ |
| *Zic-1* | 5′-AAGCAGAAGATGCGGACTG-3′ | 5′-GCTTGCACTTCATCCTTTGG-3′ |
| *NeuN* | 5′-CGAGAACGGTGGAACTTTGAC-3′ | 5′-CAGGGCTCAGGTAGACCTTG-3′ |
| *Tub-.III* | 5′-CTGGGCGAGCTGATCC-3’′ | 5′-GGTGAGGCTGCAGCA-3′ |
| *Calb1* | 5′-TCCGCAAGCATGCACAGA-3′ | 5′-AGGGTGGGTTGGAAATGAACT-3′ |
| *Gfap* | 5′-GAAGCTCCGCCTGGT-3′ | 5’-CCAGCTTCGAGCCAA-3′ |
| *S-100* | 5’-TCATGGAGGACCTGC-3′ | 5’-CAGCATCATACACTC-3′ |
| *Tnf-a* | 5’CAGACCCTCACACTCAGATCATCTT-3’ | 5′TCGTAGCAAACCACCAAGTGG -3 |
| *Il-6* | 5’CAAGTCGGAGGCTTAATTACACATG -3 | 5 ′AGAAAAGAGTTGTGCAATGGCA -3 ′ |
| *Tgf-b* | 5’GTGGAAATCAACGGGATCAG-3’ | 5’-ACTTCCAACCCAGGTCCTTC-3’ |
| *Gli-1* | *5′-AGGACCTGGAGAGAGAGGAGAA-3* | *5′-CCAGCGGCAGTCTGTCTCA-3′* |
| *Cxcl-5* | *5’-CGTAACTCCAAAAATTAATCCCAAA-3’* | *5’-CGAGTGCATTCCGCTTAGCT-3’* |
| *CD31* | *5’-CCAAAGCCAGTAGCATCATGG-3’* | *5’-GGATGGTGAAGTTGGCTACAG-3’* |
| *Vegf* | 5’-CTCCTGTAACGATGAAGCCCT-3′ | 5′-GCTGTAGGAAGCTCATCTCTC-3′ |
| *Gapdh* | 5′-CATGGCCTTCCGTGTTCCTA-3′ | 5’-GCGGCACGTCAGATCCA-3′ |

**References**

1. Baccaro S, et al. Gamma irradiation Calliope facility at ENEA-Casaccia Research Centre (Rome, Italy). ***Fusion Technol Nucl Saf Secur Dept Casaccia Res Centre*.** 2019:49.
2. Akalin A, Kormaksson M, Li S, Garrett-Bakelman FE, Figueroa ME, Melnick A, Mason CE. MethylKit: a comprehensive R package for the analysis of genome-wide DNA methylation profiles. *Genome Biology*, 2012;13,R87.
3. Shannon P, Markiel A, Ozier O, et al. Cytoscape: a software environment for integrated models of biomolecular interaction networks. ***Genome Res*.** 2003;13:2498-2504.
4. Doncheva NT, Morris JH, Gorodkin J, Jensen LJ. Cytoscape StringApp: network analysis and visualization of proteomics data. ***J Proteome Res*.** 2019;18:623-632.
