# Supplementary figures and images for: Multi-omics characterization of radiation-induced cerebellar remodeling and tumorigenic transcriptional programs
Source: Neoplasia. 2026 Jun 29;79:101333. doi: 10.1016/j.neo.2026.101333 (PMC13330529; doi:10.1016/j.neo.2026.101333)

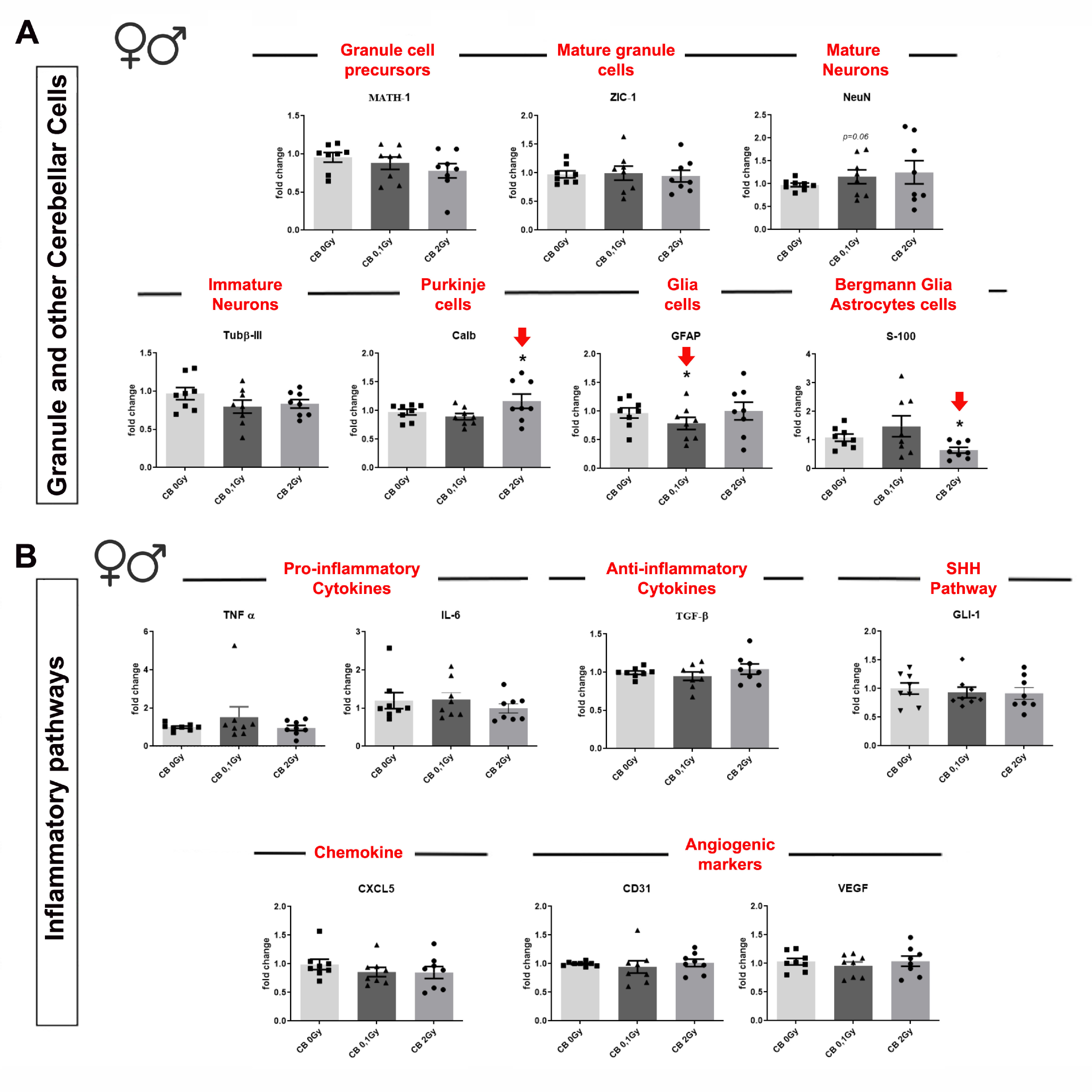

Supplement: Supplementary file 2 [file mmc2.jpg]

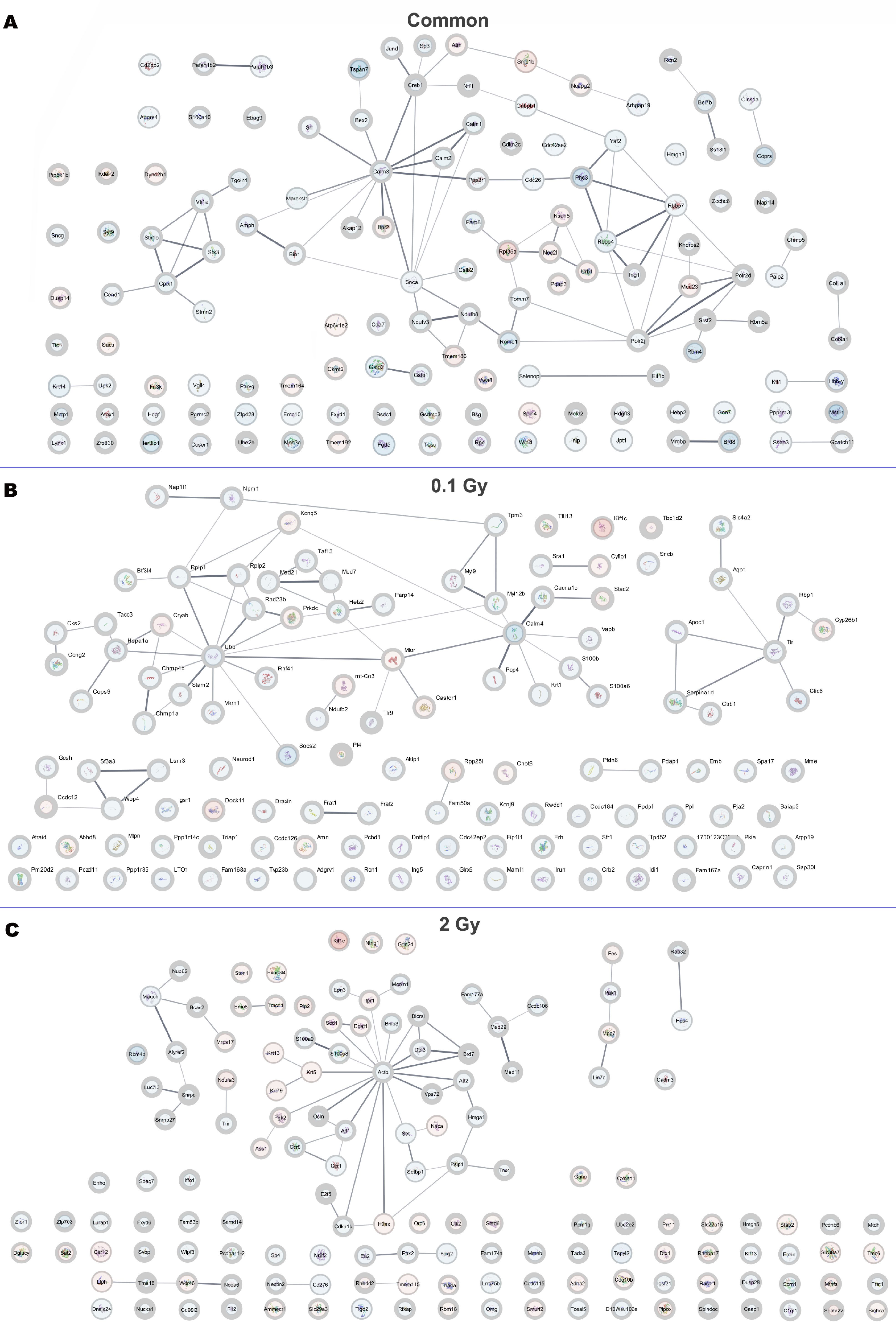

Supplement: Supplementary file 3 [file mmc3.jpg]

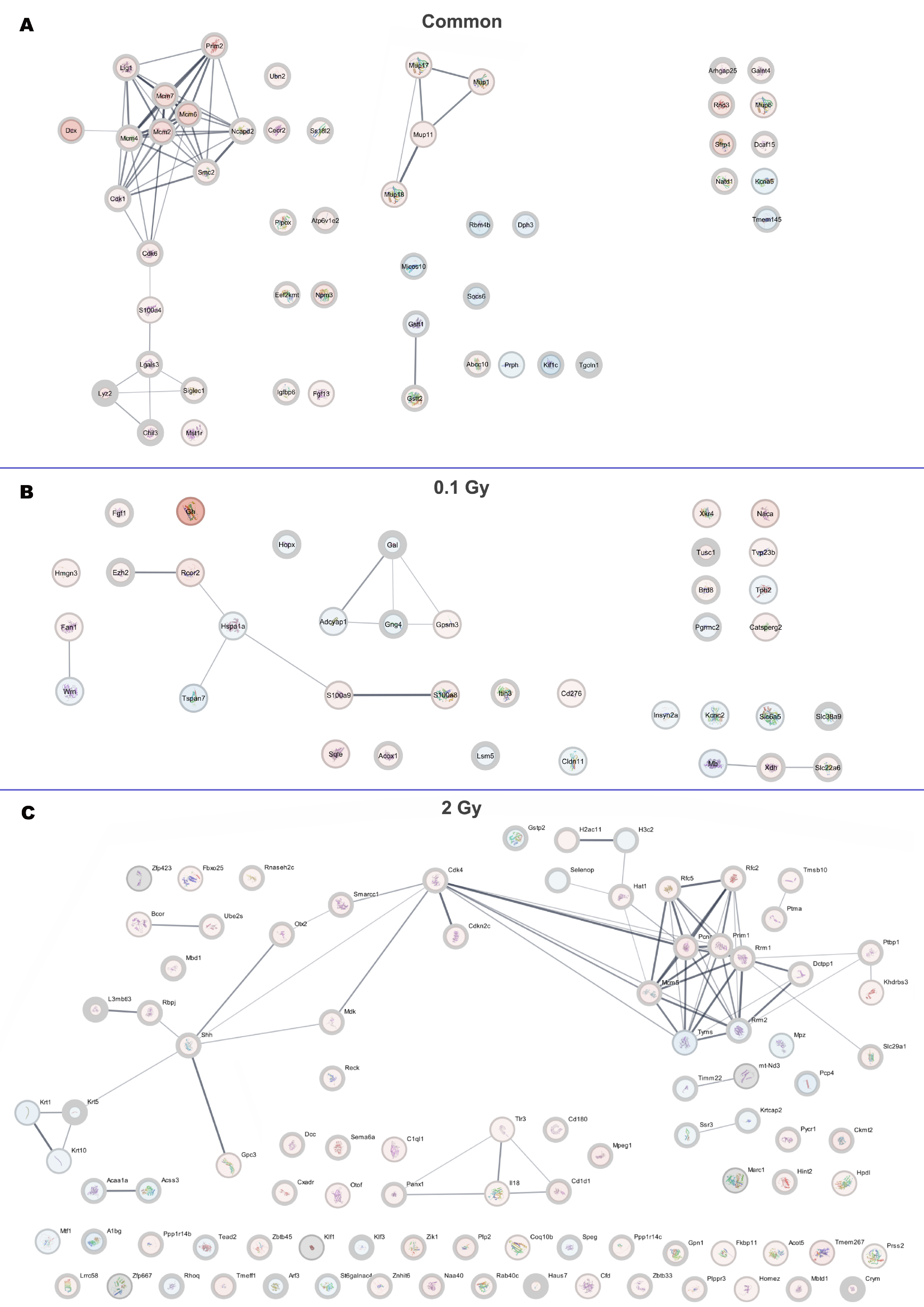

Supplement: Supplementary file 4 [file mmc4.jpg]

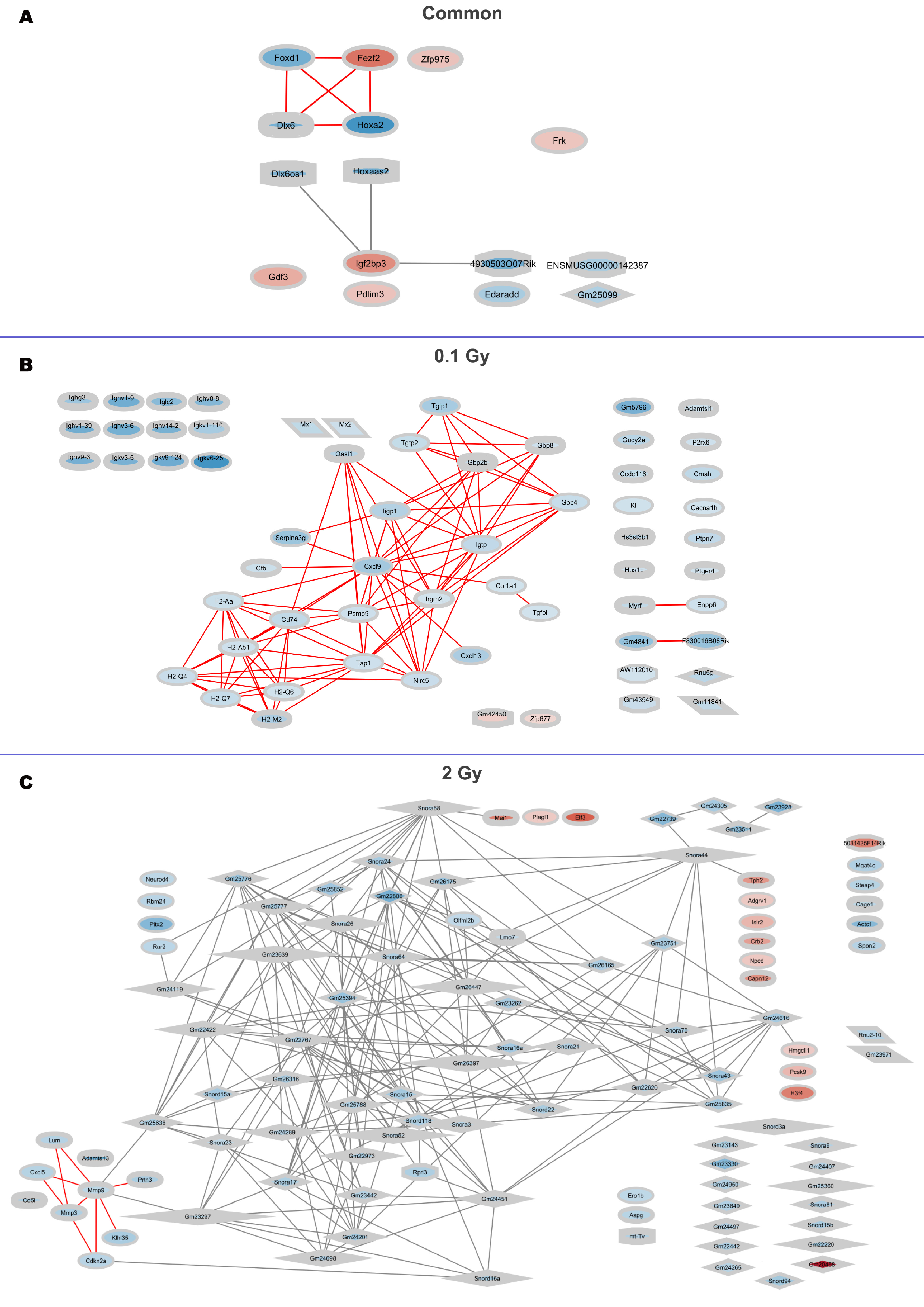

Supplement: Supplementary file 5 [file mmc5.jpg]
