# Supplementary material for: Multi-omics characterization of radiation-induced cerebellar remodeling and tumorigenic transcriptional programs
Source: Neoplasia. 2026 Jun 29;79:101333. doi: 10.1016/j.neo.2026.101333 (PMC13330529; doi:10.1016/j.neo.2026.101333)
Supplement: Supplementary file 6 [file mmc6.pdf]

Common 1 week - Figure 4B

<table>
<tr><th>Cluster</th><th>DESCRIPTION</th><th>Name</th><th>FDR_qvalue</th><th>Genes</th></tr>
<tr><td rowspan="72">Ion Transport and Membrane Excitability</td><td>CLEC7A (DECTIN-1) INDUCES NFAT ACTIVATION</td><td>REAC:R-MMU-5607763</td><td>3.84E-05</td><td>CALM2|CALM3|PPP3R1|CALM1</td></tr>
<tr><td>CALCINEURIN ACTIVATES NFAT</td><td>REAC:R-MMU-2025928</td><td>7.64E-05</td><td>CALM2|CALM3|PPP3R1|CALM1</td></tr>
<tr><td>NEGATIVE REGULATION OF RYANODINE-SENSITIVE CALCIUM-RELEASE CHANNEL ACTIVITY</td><td>GO:0060315</td><td>2.28E-04</td><td>CALM2|CALM3|CALM1</td></tr>
<tr><td>PLATELET CALCIUM HOMEOSTASIS</td><td>REAC:R-MMU-418360</td><td>3.97E-04</td><td>CALM2|CALM3|ITPR2|SRI|CALM1</td></tr>
<tr><td>SODIUM/CALCIUM EXCHANGERS</td><td>REAC:R-MMU-425561</td><td>5.27E-04</td><td>CALM2|CALM3|SRI|CALM1</td></tr>
<tr><td>RENIN SECRETION</td><td>KEGG:04924</td><td>7.45E-04</td><td>CALM2|CALM3|ITPR2|CREB1|PPP3R1|CALM1</td></tr>
<tr><td>REDUCTION OF CYTOSOLIC CA++ LEVELS</td><td>REAC:R-MMU-418359</td><td>7.57E-04</td><td>CALM2|CALM3|SRI|CALM1</td></tr>
<tr><td>ION HOMEOSTASIS</td><td>REAC:R-MMU-5578775</td><td>7.58E-04</td><td>CALM2|FXYD1|CALM3|ITPR2|SRI|CALM1</td></tr>
<tr><td>PRESYNAPTIC ENDOCYTOSIS</td><td>GO:0140238</td><td>7.74E-04</td><td>CALM2|CALM3|AMPH|BIN1|SNCG|SNCA|CALM1</td></tr>
<tr><td>TYPE 3 METABOTROPIC GLUTAMATE RECEPTOR BINDING</td><td>GO:0031800</td><td>8.24E-04</td><td>CALM2|CALM3|CALM1</td></tr>
<tr><td>ADENYLATE CYCLASE ACTIVATOR ACTIVITY</td><td>GO:0010856</td><td>8.24E-04</td><td>CALM2|CALM3|CALM1</td></tr>
<tr><td>NEGATIVE REGULATION OF CALCIUM ION EXPORT ACROSS PLASMA MEMBRANE</td><td>GO:1905913</td><td>9.11E-04</td><td>CALM2|CALM3|CALM1</td></tr>
<tr><td>OOCYTE MEIOSIS</td><td>KEGG:04114</td><td>0.001051597</td><td>CALM2|CALM3|SMC1B|CDC26|ITPR2|PPP3R1|CALM1</td></tr>
<tr><td>POST NMDA RECEPTOR ACTIVATION EVENTS</td><td>REAC:R-MMU-438064</td><td>0.001053528</td><td>CALM2|CALM3|CREB1|CALM1</td></tr>
<tr><td>CREB1 PHOSPHORYLATION THROUGH THE ACTIVATION OF CAMKII/CAMKK/CAMKIV CASCASDE</td><td>REAC:R-MMU-442729</td><td>0.001439762</td><td>CALM2|CALM3|CALM1</td></tr>
<tr><td>CAMK IV-MEDIATED PHOSPHORYLATION OF CREB</td><td>REAC:R-MMU-111932</td><td>0.001439762</td><td>CALM2|CALM3|CALM1</td></tr>
<tr><td>NEGATIVE REGULATION OF CALCIUM ION TRANSMEMBRANE TRANSPORT</td><td>GO:1903170</td><td>0.001680888</td><td>CALM2|CALM3|BIN1|PPP3R1|CALM1</td></tr>
<tr><td>ESTROGEN SIGNALING PATHWAY</td><td>KEGG:04915</td><td>0.00205586</td><td>CALM2|CALM3|ITPR2|EBAG9|CREB1|KRT14|CALM1</td></tr>
<tr><td>REGULATION OF CALCIUM ION EXPORT ACROSS PLASMA MEMBRANE</td><td>GO:1905912</td><td>0.002268409</td><td>CALM2|CALM3|CALM1</td></tr>
<tr><td>CAM-PDE 1 ACTIVATION</td><td>REAC:R-MMU-111957</td><td>0.002862953</td><td>CALM2|CALM3|CALM1</td></tr>
<tr><td>ACTIVATION OF RAC1 DOWNSTREAM OF NMDARS</td><td>REAC:R-MMU-9619229</td><td>0.002862953</td><td>CALM2|CALM3|CALM1</td></tr>
<tr><td>REGULATION OF CARDIAC MUSCLE CONTRACTION</td><td>GO:0055117</td><td>0.003299882</td><td>CALM2|FXYD1|CALM3|BIN1|SRI|CALM1</td></tr>
<tr><td>NEGATIVE REGULATION OF HIGH VOLTAGE-GATED CALCIUM CHANNEL ACTIVITY</td><td>GO:1901842</td><td>0.004520798</td><td>CALM2|CALM3|CALM1</td></tr>
<tr><td>NITRIC-OXIDE SYNTHASE REGULATOR ACTIVITY</td><td>GO:0030235</td><td>0.004564107</td><td>CALM2|CALM3|CALM1</td></tr>
<tr><td>GLUCAGON SIGNALING PATHWAY</td><td>KEGG:04922</td><td>0.004836973</td><td>CALM2|CALM3|ITPR2|CREB1|PPP3R1|CALM1</td></tr>
<tr><td>LONG-TERM POTENTIATION</td><td>KEGG:04720</td><td>0.005660553</td><td>CALM2|CALM3|ITPR2|PPP3R1|CALM1</td></tr>
<tr><td>AMPHETAMINE ADDICTION</td><td>KEGG:05031</td><td>0.006535687</td><td>CALM2|CALM3|CREB1|PPP3R1|CALM1</td></tr>
<tr><td>CARDIAC MUSCLE CONTRACTION</td><td>GO:0060048</td><td>0.006920745</td><td>CALM2|FXYD1|CALM3|BIN1|PPP1R13L|SRI|CALM1</td></tr>
<tr><td>ADENYLATE CYCLASE REGULATOR ACTIVITY</td><td>GO:0010854</td><td>0.009707755</td><td>CALM2|CALM3|CALM1</td></tr>
<tr><td>TITIN BINDING</td><td>GO:0031432</td><td>0.009707755</td><td>CALM2|CALM3|CALM1</td></tr>
<tr><td>CYCLASE ACTIVATOR ACTIVITY</td><td>GO:0010853</td><td>0.009707755</td><td>CALM2|CALM3|CALM1</td></tr>
<tr><td>REGULATION OF STRIATED MUSCLE CONTRACTION</td><td>GO:0006942</td><td>0.011106111</td><td>CALM2|FXYD1|CALM3|BIN1|SRI|CALM1</td></tr>
<tr><td>CELLULAR SENESCENCE</td><td>KEGG:04218</td><td>0.011742788</td><td>CALM2|CALM3|RBBP4|ITPR2|PPP3R1|ATM|CALM1</td></tr>
<tr><td>TETRAHYDROBIOPTERIN (BH4) SYNTHESIS, RECYCLING, SALVAGE AND REGULATION</td><td>REAC:R-MMU-1474151</td><td>0.011818117</td><td>CALM2|CALM3|CALM1</td></tr>
<tr><td>REGULATION OF HIGH VOLTAGE-GATED CALCIUM CHANNEL ACTIVITY</td><td>GO:1901841</td><td>0.012569033</td><td>CALM2|CALM3|CALM1</td></tr>
<tr><td>REGULATION OF RYANODINE-SENSITIVE CALCIUM-RELEASE CHANNEL ACTIVITY</td><td>GO:0060314</td><td>0.012569033</td><td>CALM2|CALM3|CALM1</td></tr>
<tr><td>NEGATIVE REGULATION OF VOLTAGE-GATED CALCIUM CHANNEL ACTIVITY</td><td>GO:1901386</td><td>0.012569033</td><td>CALM2|CALM3|CALM1</td></tr>
<tr><td>NITRIC-OXIDE SYNTHASE ACTIVITY</td><td>GO:0004517</td><td>0.013298631</td><td>CALM2|CALM3|CALM1</td></tr>
<tr><td>NEGATIVE REGULATION OF CALCIUM ION TRANSPORT</td><td>GO:0051926</td><td>0.015767368</td><td>CALM2|CALM3|BIN1|PPP3R1|CALM1</td></tr>
<tr><td>ION TRANSPORT BY P-TYPE ATPASES</td><td>REAC:R-MMU-936837</td><td>0.016410795</td><td>CALM2|FXYD1|CALM3|SRI|CALM1</td></tr>
<tr><td>MITOCHONDRION-ENDOPLASMIC RETICULUM MEMBRANE TETHERING</td><td>GO:1990456</td><td>0.018787022</td><td>CALM2|CALM3|CALM1</td></tr>
<tr><td>AUTOPHAGOSOME MEMBRANE DOCKING</td><td>GO:0016240</td><td>0.018787022</td><td>CALM2|CALM3|CALM1</td></tr>
<tr><td>REGULATION OF CARDIAC MUSCLE CELL ACTION POTENTIAL</td><td>GO:0098901</td><td>0.020139262</td><td>CALM2|CALM3|BIN1|CALM1</td></tr>
<tr><td>NEGATIVE REGULATION OF CATION TRANSMEMBRANE TRANSPORT</td><td>GO:1904063</td><td>0.022199253</td><td>CALM2|CALM3|BIN1|PPP3R1|CALM1</td></tr>
<tr><td>CALCIUM CHANNEL INHIBITOR ACTIVITY</td><td>GO:0019855</td><td>0.022880259</td><td>CALM2|CALM3|CALM1</td></tr>
<tr><td>ACTIVATION OF CA-PERMEABLE KAINATE RECEPTOR</td><td>REAC:R-MMU-451308</td><td>0.022948086</td><td>CALM2|CALM3|CALM1</td></tr>
<tr><td>IONOTROPIC ACTIVITY OF KAINATE RECEPTORS</td><td>REAC:R-MMU-451306</td><td>0.022948086</td><td>CALM2|CALM3|CALM1</td></tr>
<tr><td>ENOS ACTIVATION</td><td>REAC:R-MMU-203615</td><td>0.022948086</td><td>CALM2|CALM3|CALM1</td></tr>
<tr><td>CARDIAC CONDUCTION</td><td>REAC:R-MMU-5576891</td><td>0.026125365</td><td>CALM2|FXYD1|CALM3|ITPR2|SRI|CALM1</td></tr>
<tr><td>NEGATIVE REGULATION OF CALCIUM ION TRANSMEMBRANE TRANSPORTER ACTIVITY</td><td>GO:1901020</td><td>0.026743919</td><td>CALM2|CALM3|CALM1</td></tr>
<tr><td>NEGATIVE REGULATION OF RELEASE OF SEQUESTERED CALCIUM ION INTO CYTOSOL</td><td>GO:0051280</td><td>0.026743919</td><td>CALM2|CALM3|CALM1</td></tr>
<tr><td>REGULATION OF CALCIUM ION TRANSMEMBRANE TRANSPORT</td><td>GO:1903169</td><td>0.02823109</td><td>CALM2|CALM3|BIN1|SNCA|PPP3R1|SRI|CALM1</td></tr>
<tr><td>ADENYLATE CYCLASE BINDING</td><td>GO:0008179</td><td>0.02901234</td><td>CALM2|CALM3|AKAP12</td></tr>
<tr><td>G PROTEIN-COUPLED GLUTAMATE RECEPTOR BINDING</td><td>GO:0035256</td><td>0.02901234</td><td>CALM2|CALM3|CALM1</td></tr>
<tr><td>ION CHANNEL INHIBITOR ACTIVITY</td><td>GO:0008200</td><td>0.029492966</td><td>CALM2|CALM3|LYNX1|CALM1</td></tr>
<tr><td>NEGATIVE REGULATION OF MONOATOMIC ION TRANSMEMBRANE TRANSPORT</td><td>GO:0034766</td><td>0.032933557</td><td>CALM2|CALM3|BIN1|PPP3R1|CALM1</td></tr>
<tr><td>PHOSPHATIDYLINOSITOL SIGNALING SYSTEM</td><td>KEGG:04070</td><td>0.033239979</td><td>CALM2|CALM3|ITPR2|PIP5K1B|CALM1</td></tr>
<tr><td>ACTIVATION OF NMDA RECEPTORS AND POSTSYNAPTIC EVENTS</td><td>REAC:R-MMU-442755</td><td>0.033942678</td><td>CALM2|CALM3|CREB1|CALM1</td></tr>
<tr><td>TRANSPORTER INHIBITOR ACTIVITY</td><td>GO:0141110</td><td>0.036029068</td><td>CALM2|CALM3|LYNX1|CALM1</td></tr>
<tr><td>CHANNEL INHIBITOR ACTIVITY</td><td>GO:0016248</td><td>0.036029068</td><td>CALM2|CALM3|LYNX1|CALM1</td></tr>
<tr><td>CALCIUM ION EXPORT ACROSS PLASMA MEMBRANE</td><td>GO:1990034</td><td>0.036643185</td><td>CALM2|CALM3|CALM1</td></tr>
<tr><td>DETECTION OF CALCIUM ION</td><td>GO:0005513</td><td>0.036643185</td><td>CALM2|CALM3|CALM1</td></tr>
<tr><td>ALDOSTERONE SYNTHESIS AND SECRETION</td><td>KEGG:04925</td><td>0.041995103</td><td>CALM2|CALM3|ITPR2|CREB1|CALM1</td></tr>
<tr><td>CYCLASE REGULATOR ACTIVITY</td><td>GO:0010851</td><td>0.044304049</td><td>CALM2|CALM3|CALM1</td></tr>
<tr><td>KINASE REGULATOR ACTIVITY</td><td>GO:0019207</td><td>0.045204676</td><td>CALM2|CDKN2C|CALM3|MOB3A|TESC|PARP8|GSTP1|CALM1</td></tr>
<tr><td>CALCIUM-DEPENDENT PROTEIN BINDING</td><td>GO:0048306</td><td>0.045306853</td><td>CALM2|CALM3|S100A10|STMN2|CALM1</td></tr>
<tr><td>STRIATED MUSCLE CONTRACTION</td><td>GO:0006941</td><td>0.04663437</td><td>CALM2|FXYD1|CALM3|BIN1|PPP1R13L|SRI|CALM1</td></tr>
<tr><td>POSITIVE REGULATION OF SEQUESTERING OF CALCIUM ION</td><td>GO:0051284</td><td>0.048685286</td><td>CALM2|CALM3|CALM1</td></tr>
<tr><td>GLYCOGEN BREAKDOWN (GLYCOGENOLYSIS)</td><td>REAC:R-MMU-70221</td><td>0.049757643</td><td>CALM2|CALM3|CALM1</td></tr>
<tr><td>PKA ACTIVATION</td><td>REAC:R-MMU-163615</td><td>0.049757643</td><td>CALM2|CALM3|CALM1</td></tr>
<tr><td>METABOLISM OF NITRIC OXIDE: NOS3 ACTIVATION AND REGULATION</td><td>REAC:R-MMU-202131</td><td>0.049757643</td><td>CALM2|CALM3|CALM1</td></tr>
<tr><td>PKA-MEDIATED PHOSPHORYLATION OF CREB</td><td>REAC:R-MMU-111931</td><td>0.049757643</td><td>CALM2|CALM3|CALM1</td></tr>
<tr><td rowspan="12">Synaptic Vescicle Exocytosis and Neurotransmitter Release</td><td>VESICLE FUSION</td><td>GO:0006906</td><td>0.004530309</td><td>STX3|SNCA|CHMP5|VTI1A|SYT9|STX1B|CPLX1</td></tr>
<tr><td>ORGANELLE MEMBRANE FUSION</td><td>GO:0090174</td><td>0.005050731</td><td>STX3|SNCA|CHMP5|VTI1A|SYT9|STX1B|CPLX1</td></tr>
<tr><td>SYNAPTIC VESICLE CYCLE</td><td>GO:0099504</td><td>0.005609493</td><td>STX3|AMPH|BIN1|SNCG|SNCA|SYT9|PPP3R1|STX1B|CPLX1</td></tr>
<tr><td>SNARE BINDING</td><td>GO:0000149</td><td>0.010523968</td><td>STX3|SNCA|VTI1A|SYT9|STX1B|CPLX1</td></tr>
<tr><td>EXOCYTIC INSERTION OF NEUROTRANSMITTER RECEPTOR TO POSTSYNAPTIC MEMBRANE</td><td>GO:0098967</td><td>0.012569033</td><td>STX3|STX1B|CPLX1</td></tr>
<tr><td>SYNAPTIC VESICLE FUSION TO PRESYNAPTIC ACTIVE ZONE MEMBRANE</td><td>GO:0031629</td><td>0.017326788</td><td>STX3|SYT9|STX1B|CPLX1</td></tr>
<tr><td>VESICLE FUSION TO PLASMA MEMBRANE</td><td>GO:0099500</td><td>0.020139262</td><td>STX3|SYT9|STX1B|CPLX1</td></tr>
<tr><td>SYNAPTIC VESICLE MEMBRANE ORGANIZATION</td><td>GO:0048499</td><td>0.030604462</td><td>STX3|SYT9|STX1B|CPLX1</td></tr>
<tr><td>SIGNAL RELEASE FROM SYNAPSE</td><td>GO:0099643</td><td>0.035784802</td><td>STX3|MCTP1|SNCG|SNCA|SYT9|STX1B|CPLX1</td></tr>
<tr><td>NEUROTRANSMITTER SECRETION</td><td>GO:0007269</td><td>0.035784802</td><td>STX3|MCTP1|SNCG|SNCA|SYT9|STX1B|CPLX1</td></tr>
<tr><td>ORGANELLE FUSION</td><td>GO:0048284</td><td>0.040145697</td><td>STX3|SNCA|CHMP5|VTI1A|SYT9|STX1B|CPLX1</td></tr>
<tr><td>MEMBRANE FUSION</td><td>GO:0061025</td><td>0.04838491</td><td>STX3|SNCA|CHMP5|VTI1A|SYT9|STX1B|CPLX1</td></tr>
<tr><td rowspan="7">Cellular Trafficking and Vescicle-Mediated Transport</td><td>ESTABLISHMENT OF LOCALIZATION IN CELL</td><td>GO:0051649</td><td>1.99E-04</td><td>CALM2|KIF1C|CALM3|AMPH|WIPI1|DYNC2H1|SYT9|ATM|RBM4|MCTP1|KDELR2|BIN1|SNCG|RBM8A|SRI|STX3|UBE2B|HMGN3|SNCA|TSPAN7|CHMP5|PPP3R1|ROMO1|IER3IP1|ING1|VTI1A|STX1B|CPLX1|CALM1</td></tr>
<tr><td>VESICLE-MEDIATED TRANSPORT</td><td>GO:0016192</td><td>5.25E-04</td><td>STX3|CALM2|KIF1C|CALM3|AMPH|WIPI1|SNCA|TSPAN7|CHMP5|SYT9|PPP3R1|IER3IP1|CDC42SE2|MST1R|MCFD2|MCTP1|KDELR2|BIN1|S100A10|SNCG|VTI1A|STX1B|CPLX1|CALM1</td></tr>
<tr><td>CELLULAR LOCALIZATION</td><td>GO:0051641</td><td>0.001446851</td><td>CALM2|KIF1C|CALM3|AMPH|ITPR2|WIPI1|DYNC2H1|SYT9|ATM|RBM4|MCFD2|MCTP1|KDELR2|BIN1|S100A10|BSG|SNCG|RBM8A|SRI|STX3|UBE2B|HMGN3|HDGF|SNCA|TSPAN7|CHMP5|PPP3R1|ROMO1|IER3IP1|EMC10|TOMM7|ING1|COL1A1|TESC|VTI1A|STX1B|CPLX1|CALM1</td></tr>
<tr><td>ESTABLISHMENT OF LOCALIZATION</td><td>GO:0051234</td><td>0.002727499</td><td>CALM2|KIF1C|CALM3|AMPH|ITPR2|WIPI1|DYNC2H1|CREB1|SYT9|ATM|HBB-Y|RBM4|ATOX1|MST1R|MCFD2|MCTP1|KDELR2|BIN1|S100A10|SNCG|RBM8A|SRI|STX3|FXYD1|UBE2B|HMGN3|SNCA|TSPAN7|CHMP5|PPP3R1|ROMO1|IER3IP1|CDC42SE2|EMC10|TOMM7|PGRMC2|ATP6V1E2|CLNS1A|ING1|COL1A1|TESC|VTI1A|STX1B|CPLX1|CALM1</td></tr>
<tr><td>TRANSPORT</td><td>GO:0006810</td><td>0.007868125</td><td>CALM2|KIF1C|CALM3|AMPH|ITPR2|WIPI1|DYNC2H1|CREB1|SYT9|HBB-Y|RBM4|ATOX1|MST1R|MCFD2|MCTP1|KDELR2|BIN1|S100A10|SNCG|RBM8A|SRI|STX3|FXYD1|HMGN3|SNCA|TSPAN7|CHMP5|PPP3R1|ROMO1|IER3IP1|CDC42SE2|TOMM7|PGRMC2|ATP6V1E2|CLNS1A|ING1|COL1A1|TESC|VTI1A|STX1B|CPLX1|CALM1</td></tr>
<tr><td>EXPORT FROM CELL</td><td>GO:0140352</td><td>0.011092716</td><td>STX3|CALM2|FXYD1|CALM3|HMGN3|CREB1|SNCA|SYT9|IER3IP1|RBM4|MCTP1|S100A10|SNCG|STX1B|CPLX1|SRI|CALM1</td></tr>
<tr><td>LOCALIZATION</td><td>GO:0051179</td><td>0.016187089</td><td>CALM2|KIF1C|CALM3|AMPH|ITPR2|WIPI1|DYNC2H1|CREB1|SYT9|ATM|HBB-Y|RBM4|ATOX1|MST1R|MCFD2|MCTP1|KDELR2|BIN1|S100A10|BSG|SNCG|RBM8A|SRI|STX3|FXYD1|UBE2B|HMGN3|HDGF|SNCA|TSPAN7|CHMP5|PPP3R1|ROMO1|IER3IP1|CDC42SE2|EMC10|TOMM7|PGRMC2|ATP6V1E2|CLNS1A|ING1|COL1A1|TESC|VTI1A|STX1B|CPLX1|CALM1</td></tr>
</table>

<table>
<tr><td></td><td>REGULATION OF SIGNALING</td><td>GO:0023051</td><td>0.044395915</td><td>CALM2|CALM3|SPIN4|NOC2L|RBBP4|DYNC2H1|CREB1|SYT9|ATM|RBM4|MST1R|RBBP7|CALB2|AKAP12|MCTP1|SNCG|PAIP2|SRI|STX3|UBE2B|LYNX1|HMGN3|SNCA|CHMP5|VGLL4|PPP3R1|CDC42SE2|JUND|ARHGAP19|ING1|COL1A1|STX1B|CPLX1|GSTP1|CALM1</td></tr>
<tr><td rowspan="7">Calcium Homeostasis and Ion Regulation</td><td>CALCIUM ION BINDING</td><td>GO:0005509</td><td>0.001881415</td><td>CALM2|CALM3|ITPR2|SNCA|SYT9|PPP3R1|RCN2|ADGRE4|CALB2|MCFD2|MCTP1|S100A10|TESC|SRI|CALM1</td></tr>
<tr><td>REGULATION OF MONOATOMIC CATION TRANSMEMBRANE TRANSPORT</td><td>GO:1904062</td><td>0.008887382</td><td>CALM2|FXYD1|CALM3|BIN1|TESC|SNCA|PPP3R1|SRI|CALM1</td></tr>
<tr><td>REGULATION OF MONOATOMIC ION TRANSMEMBRANE TRANSPORT</td><td>GO:0034765</td><td>0.021155927</td><td>CALM2|FXYD1|CALM3|BIN1|TESC|SNCA|PPP3R1|SRI|CALM1</td></tr>
<tr><td>CELLULAR HOMEOSTASIS</td><td>GO:0019725</td><td>0.040352132</td><td>CALM2|FXYD1|CALM3|MARCKSL1|HMGN3|ITPR2|SNCA|RBM4|ATOX1|CALB2|CLNS1A|COL1A1|TESC|SRI|CALM1</td></tr>
<tr><td>INTRACELLULAR CHEMICAL HOMEOSTASIS</td><td>GO:0055082</td><td>0.041527733</td><td>CALM2|FXYD1|CALM3|MARCKSL1|HMGN3|ITPR2|SNCA|RBM4|ATOX1|CALB2|COL1A1|TESC|SRI|CALM1</td></tr>
<tr><td>INTRACELLULAR CALCIUM ION HOMEOSTASIS</td><td>GO:0006874</td><td>0.043457155</td><td>CALM2|FXYD1|CALB2|CALM3|MARCKSL1|ITPR2|SNCA|SRI|CALM1</td></tr>
<tr><td rowspan="4">Protein Binding-Mediated Positive Regulation</td><td>POSITIVE REGULATION OF CELLULAR PROCESS</td><td>GO:0048522</td><td>2.77E-04</td><td>SPIN4|NOC2L|DYNC2H1|NCAPG2|ATM|GABPB1|RBBP7|AKAP12|BIN1|S100A10|BSG|STMN2|NRF1|SRI|SSBP3|STX3|FXYD1|UBE2B|HDGF|PPP1R13L|SNCA|VGLL4|PPP3R1|PAFAH1B2|JUND|ING1|COL1A1|TESC|YAF2|STX1B|GSTP1|CALM1|CALM2|CALM3|MARCKSL1|AMPH|RBBP4|WIPI1|CREB1|SP3|SYT9|RBM4|MST1R|CALB2|NSUN5|PAIP2|SS18L1|HMGN3|ROMO1|IER3IP1|MED23|TMEM164|EMC10|TOMM7|MRGBP|BCL7B</td></tr>
<tr><td>PROTEIN BINDING</td><td>GO:0005515</td><td>4.01E-04</td><td>KIF1C|SMC1B|SPIN4|NOC2L|ITPR2|DYNC2H1|NCAPG2|PIP5K1B|TMEM192|SACS|ATM|GABPB1|NAP1L4|PAFAH1B3|ATOX1|SRSF2|RBBP7|AKAP12|POLR2J|BIN1|S100A10|BSG|SNCG|CEND1|STMN2|CD2BP2|NRF1|POLR2D|SRI|SSBP3|SELENOP|STX3|FXYD1|UBE2B|LYNX1|HDGF|PPP1R13L|HDGFL3|SNCA|VGLL4|PPP3R1|KHDRBS2|COA7|CDC42SE2|COL9A1|PAFAH1B2|RCN2|PGRMC2|CDKN2C|JUND|ING1|COL1A1|TTC1|TESC|PARP8|VTI1A|BEX2|YAF2|STX1B|CPLX1|GSTP1|PARVG|CALM1|CALM2|CALM3|MARCKSL1|AMPH|RBBP4|WIPI1|CREB1|SP3|SYT9|KRT14|GSTP2|HBB-Y|RBM4|COPRS|PHC3|MST1R</td></tr>
<tr><td>POSITIVE REGULATION OF BIOLOGICAL PROCESS</td><td>GO:0048518</td><td>0.001973846</td><td>SPIN4|NOC2L|DYNC2H1|NCAPG2|ATM|GABPB1|RBBP7|AKAP12|BIN1|S100A10|BSG|STMN2|NRF1|SRI|SSBP3|STX3|FXYD1|UBE2B|HDGF|PPP1R13L|SNCA|VGLL4|PPP3R1|PAFAH1B2|JUND|ING1|COL1A1|TESC|YAF2|STX1B|GSTP1|CALM1|CALM2|CALM3|MARCKSL1|AMPH|RBBP4|WIPI1|CREB1|SP3|SYT9|RBM4|MST1R|CALB2|NSUN5|PAIP2|SS18L1|HMGN3|ROMO1|IER3IP1|MED23|TMEM164|EMC10|TOMM7|MRGBP|BCL7B</td></tr>
<tr><td>BINDING</td><td>GO:0005488</td><td>0.039235052</td><td>KIF1C|SMC1B|SPIN4|NOC2L|ITPR2|DYNC2H1|NCAPG2|PIP5K1B|TMEM192|SACS|ATM|GABPB1|NAP1L4|PAFAH1B3|ATOX1|SRSF2|RBBP7|AKAP12|POLR2J|BIN1|S100A10|BSG|SNCG|CEND1|STMN2|CD2BP2|NRF1|POLR2D|SRI|SSBP3|SELENOP|STX3|FXYD1|UBE2B|LYNX1|HDGF|PPP1R13L|HDGFL3|SNCA|VGLL4|PPP3R1|KHDRBS2|COA7|CDC42SE2|COL9A1|PAFAH1B2|RCN2|PGRMC2|CDKN2C|JUND|ING1|COL1A1|TTC1|TESC|PARP8|VTI1A|BEX2|YAF2|STX1B|CPLX1|GSTP1|PARVG|CALM1|CALM2|CALM3|MARCKSL1|AMPH|RBBP4|WIPI1|CREB1|SP3|SYT9|KRT14|GSTP2|HBB-Y|RBM4|COPRS|PHC3|MST1R|ADGRE4|CALB2|MCFD2|MCTP1|KDELR2|VWA8|FN3K|CKMT2|NSUN5|GPATCH11|GSDMC3|RBM8A|PAIP2|ZCCHC8|ZNF830|HEBP2|SS18L1|RPE|EIF1B|HMGN3|ZNF428|MOB3A|FGD5</td></tr>
<tr><td></td><td>VESICLE-MEDIATED TRANSPORT IN SYNAPSE</td><td>GO:0099003</td><td>4.25E-06</td><td>STX3|CALM2|CALM3|AMPH|SNCA|TSPAN7|SYT9|PPP3R1|BIN1|SNCG|STX1B|CPLX1|CALM1</td></tr>
<tr><td></td><td>HISTONE BINDING</td><td>GO:0042393</td><td>0.001158087</td><td>RBBP7|SPIN4|ING1|NOC2L|RBBP4|NCAPG2|SNCA|NAP1L4|COPRS|PHC3</td></tr>
<tr><td></td><td>CUPROUS ION BINDING</td><td>GO:1903136</td><td>0.006820745</td><td>SNCG|SNCA|ATOX1</td></tr>
<tr><td></td><td>TRANSCRIPTIONAL REGULATION BY E2F6</td><td>REAC:R-MMU-8953750</td><td>0.008825521</td><td>RBBP7|RBBP4|YAF2|PHC3</td></tr>
</table>

0.1Gy 1 week - Figure 4C

<table>
<tr><th>Cluster</th><th>DESCRIPTION</th><th>Name</th><th>FDR_qvalue</th><th>Genes</th></tr>
<tr><td rowspan="4">Regulation of Cell Differentiation</td><td>PROTEIN BINDING</td><td>GO:0005515</td><td>1.78006E-05</td><td>DOCK11|CRYAB|PF4|STAC2|TTLL13|CASTOR1|TBC1D2|AMN|PRKDC|CYFIP1|CNOT6|MTOR|PFDN6|SAP30L|MTPN|STAM2|ING5|PCP4|AQP1|TPD52|WBP4|CHMP1A|PJA2|MED21|NEUROD1|MYL9|GCSH|VAPB|EMB|PDAP1|CHMP4B|TRIAP1|TTR|RAD23B|ARPP19|RWDD1|SPA17|MAML1|TPM3|DNTTIP1|FIP1L1|TAF13|PKIA|TLR9|PCBD1|ADGRV1|ILRUN|S100B|PARP14|PM20D2|DRAXIN|NAP1L1|RNF41|MYL12B|PPP1R35|CRB2|IGSF1|AKIP1|CACNA1C|KRT1|BAIAP3|NPM1|SNCB|S100A6|CKS2|MME|PDZD11|SLC4A2|ATRAID|HSPA1A|CDC42EP2|KCNJ9|PPL|UBB|CLIC6|SOCS2</td></tr>
<tr><td>REGULATION OF CELL DIFFERENTIATION</td><td>GO:0045595</td><td>0.010522523</td><td>MME|PF4|ATRAID|MAML1|PRKDC|CYFIP1|MTOR|TLR9|SOCS2|ADGRV1|S100B|ING5|PCP4|CAPRIN1|DRAXIN|CYP26B1|NAP1L1|SRA1|RNF41|RBP1|CRB2|NEUROD1</td></tr>
<tr><td>POSITIVE REGULATION OF BIOLOGICAL PROCESS</td><td>GO:0048518</td><td>0.022390477</td><td>DOCK11|PF4|STAC2|CASTOR1|PRKDC|CYFIP1|CNOT6|MTOR|SAP30L|MTPN|ING5|PCP4|AQP1|TPD52|PJA2|MED21|NEUROD1|VAPB|TRIAP1|MAML1|TAF13|TLR9|PCBD1|ADGRV1|S100B|PARP14|NAP1L1|RNF41|PPP1R35|CRB2|CACNA1C|KRT1|BAIAP3|NPM1|MME|ATRAID|HSPA1A|CDC42EP2|UBB|SOCS2|CAPRIN1|CYP26B1|SRA1|FAM168A|MED7|CTRB1|COPS9|SFR1|HELZ2</td></tr>
<tr><td>POSITIVE REGULATION OF CELL DIFFERENTIATION</td><td>GO:0045597</td><td>0.041001828</td><td>MME|ATRAID|MAML1|PRKDC|CYFIP1|MTOR|TLR9|SOCS2|S100B|PCP4|CAPRIN1|CYP26B1|NAP1L1|CRB2|NEUROD1</td></tr>
<tr><td rowspan="2">Membrane Organelle Assembly</td><td>REGULATION OF ORGANELLE ASSEMBLY</td><td>GO:1902115</td><td>0.022779677</td><td>CAPRIN1|CHMP1A|HSPA1A|PPP1R35|CNOT6|MTOR|NPM1|CHMP4B</td></tr>
<tr><td>MEMBRANELESS ORGANELLE ASSEMBLY</td><td>GO:0140694</td><td>0.029418181</td><td>CAPRIN1|CHMP1A|HSPA1A|PPP1R35|PRKDC|MYL9|CNOT6|LSM3|NPM1|CHMP4B</td></tr>
<tr><td rowspan="2">Enzime and Molecular Function Regulation</td><td>ENZYME REGULATOR ACTIVITY</td><td>GO:0030234</td><td>0.000785406</td><td>DOCK11|CKS2|ARPP19|PDZD11|TBC1D2|CDC42EP2|DNTTIP1|FIP1L1|PKIA|SOCS2|PPP1R14C|RPLP1|ADGRV1|CCNG2|APOC1|SERPINA1D|CALM4|PPP1R35|CRB2|NPM1</td></tr>
<tr><td>MOLECULAR FUNCTION REGULATOR ACTIVITY</td><td>GO:0098772</td><td>0.001923953</td><td>DOCK11|CKS2|PF4|PDZD11|CASTOR1|TBC1D2|CDC42EP2|SOCS2|PPP1R14C|RPLP1|CCNG2|APOC1|SERPINA1D|CALM4|CAPRIN1|TTR|RAD23B|ARPP19|DNTTIP1|FIP1L1|PKIA|ADGRV1|PPP1R35|CRB2|IGSF1|NPM1</td></tr>
<tr><td></td><td>POSITIVE REGULATION OF PLASMA MEMBRANE BOUNDED CELL PROJECTION ASSEMBLY</td><td>GO:0120034</td><td>0.021373924</td><td>DOCK11|AQP1|PPP1R35|CDC42EP2|CYFIP1|MTOR</td></tr>
</table>

2Gy 1 week - Figure 4D

<table>
<tr><th>Cluster</th><th>DESCRIPTION</th><th>Name</th><th>FDR_qvalue</th><th>Genes</th></tr>
<tr><td rowspan="2">Chromatin Remodeling and Organization</td><td>CHROMATIN ORGANIZATION</td><td>GO:0006325</td><td>0.012865321</td><td>BRD7|TADA3|PSIP1|NUCKS1|HMGA1|H2AX|PAK1|SET|DPF3|HMGN5|ATF2|BICRAL|ACTB|SETBP1|VPS72|TSPYL2</td></tr>
<tr><td>ATP-DEPENDENT CHROMATIN REMODELING</td><td>KEGG:03082</td><td>0.017349149</td><td>ACTB|VPS72|BRD7|H2AX|DPF3|BICRAL</td></tr>
<tr><td></td><td>TRANSCRIPTION COACTIVATOR ACTIVITY</td><td>GO:0003713</td><td>0.01720914</td><td>NACA|NCOA6|BRD7|TADA3|PSIP1|MTDH|NUCKS1|HMGA1</td></tr>
</table>
