# Supplementary material for: Multi-omics characterization of radiation-induced cerebellar remodeling and tumorigenic transcriptional programs
Source: Neoplasia. 2026 Jun 29;79:101333. doi: 10.1016/j.neo.2026.101333 (PMC13330529; doi:10.1016/j.neo.2026.101333)
Supplement: Supplementary file 7 [file mmc7.pdf]

Common 6 weeks - Figure 5B

<table>
<tr><th>Cluster</th><th>DESCRIPTION</th><th>Name</th><th>FDR_qvalue</th><th>Genes</th></tr>
<tr><td rowspan="55">DNA Replication and Cell-Cycle Regulation</td><td>DNA REPLICATION</td><td>KEGG:03030</td><td>7.98935E-09</td><td>MCM6|MCM2|MCM7|MCM4|PRIM2|LIG1</td></tr>
<tr><td>G1 TO S CELL CYCLE CONTROL</td><td>WP:WP413</td><td>3.56355E-08</td><td>MCM6|MCM2|CDK1|MCM7|MCM4|PRIM2|CDK6</td></tr>
<tr><td>PREMEIOTIC DNA REPLICATION</td><td>GO:0006279</td><td>1.44223E-07</td><td>MCM6|MCM2|MCM7|MCM4</td></tr>
<tr><td>DNA REPLICATION INITIATION</td><td>GO:0006270</td><td>3.70371E-06</td><td>MCM6|MCM2|MCM7|MCM4|PRIM2</td></tr>
<tr><td>DOUBLE-STRAND BREAK REPAIR VIA BREAK-INDUCED REPLICATION</td><td>GO:0000727</td><td>4.72682E-06</td><td>MCM6|MCM2|MCM7|MCM4</td></tr>
<tr><td>ACTIVATION OF THE PRE-REPLICATIVE COMPLEX</td><td>REAC:R-MMU-68962</td><td>5.52067E-06</td><td>MCM6|MCM2|MCM7|MCM4|PRIM2</td></tr>
<tr><td>SINGLE-STRANDED DNA HELICASE ACTIVITY</td><td>GO:0017116</td><td>2.21508E-05</td><td>MCM6|MCM2|MCM7|MCM4</td></tr>
<tr><td>CELL CYCLE</td><td>KEGG:04110</td><td>7.19674E-05</td><td>MCM6|MCM2|CDK1|MCM7|MCM4|CDK6</td></tr>
<tr><td>ATP-DEPENDENT ACTIVITY</td><td>GO:0140657</td><td>0.000228677</td><td>MCM6|MCM2|MCM7|MCM4|CECR2|ATP6V1E2|SMC2|ABCC10|KIF1C</td></tr>
<tr><td>DNA REPLICATION</td><td>GO:0006260</td><td>0.000314729</td><td>MCM6|MCM2|CDK1|MCM7|MCM4|PRIM2|LIG1</td></tr>
<tr><td>MITOTIC G1 PHASE AND G1/S TRANSITION</td><td>REAC:R-MMU-453279</td><td>0.000393425</td><td>MCM6|MCM2|MCM7|MCM4|PRIM2|CDK6</td></tr>
<tr><td>NUCLEAR DNA REPLICATION</td><td>GO:0033260</td><td>0.000547271</td><td>MCM6|MCM2|MCM7|MCM4</td></tr>
<tr><td>SINGLE-STRANDED DNA BINDING</td><td>GO:0003697</td><td>0.000595062</td><td>MCM6|MCM2|MCM7|MCM4|SMC2</td></tr>
<tr><td>ATP-DEPENDENT ACTIVITY, ACTING ON DNA</td><td>GO:0008094</td><td>0.00064617</td><td>MCM6|MCM2|MCM7|MCM4|CECR2</td></tr>
<tr><td>ACTIVATION OF ATR IN RESPONSE TO REPLICATION STRESS</td><td>REAC:R-MMU-176187</td><td>0.000807655</td><td>MCM6|MCM2|MCM7|MCM4</td></tr>
<tr><td>DNA HELICASE ACTIVITY</td><td>GO:0003678</td><td>0.000884091</td><td>MCM6|MCM2|MCM7|MCM4</td></tr>
<tr><td>CELL CYCLE DNA REPLICATION</td><td>GO:0044786</td><td>0.000935512</td><td>MCM6|MCM2|MCM7|MCM4</td></tr>
<tr><td>CATALYTIC ACTIVITY, ACTING ON DNA</td><td>GO:0140097</td><td>0.001205949</td><td>MCM6|MCM2|MCM7|MCM4|CECR2|LIG1</td></tr>
<tr><td>ATP HYDROLYSIS ACTIVITY</td><td>GO:0016887</td><td>0.001365941</td><td>MCM6|MCM2|MCM7|MCM4|SMC2|ABCC10|KIF1C</td></tr>
<tr><td>DNA REPLICATION PRE-INITIATION</td><td>REAC:R-MMU-69002</td><td>0.002078408</td><td>MCM6|MCM2|MCM7|MCM4|PRIM2</td></tr>
<tr><td>G1/S TRANSITION</td><td>REAC:R-MMU-69206</td><td>0.002935721</td><td>MCM6|MCM2|MCM7|MCM4|PRIM2</td></tr>
<tr><td>MITOTIC DNA REPLICATION</td><td>GO:1902969</td><td>0.002949817</td><td>MCM6|MCM2|MCM4</td></tr>
<tr><td>SYNTHESIS OF DNA</td><td>REAC:R-MMU-69239</td><td>0.004614889</td><td>MCM6|MCM2|MCM7|MCM4|PRIM2</td></tr>
<tr><td>DNA-TEMPLATED DNA REPLICATION</td><td>GO:0006261</td><td>0.00544679</td><td>MCM6|MCM2|MCM7|MCM4|PRIM2</td></tr>
<tr><td>DNA REPLICATION</td><td>REAC:R-MMU-69306</td><td>0.006179535</td><td>MCM6|MCM2|MCM7|MCM4|PRIM2</td></tr>
<tr><td>CATALYTIC ACTIVITY</td><td>GO:0003824</td><td>0.006225164</td><td>MCM6|MCM2|MCM7|MCM4|CECR2|SMC2|ABCC10|KIF1C|LIG1|SFRP1|RND3|LYZ2|CDK6|CHIL3|ARHGAP25|GALNT4|CDK1|MST1R|FGF13|GSTT2|PIPOX|EEF2KMT|GSTT1|SOCS6</td></tr>
<tr><td>ATP BINDING</td><td>GO:0005524</td><td>0.008056452</td><td>MCM6|MCM2|CDK1|MCM7|MST1R|MCM4|SMC2|ABCC10|KIF1C|LIG1|CDK6</td></tr>
<tr><td>CARBOHYDRATE DERIVATIVE BINDING</td><td>GO:0097367</td><td>0.008161425</td><td>MCM6|MCM2|MCM7|MCM4|SMC2|ABCC10|KIF1C|LIG1|SFRP1|RND3|CDK6|CHIL3|CDK1|MST1R</td></tr>
<tr><td>ADENYL RIBONUCLEOTIDE BINDING</td><td>GO:0032559</td><td>0.010410407</td><td>MCM6|MCM2|CDK1|MCM7|MST1R|MCM4|SMC2|ABCC10|KIF1C|LIG1|CDK6</td></tr>
<tr><td>S PHASE</td><td>REAC:R-MMU-69242</td><td>0.012994851</td><td>MCM6|MCM2|MCM7|MCM4|PRIM2</td></tr>
<tr><td>ORC1 REMOVAL FROM CHROMATIN</td><td>REAC:R-MMU-68949</td><td>0.013192538</td><td>MCM6|MCM2|MCM7|MCM4</td></tr>
<tr><td>COMPREHENSIVE IL 17A SIGNALING</td><td>WP:WP5242</td><td>0.016634279</td><td>MCM6|MCM2|MCM7|MCM4</td></tr>
<tr><td>CELL CYCLE, MITOTIC</td><td>REAC:R-MMU-69278</td><td>0.017633061</td><td>MCM6|MCM2|CDK1|MCM7|MCM4|SMC2|PRIM2|CDK6</td></tr>
<tr><td>ADENYL NUCLEOTIDE BINDING</td><td>GO:0030554</td><td>0.01961641</td><td>MCM6|MCM2|CDK1|MCM7|MST1R|MCM4|SMC2|ABCC10|KIF1C|LIG1|CDK6</td></tr>
<tr><td>MITOTIC CELL CYCLE PROCESS</td><td>GO:1903047</td><td>0.022743381</td><td>MCM6|MCM2|CDK1|MCM4|SMC2|NCAPD2|KCNA5|CDK6</td></tr>
<tr><td>ASSEMBLY OF THE PRE-REPLICATIVE COMPLEX</td><td>REAC:R-MMU-68867</td><td>0.023420415</td><td>MCM6|MCM2|MCM7|MCM4</td></tr>
<tr><td>G2/M CHECKPOINTS</td><td>REAC:R-MMU-69481</td><td>0.023852615</td><td>MCM6|MCM2|CDK1|MCM7|MCM4</td></tr>
<tr><td>DNA METABOLIC PROCESS</td><td>GO:0006259</td><td>0.025940805</td><td>MCM6|MCM2|CDK1|MCM7|MCM4|SMC2|GSTT1|PRIM2|LIG1</td></tr>
<tr><td>NUCLEOTIDE BINDING</td><td>GO:0000166</td><td>0.028211272</td><td>MCM6|MCM2|MCM7|MCM4|SMC2|ABCC10|KIF1C|LIG1|RND3|CDK6|CDK1|MST1R|PIPOX</td></tr>
<tr><td>RIBONUCLEOSIDE TRIPHOSPHATE PHOSPHATASE ACTIVITY</td><td>GO:0017111</td><td>0.028800672</td><td>ARHGAP25|MCM6|MCM2|MCM7|MCM4|SMC2|ABCC10|KIF1C|RND3</td></tr>
<tr><td>CELL CYCLE PROCESS</td><td>GO:0022402</td><td>0.030225745</td><td>MCM6|MCM2|CDK1|MCM7|MCM4|SMC2|SFRP1|NCAPD2|KCNA5|CDK6</td></tr>
<tr><td>PURINE RIBONUCLEOSIDE TRIPHOSPHATE BINDING</td><td>GO:0035639</td><td>0.030445573</td><td>MCM6|MCM2|CDK1|MCM7|MST1R|MCM4|SMC2|ABCC10|KIF1C|LIG1|RND3|CDK6</td></tr>
<tr><td>NUCLEOSIDE PHOSPHATE BINDING</td><td>GO:1901265</td><td>0.031145661</td><td>MCM6|MCM2|MCM7|MCM4|SMC2|ABCC10|KIF1C|LIG1|RND3|CDK6|CDK1|MST1R|PIPOX</td></tr>
<tr><td>SWITCHING OF ORIGINS TO A POST-REPLICATIVE STATE</td><td>REAC:R-MMU-69052</td><td>0.035331252</td><td>MCM6|MCM2|MCM7|MCM4</td></tr>
<tr><td>MACROMOLECULAR CONFORMATION ISOMERASE ACTIVITY</td><td>GO:0120543</td><td>0.036038965</td><td>MCM6|MCM2|MCM7|MCM4|KIF1C</td></tr>
<tr><td>PURINE RIBONUCLEOTIDE BINDING</td><td>GO:0032555</td><td>0.040521144</td><td>MCM6|MCM2|CDK1|MCM7|MST1R|MCM4|SMC2|ABCC10|KIF1C|LIG1|RND3|CDK6</td></tr>
<tr><td>PYROPHOSPHATASE ACTIVITY</td><td>GO:0016462</td><td>0.041967929</td><td>ARHGAP25|MCM6|MCM2|MCM7|MCM4|SMC2|ABCC10|KIF1C|RND3</td></tr>
<tr><td>HYDROLASE ACTIVITY, ACTING ON ACID ANHYDRIDES, IN PHOSPHORUS-CONTAINING ANHYDRIDES</td><td>GO:0016818</td><td>0.042237962</td><td>ARHGAP25|MCM6|MCM2|MCM7|MCM4|SMC2|ABCC10|KIF1C|RND3</td></tr>
<tr><td>HYDROLASE ACTIVITY, ACTING ON ACID ANHYDRIDES</td><td>GO:0016817</td><td>0.042237962</td><td>ARHGAP25|MCM6|MCM2|MCM7|MCM4|SMC2|ABCC10|KIF1C|RND3</td></tr>
<tr><td>CELL CYCLE DNA REPLICATION INITIATION</td><td>GO:1902292</td><td>0.043504095</td><td>MCM2|MCM4</td></tr>
<tr><td>MITOTIC DNA REPLICATION INITIATION</td><td>GO:1902975</td><td>0.043504095</td><td>MCM2|MCM4</td></tr>
<tr><td>NUCLEAR CELL CYCLE DNA REPLICATION INITIATION</td><td>GO:1902315</td><td>0.043504095</td><td>MCM2|MCM4</td></tr>
<tr><td>RIBONUCLEOTIDE BINDING</td><td>GO:0032553</td><td>0.044138859</td><td>MCM6|MCM2|CDK1|MCM7|MST1R|MCM4|SMC2|ABCC10|KIF1C|LIG1|RND3|CDK6</td></tr>
<tr><td>HELICASE ACTIVITY</td><td>GO:0004386</td><td>0.046005115</td><td>MCM6|MCM2|MCM7|MCM4</td></tr>
<tr><td>CELL CYCLE</td><td>REAC:R-MMU-1640170</td><td>0.049355923</td><td>MCM6|MCM2|CDK1|MCM7|MCM4|SMC2|PRIM2|CDK6</td></tr>
</table>

0.1Gy 6 weeks - Figure 5C

<table>
<tr><th>Cluster</th><th>DESCRIPTION</th><th>Name</th><th>FDR_qvalue</th><th>Genes</th></tr>
<tr><td rowspan="3">Molecular Transport and Localization</td><td>LOCALIZATION</td><td>GO:0051179</td><td>0.006658744</td><td>WRN|XDH|MB|GH1|NACA|XKR4|S100A8|HMGN3|S100A9|TVP23B|EZH2|SLC22A6|SLC38A9|HSPA1A|GAL|KCNC2|PGRMC2|ADCYAP1|SLC6A5|TSPAN7</td></tr>
<tr><td>TRANSPORT</td><td>GO:0006810</td><td>0.008146211</td><td>XDH|MB|GH1|NACA|XKR4|S100A8|HMGN3|S100A9|TVP23B|SLC22A6|SLC38A9|HSPA1A|GAL|KCNC2|PGRMC2|ADCYAP1|SLC6A5|TSPAN7</td></tr>
<tr><td>ESTABLISHMENT OF LOCALIZATION</td><td>GO:0051234</td><td>0.021523321</td><td>XDH|MB|GH1|NACA|XKR4|S100A8|HMGN3|S100A9|TVP23B|SLC22A6|SLC38A9|HSPA1A|GAL|KCNC2|PGRMC2|ADCYAP1|SLC6A5|TSPAN7</td></tr>
<tr><td rowspan="3">Innate Immune Response</td><td>METAL SEQUESTRATION BY ANTIMICROBIAL PROTEINS</td><td>REAC:R-MMU-6799990</td><td>0.011563478</td><td>S100A8|S100A9</td></tr>
<tr><td>NEUTROPHIL AGGREGATION</td><td>GO:0070488</td><td>0.0322838</td><td>S100A8|S100A9</td></tr>
<tr><td>CELLULAR RESPONSE TO TOXIC SUBSTANCE</td><td>GO:0097237</td><td>0.046539552</td><td>KCNC2|MB|S100A8|S100A9</td></tr>
<tr><td></td><td>FLAP-STRUCTURED DNA BINDING</td><td>GO:0070336</td><td>0.000759797</td><td>FAN1|WRN</td></tr>
<tr><td></td><td>FAD BINDING</td><td>GO:0071949</td><td>0.013793439</td><td>SQLE|XDH|ACOX1</td></tr>
<tr><td></td><td>NITRITE REDUCTASE ACTIVITY</td><td>GO:0098809</td><td>0.033948692</td><td>XDH|MB</td></tr>
</table>

2Gy 6 weeks - Figure 5D

<table>
<tr><th>Cluster</th><th>DESCRIPTION</th><th>Name</th><th>FDR_qvalue</th><th>Genes</th></tr>
<tr><td rowspan="18">DNA Replication and Repair (S-Phase)</td><td>DNA REPLICATION</td><td>KEGG:03030</td><td>3.13502E-06</td><td>PCNA|RFC2|MCM5|RNASEH2C|PRIM1|RFC5</td></tr>
<tr><td>POLYMERASE SWITCHING</td><td>REAC:R-MMU-69091</td><td>0.000184196</td><td>PCNA|RFC2|PRIM1|RFC5</td></tr>
<tr><td>LEADING STRAND SYNTHESIS</td><td>REAC:R-MMU-69109</td><td>0.000184196</td><td>PCNA|RFC2|PRIM1|RFC5</td></tr>
<tr><td>LAGGING STRAND SYNTHESIS</td><td>REAC:R-MMU-69186</td><td>0.000664798</td><td>PCNA|RFC2|PRIM1|RFC5</td></tr>
<tr><td>G1 TO S CELL CYCLE CONTROL</td><td>WP:WP413</td><td>0.001645265</td><td>PCNA|MCM5|CDKN2C|CDK4|PRIM1</td></tr>
<tr><td>DNA STRAND ELONGATION</td><td>REAC:R-MMU-69190</td><td>0.001737239</td><td>PCNA|RFC2|PRIM1|RFC5</td></tr>
<tr><td>POLYMERASE SWITCHING ON THE C-STRAND OF THE TELOMERE</td><td>REAC:R-MMU-174411</td><td>0.002598693</td><td>PCNA|RFC2|PRIM1|RFC5</td></tr>
<tr><td>S PHASE</td><td>REAC:R-MMU-69242</td><td>0.006699341</td><td>UBE2S|PCNA|RFC2|MCM5|CDK4|PRIM1|RFC5</td></tr>
<tr><td>TELOMERE C-STRAND (LAGGING STRAND) SYNTHESIS</td><td>REAC:R-MMU-174417</td><td>0.007074472</td><td>PCNA|RFC2|PRIM1|RFC5</td></tr>
<tr><td>MISMATCH REPAIR</td><td>KEGG:03430</td><td>0.017607833</td><td>PCNA|RFC2|RFC5</td></tr>
<tr><td>SYNTHESIS OF DNA</td><td>REAC:R-MMU-69239</td><td>0.019223643</td><td>UBE2S|PCNA|RFC2|MCM5|PRIM1|RFC5</td></tr>
<tr><td>DNA REPLICATION</td><td>REAC:R-MMU-69306</td><td>0.026879713</td><td>UBE2S|PCNA|RFC2|MCM5|PRIM1|RFC5</td></tr>
<tr><td>TRANSLESION SYNTHESIS BY REV1</td><td>REAC:R-MMU-110312</td><td>0.03456676</td><td>PCNA|RFC2|RFC5</td></tr>
<tr><td>TRANSLESION SYNTHESIS BY POLI</td><td>REAC:R-MMU-5656121</td><td>0.03456676</td><td>PCNA|RFC2|RFC5</td></tr>
<tr><td>PCNA-DEPENDENT LONG PATCH BASE EXCISION REPAIR</td><td>REAC:R-MMU-5651801</td><td>0.03456676</td><td>PCNA|RFC2|RFC5</td></tr>
<tr><td>EXTENSION OF TELOMERES</td><td>REAC:R-MMU-180786</td><td>0.039781111</td><td>PCNA|RFC2|PRIM1|RFC5</td></tr>
<tr><td>TRANSLESION SYNTHESIS BY POLK</td><td>REAC:R-MMU-5655862</td><td>0.041788495</td><td>PCNA|RFC2|RFC5</td></tr>
<tr><td>DNA-TEMPLATED DNA REPLICATION</td><td>GO:0006261</td><td>0.042653983</td><td>PCNA|RFC2|MCM5|PRIM1|RFC5|RRM1</td></tr>
</table>

<table>
<tr><td rowspan="4">Molecular Binding and Interaction</td><td>BINDING</td><td>GO:0005488</td><td>0.003077191</td><td>ZIK1 | CKMT2 | PCNA | PLP2 | TMEFF1 | ZNF667 | MCM5 | DCC | OTX2 | C1QL1 | PRIM1 | SMARCC1 | MBTD1 | RFC5 | OTOF | GPC3 | PYCR1 | LRRC58 | ZBTB45 | PTMA | HINT2 | HAUS7 | PANX1 | CRYM | CDKN2C | ZNHIT6 | HOMEZ | RAB40C | KHDRBS3 | RRM1 | COQ10B | HPDL | BCOR | SHH | L3MBTL3 | PTBP1 | MAPKAPK5 | MDK | RECK | AFAP1 | CDK4 | KLHL18 | FBXO25 | TLR3 | MTARC1 | IL18 | CXADR | RBPJ | UBE2S | TMSB10 | ZBTB33 | ZNF423 | RFC2 | CD180 | H2AC11 | GPN1 | MBD1 | SEMA6A | TNS2 | HAT1 | ANTXR2 | NIP7 | KCTD16 | RCN1 | PRSS2 | DCTPP1 | SPEG | TIMM22 | SELENOP | KLF3 | AVIL | TIGD2 | H3C2 | KRT5 | ARF3 | MTF1 | PDZD11 | RHOQ | TEAD2 | KRT10 | RRM2 | IGHM | GSTP2 | ACSS3 | KRT1 | PCP4 | TYMS | CRB2</td></tr>
<tr><td>PROTEIN BINDING</td><td>GO:0005515</td><td>0.003842264</td><td>PCNA | PLP2 | TMEFF1 | DCC | C1QL1 | PRIM1 | SMARCC1 | MBTD1 | RFC5 | GPC3 | PYCR1 | LRRC58 | ZBTB45 | PTMA | HAUS7 | PANX1 | CRYM | CDKN2C | ZNHIT6 | KHDRBS3 | RRM1 | BCOR | SHH | L3MBTL3 | PTBP1 | MAPKAPK5 | MDK | RECK | AFAP1 | CDK4 | KLHL18 | FBXO25 | TLR3 | IL18 | CXADR | RBPJ | TMSB10 | ZBTB33 | ZNF423 | RFC2 | CD180 | H2AC11 | SEMA6A | TNS2 | HAT1 | KCTD16 | DCTPP1 | SPEG | TIMM22 | SELENOP | AVIL | H3C2 | KRT5 | MTF1 | PDZD11 | RHOQ | TEAD2 | KRT10 | RRM2 | IGHM | GSTP2 | KRT1 | PCP4 | TYMS | CRB2</td></tr>
<tr><td>SMALL MOLECULE BINDING</td><td>GO:0036094</td><td>0.021557448</td><td>RCN1 | ZIK1 | PRSS2 | CKMT2 | DCTPP1 | SPEG | SELENOP | ZNF667 | KLF3 | MCM5 | AVIL | PRIM1 | ARF3 | MTF1 | MBTD1 | RFC5 | RHOQ | OTOF | RRM2 | IGHM | ZBTB45 | ACSS3 | HINT2 | PCP4 | TYMS | CRYM | CRB2 | ZNHIT6 | RAB40C | RRM1 | COQ10B | HPDL | SHH | L3MBTL3 | MAPKAPK5 | MDK | CDK4 | MTARC1 | UBE2S | ZBTB33 | ZNF423 | RFC2 | GPN1 | MBD1 | TNS2 | ANTXR2</td></tr>
<tr><td>SYSTEM DEVELOPMENT</td><td>GO:0048731</td><td>0.024642318</td><td>RCN1 | PCNA | SPEG | SELENOP | AVIL | DCC | OTX2 | C1QL1 | SMARCC1 | MTF1 | MBTD1 | TEAD2 | GPC3 | ZBTB45 | PCP4 | TYMS | CRB2 | CDKN2C | MPZ | RRM1 | BCOR | SHH | PTBP1 | MAPKAPK5 | MDK | RECK | TLR3 | IL18 | CXADR | RBPJ | ZNF423 | SEMA6A | TNS2 | ANTXR2</td></tr>
<tr><td rowspan="2">Ribonucleotide Reductase (dNTP Synthesis)</td><td>RIBONUCLEOSIDE-DIPHOSPHATE REDUCTASE ACTIVITY, THIOREDOXIN DISULFIDE AS ACCEPTOR</td><td>GO:0004748</td><td>0.024944372</td><td>RRM2 | RRM1</td></tr>
<tr><td>RIBONUCLEOSIDE-DIPHOSPHATE REDUCTASE ACTIVITY</td><td>GO:0061731</td><td>0.024944372</td><td>RRM2 | RRM1</td></tr>
<tr><td rowspan="2">DNA Precursor Metabolism</td><td>PYRIMIDINE METABOLISM</td><td>KEGG:00240</td><td>0.022904851</td><td>RRM2 | DCTPP1 | TYMS | RRM1</td></tr>
<tr><td>2'-DEOXYRIBONUCLEOTIDE METABOLIC PROCESS</td><td>GO:0009394</td><td>0.047762517</td><td>RRM2 | DCTPP1 | TYMS | RRM1</td></tr>
<tr><td rowspan="2">Protein Complex Assembly</td><td>PROTEIN HETEROTETRAMERIZATION</td><td>GO:0051290</td><td>0.000574968</td><td>KRT10 | RRM2 | KRT1 | RRM1</td></tr>
<tr><td>PROTEIN HETEROOLIGOMERIZATION</td><td>GO:0051291</td><td>0.011031334</td><td>KRT10 | RRM2 | KRT1 | RRM1</td></tr>
<tr><td></td><td>POSITIVE REGULATION OF BMP SIGNALING PATHWAY</td><td>GO:0030513</td><td>0.02900287</td><td>GPC3 | RBPJ | ZNF423 | CRB2</td></tr>
</table>
