# Supplementary material for: Multi-omics characterization of radiation-induced cerebellar remodeling and tumorigenic transcriptional programs
Source: Neoplasia. 2026 Jun 29;79:101333. doi: 10.1016/j.neo.2026.101333 (PMC13330529; doi:10.1016/j.neo.2026.101333)
Supplement: Supplementary file 8 [file mmc8.pdf]

Common - Figure 6C

<table>
<tr><th>Cluster</th><th>DESCRIPTION</th><th>Name</th><th>FDR_qvalue</th><th>Genes</th></tr>
<tr><td rowspan="12">RNA Polymerase II-Mediated Transcriptional Regulation</td><td>RNA POLYMERASE II CIS-REGULATORY REGION SEQUENCE-SPECIFIC DNA BINDING</td><td>GO:0000978</td><td>0.011923568</td><td>DLX6|FEZF2|FOXD1|HOXA2|ZFP975</td></tr>
<tr><td>CIS-REGULATORY REGION SEQUENCE-SPECIFIC DNA BINDING</td><td>GO:0000987</td><td>0.013290346</td><td>DLX6|FEZF2|FOXD1|HOXA2|ZFP975</td></tr>
<tr><td>DNA-BINDING TRANSCRIPTION FACTOR ACTIVITY, RNA POLYMERASE II-SPECIFIC</td><td>GO:0000981</td><td>0.022741505</td><td>DLX6|FEZF2|FOXD1|HOXA2|ZFP975</td></tr>
<tr><td>RNA POLYMERASE II TRANSCRIPTION REGULATORY REGION SEQUENCE-SPECIFIC DNA BINDING</td><td>GO:0000977</td><td>0.026414374</td><td>DLX6|FEZF2|FOXD1|HOXA2|ZFP975</td></tr>
<tr><td>DNA-BINDING TRANSCRIPTION FACTOR ACTIVITY</td><td>GO:0003700</td><td>0.029884041</td><td>DLX6|FEZF2|FOXD1|HOXA2|ZFP975</td></tr>
<tr><td>REGIONALIZATION</td><td>GO:0003002</td><td>0.033575631</td><td>FEZF2|FOXD1|HOXA2|GDF3</td></tr>
<tr><td>TRANSCRIPTION CIS-REGULATORY REGION BINDING</td><td>GO:0000976</td><td>0.036983944</td><td>DLX6|FEZF2|FOXD1|HOXA2|ZFP975</td></tr>
<tr><td>TRANSCRIPTION REGULATORY REGION NUCLEIC ACID BINDING</td><td>GO:0001067</td><td>0.037618961</td><td>DLX6|FEZF2|FOXD1|HOXA2|ZFP975</td></tr>
<tr><td>REGULATION OF TRANSCRIPTION BY RNA POLYMERASE II</td><td>GO:0006357</td><td>0.042534043</td><td>DLX6|FEZF2|FOXD1|HOXA2|ZFP975|DLX6OS1|FRK</td></tr>
<tr><td>REGULATION OF RNA METABOLIC PROCESS</td><td>GO:0051252</td><td>0.042928859</td><td>DLX6|FEZF2|FOXD1|HOXA2|ZFP975|DLX6OS1|FRK|IGF2BP3</td></tr>
<tr><td>SEQUENCE-SPECIFIC DOUBLE-STRANDED DNA BINDING</td><td>GO:1990837</td><td>0.0465158</td><td>DLX6|FEZF2|FOXD1|HOXA2|ZFP975</td></tr>
<tr><td>PATTERN SPECIFICATION PROCESS</td><td>GO:0007389</td><td>0.049904768</td><td>FEZF2|FOXD1|HOXA2|GDF3</td></tr>
</table>

0.1Gy - Figure 6D

<table>
<tr><th>Cluster</th><th>DESCRIPTION</th><th>Name</th><th>FDR_qvalue</th><th>Genes</th></tr>
<tr><td rowspan="50">Innate Immunity and Autophagy Activation Module</td><td>IMMUNE RESPONSE</td><td>GO:0006955</td><td>4.38645E-25</td><td>H2/AA|H2/AB1|H2/M2|H2/Q4|H2/Q6|H2/Q7|IGHG3|IGHV1/39|IGHV1/9|IGHV14/2|IGHV3/6|IGHV8/8|IGHV9/3|IGLC2|TAP1|F830016B08RIK|GBP2B|GBP4|GBP8|GM4841|IGTP|IIGP1|IIGP1C|IRGM2|MX1|TGTP1|TGTP2|CD74|NLRC5|CFB|CXCL13|CXCL9|IGKV1/110|IGKV3/5|IGKV6/25|IGKV9/124|OASL1|PTGER4</td></tr>
<tr><td>IMMUNE SYSTEM PROCESS</td><td>GO:0002376</td><td>2.34288E-19</td><td>H2/AA|H2/AB1|H2/M2|H2/Q4|H2/Q6|H2/Q7|IGHG3|IGHV1/39|IGHV1/9|IGHV14/2|IGHV3/6|IGHV8/8|IGHV9/3|IGLC2|TAP1|F830016B08RIK|GBP2B|GBP4|GBP8|GM4841|IGTP|IIGP1|IIGP1C|IRGM2|MX1|TGTP1|TGTP2|CD74|NLRC5|CFB|CXCL13|CXCL9|IGKV1/110|IGKV3/5|IGKV6/25|IGKV9/124|OASL1|PTGER4</td></tr>
<tr><td>DEFENSE RESPONSE TO SYMBIONT</td><td>GO:0140546</td><td>3.38574E-14</td><td>CFB|H2/AA|H2/AB1|H2/Q7|CXCL13|IGHG3|CXCL9|OASL1|TAP1|F830016B08RIK|GBP2B|GBP4|GBP8|GM4841|IGTP|IIGP1|IIGP1C|IRGM2|MX1|TGTP1|TGTP2|CD74|NLRC5</td></tr>
<tr><td>DEFENSE RESPONSE TO OTHER ORGANISM</td><td>GO:0098542</td><td>1.70463E-12</td><td>CFB|H2/AA|H2/AB1|H2/Q7|CXCL13|IGHG3|CXCL9|OASL1|TAP1|F830016B08RIK|GBP2B|GBP4|GBP8|GM4841|IGTP|IIGP1|IIGP1C|IRGM2|MX1|TGTP1|TGTP2|CD74|NLRC5</td></tr>
<tr><td>RESPONSE TO TYPE II INTERFERON</td><td>GO:0034341</td><td>8.54793E-12</td><td>GBP2B|H2/AA|GBP4|H2/AB1|GBP8|H2/Q7|IGTP|TGTP1|TGTP2|CD74|NLRC5</td></tr>
<tr><td>INNATE IMMUNE RESPONSE</td><td>GO:0045087</td><td>2.38524E-11</td><td>CFB|H2/AA|H2/AB1|H2/Q7|OASL1|TAP1|F830016B08RIK|GBP2B|GBP4|GBP8|GM4841|IGTP|IIGP1|IIGP1C|IRGM2|MX1|TGTP1|TGTP2|CD74|NLRC5</td></tr>
<tr><td>RESPONSE TO OTHER ORGANISM</td><td>GO:0051707</td><td>2.19743E-10</td><td>CFB|H2/AA|H2/AB1|H2/Q7|CXCL13|IGHG3|CXCL9|OASL1|PTGER4|TAP1|F830016B08RIK|GBP2B|GBP4|GBP8|GM4841|IGTP|IIGP1|IIGP1C|IRGM2|MX1|TGTP1|TGTP2|CD74|NLRC5</td></tr>
<tr><td>RESPONSE TO EXTERNAL BIOTIC STIMULUS</td><td>GO:0043207</td><td>2.30983E-10</td><td>CFB|H2/AA|H2/AB1|H2/Q7|CXCL13|IGHG3|CXCL9|OASL1|PTGER4|TAP1|F830016B08RIK|GBP2B|GBP4|GBP8|GM4841|IGTP|IIGP1|IIGP1C|IRGM2|MX1|TGTP1|TGTP2|CD74|NLRC5</td></tr>
<tr><td>CELLULAR RESPONSE TO INTERFERON-BETA</td><td>GO:0035458</td><td>3.54839E-10</td><td>F830016B08RIK|GM4841|IGTP|IIGP1|IIGP1C|IRGM2|TGTP1|TGTP2</td></tr>
<tr><td>RESPONSE TO BIOTIC STIMULUS</td><td>GO:0009607</td><td>3.82364E-10</td><td>CFB|H2/AA|H2/AB1|H2/Q7|CXCL13|IGHG3|CXCL9|OASL1|PTGER4|TAP1|F830016B08RIK|GBP2B|GBP4|GBP8|GM4841|IGTP|IIGP1|IIGP1C|IRGM2|MX1|TGTP1|TGTP2|CD74|NLRC5</td></tr>
<tr><td>DEFENSE RESPONSE</td><td>GO:0006952</td><td>4.47407E-10</td><td>CFB|H2/AA|H2/AB1|H2/Q7|CXCL13|IGHG3|CXCL9|OASL1|PTGER4|TAP1|F830016B08RIK|GBP2B|GBP4|GBP8|GM4841|IGTP|IIGP1|IIGP1C|IRGM2|MX1|TGTP1|TGTP2|CD74|NLRC5</td></tr>
<tr><td>RESPONSE TO CYTOKINE</td><td>GO:0034097</td><td>8.64274E-10</td><td>H2/AA|H2/AB1|H2/Q7|CXCL13|CXCL9|OASL1|COL1A1|F830016B08RIK|GBP2B|GBP4|GBP8|GM4841|IGTP|IIGP1|IIGP1C|IRGM2|TGTP1|TGTP2|CD74|NLRC5</td></tr>
<tr><td>BIOLOGICAL PROCESS INVOLVED IN INTERSPECIES INTERACTION BETWEEN ORGANISMS</td><td>GO:0044419</td><td>1.02032E-09</td><td>CFB|H2/AA|H2/AB1|H2/Q7|CXCL13|IGHG3|CXCL9|OASL1|PTGER4|TAP1|F830016B08RIK|GBP2B|GBP4|GBP8|GM4841|IGTP|IIGP1|IIGP1C|IRGM2|MX1|TGTP1|TGTP2|CD74|NLRC5</td></tr>
<tr><td>RESPONSE TO PEPTIDE</td><td>GO:1901652</td><td>1.12762E-09</td><td>H2/AA|H2/AB1|H2/Q7|CXCL13|CXCL9|OASL1|COL1A1|F830016B08RIK|GBP2B|GBP4|GBP8|GM4841|IGTP|IIGP1|IIGP1C|IRGM2|TGTP1|TGTP2|CD74|NLRC5</td></tr>
<tr><td>RESPONSE TO INTERFERON-BETA</td><td>GO:0035456</td><td>1.24923E-09</td><td>F830016B08RIK|GM4841|IGTP|IIGP1|IIGP1C|IRGM2|TGTP1|TGTP2</td></tr>
<tr><td>CELLULAR RESPONSE TO CYTOKINE STIMULUS</td><td>GO:0071345</td><td>1.80715E-09</td><td>H2/AB1|H2/Q7|CXCL13|CXCL9|OASL1|COL1A1|F830016B08RIK|GBP2B|GBP4|GBP8|GM4841|IGTP|IIGP1|IIGP1C|IRGM2|TGTP1|TGTP2|CD74|NLRC5</td></tr>
<tr><td>DEFENSE RESPONSE TO PROTOZOAN</td><td>GO:0042832</td><td>2.26827E-09</td><td>GBP4|GBP8|IGTP|IIGP1|IRGM2|TGTP1|TGTP2</td></tr>
<tr><td>RESPONSE TO PROTOZOAN</td><td>GO:0001562</td><td>5.15182E-09</td><td>GBP4|GBP8|IGTP|IIGP1|IRGM2|TGTP1|TGTP2</td></tr>
<tr><td>RESPONSE TO EXTERNAL STIMULUS</td><td>GO:0009605</td><td>1.45054E-07</td><td>H2/AA|H2/AB1|H2/Q7|IGHG3|TAP1|F830016B08RIK|GBP2B|GBP4|GBP8|GM4841|IGTP|IIGP1|IIGP1C|IRGM2|MX1|TGTP1|TGTP2|CD74|NLRC5|CFB|CXCL13|CXCL9|OASL1|PTGER4|COL1A1</td></tr>
<tr><td>GTP BINDING</td><td>GO:0005525</td><td>2.8718E-07</td><td>F830016B08RIK|GBP2B|GBP4|GBP8|GM4841|GUCY2E|IGTP|IIGP1|IIGP1C|IRGM2|MX1|TGTP1|TGTP2</td></tr>
<tr><td>GUANYL NUCLEOTIDE BINDING</td><td>GO:0019001</td><td>6.13845E-07</td><td>F830016B08RIK|GBP2B|GBP4|GBP8|GM4841|GUCY2E|IGTP|IIGP1|IIGP1C|IRGM2|MX1|TGTP1|TGTP2</td></tr>
<tr><td>GUANYL RIBONUCLEOTIDE BINDING</td><td>GO:0032561</td><td>6.13845E-07</td><td>F830016B08RIK|GBP2B|GBP4|GBP8|GM4841|GUCY2E|IGTP|IIGP1|IIGP1C|IRGM2|MX1|TGTP1|TGTP2</td></tr>
<tr><td>AUTOPHAGOSOME ASSEMBLY</td><td>GO:0000045</td><td>7.26496E-07</td><td>F830016B08RIK|GM4841|IGTP|IIGP1|IIGP1C|IRGM2|TGTP1|TGTP2</td></tr>
<tr><td>AUTOPHAGOSOME ORGANIZATION</td><td>GO:1905037</td><td>1.11952E-06</td><td>F830016B08RIK|GM4841|IGTP|IIGP1|IIGP1C|IRGM2|TGTP1|TGTP2</td></tr>
<tr><td>CELLULAR RESPONSE TO TYPE II INTERFERON</td><td>GO:0071346</td><td>5.16576E-06</td><td>GBP2B|GBP4|H2/AB1|GBP8|H2/Q7|IGTP|NLRC5</td></tr>
<tr><td>RESPONSE TO STIMULUS</td><td>GO:0050896</td><td>3.12911E-05</td><td>H2/AA|H2/AB1|H2/M2|H2/Q4|H2/Q6|H2/Q7|IGHG3|IGHV1/39|IGHV1/9|IGHV14/2|IGHV3/6|IGHV8/8|IGHV9/3|IGLC2|TAP1|F830016B08RIK|GBP2B|GBP4|GBP8|GM4841|GUCY2E|IGTP|IIGP1|IIGP1C|IRGM2|MX1|TGTP1|TGTP2|CD74|NLRC5|P2RX6|CFB|KL|MYRF|CXCL13|CXCL9|IGKV1/110|IGKV3/5|IGKV6/25|IGKV9/124|OASL1|PTGER4|COL1A1|CACNA1H|HUS1B</td></tr>
<tr><td>RESPONSE TO STRESS</td><td>GO:0006950</td><td>3.45338E-05</td><td>H2/AA|H2/AB1|H2/Q7|IGHG3|TAP1|F830016B08RIK|GBP2B|GBP4|GBP8|GM4841|IGTP|IIGP1|IIGP1C|IRGM2|MX1|TGTP1|TGTP2|CD74|NLRC5|CFB|MYRF|CXCL13|CXCL9|OASL1|PTGER4|COL1A1|HUS1B</td></tr>
<tr><td>GTPASE ACTIVITY</td><td>GO:0003924</td><td>3.57202E-05</td><td>F830016B08RIK|GBP2B|GBP4|GBP8|GM4841|IGTP|IIGP1|IIGP1C|IRGM2|MX1|TGTP1|TGTP2</td></tr>
<tr><td>VACUOLE ORGANIZATION</td><td>GO:0007033</td><td>9.76978E-05</td><td>F830016B08RIK|GM4841|IGTP|IIGP1|IIGP1C|IRGM2|TGTP1|TGTP2</td></tr>
<tr><td>RIBONUCLEOSIDE TRIPHOSPHATE PHOSPHATASE ACTIVITY</td><td>GO:0017111</td><td>0.000477857</td><td>TAP1|F830016B08RIK|GBP2B|GBP4|GBP8|GM4841|IGTP|IIGP1|IIGP1C|IRGM2|MX1|TGTP1|TGTP2</td></tr>
<tr><td>MACROAUTOPHAGY</td><td>GO:0016236</td><td>0.000631153</td><td>F830016B08RIK|GM4841|IGTP|IIGP1|IIGP1C|IRGM2|TGTP1|TGTP2</td></tr>
<tr><td>PYROPHOSPHATASE ACTIVITY</td><td>GO:0016462</td><td>0.0008255</td><td>TAP1|F830016B08RIK|GBP2B|GBP4|GBP8|GM4841|IGTP|IIGP1|IIGP1C|IRGM2|MX1|TGTP1|TGTP2</td></tr>
<tr><td>HYDROLASE ACTIVITY, ACTING ON ACID ANHYDRIDES</td><td>GO:0016817</td><td>0.000833223</td><td>TAP1|F830016B08RIK|GBP2B|GBP4|GBP8|GM4841|IGTP|IIGP1|IIGP1C|IRGM2|MX1|TGTP1|TGTP2</td></tr>
<tr><td>HYDROLASE ACTIVITY, ACTING ON ACID ANHYDRIDES, IN PHOSPHORUS-CONTAINING ANHYDRIDES</td><td>GO:0016818</td><td>0.000833223</td><td>TAP1|F830016B08RIK|GBP2B|GBP4|GBP8|GM4841|IGTP|IIGP1|IIGP1C|IRGM2|MX1|TGTP1|TGTP2</td></tr>
<tr><td>RESPONSE TO BACTERIUM</td><td>GO:0009617</td><td>0.001419533</td><td>CFB|GBP4|GBP8|CXCL13|IGHG3|IIGP1|CXCL9|IRGM2|TGTP1|TGTP2|NLRC5|PTGER4</td></tr>
<tr><td>PURINE RIBONUCLEOSIDE TRIPHOSPHATE BINDING</td><td>GO:0035639</td><td>0.003780714</td><td>TAP1|F830016B08RIK|GBP2B|GBP4|GBP8|GM4841|GUCY2E|IGTP|IIGP1|IIGP1C|IRGM2|MX1|TGTP1|TGTP2|NLRC5|P2RX6</td></tr>
<tr><td>PURINE RIBONUCLEOTIDE BINDING</td><td>GO:0032555</td><td>0.005496613</td><td>TAP1|F830016B08RIK|GBP2B|GBP4|GBP8|GM4841|GUCY2E|IGTP|IIGP1|IIGP1C|IRGM2|MX1|TGTP1|TGTP2|NLRC5|P2RX6</td></tr>
<tr><td>HYDROLASE ACTIVITY</td><td>GO:0016787</td><td>0.005814099</td><td>CFB|ENPP6|KL|MYRF|PSMB9|PTPN7|SERPINA3G|TAP1|F830016B08RIK|GBP2B|GBP4|GBP8|GM4841|IGTP|IIGP1|IIGP1C|IRGM2|MX1|TGTP1|TGTP2</td></tr>
<tr><td>RIBONUCLEOTIDE BINDING</td><td>GO:0032553</td><td>0.00614729</td><td>TAP1|F830016B08RIK|GBP2B|GBP4|GBP8|GM4841|GUCY2E|IGTP|IIGP1|IIGP1C|IRGM2|MX1|TGTP1|TGTP2|NLRC5|P2RX6</td></tr>
<tr><td>CARBOHYDRATE DERIVATIVE BINDING</td><td>GO:0097367</td><td>0.008909173</td><td>CXCL13|TAP1|F830016B08RIK|GBP2B|GBP4|GBP8|GM4841|GUCY2E|IGTP|IIGP1|IIGP1C|IRGM2|MX1|TGTP1|TGTP2|NLRC5|P2RX6</td></tr>
<tr><td>PURINE NUCLEOTIDE BINDING</td><td>GO:0017076</td><td>0.010382424</td><td>TAP1|F830016B08RIK|GBP2B|GBP4|GBP8|GM4841|GUCY2E|IGTP|IIGP1|IIGP1C|IRGM2|MX1|TGTP1|TGTP2|NLRC5|P2RX6</td></tr>
<tr><td>NUCLEOTIDE BINDING</td><td>GO:0000166</td><td>0.024222772</td><td>TAP1|F830016B08RIK|GBP2B|GBP4|GBP8|GM4841|GUCY2E|IGTP|IIGP1|IIGP1C|IRGM2|MX1|TGTP1|TGTP2|NLRC5|P2RX6</td></tr>
<tr><td>NUCLEOSIDE PHOSPHATE BINDING</td><td>GO:1901265</td><td>0.027190599</td><td>TAP1|F830016B08RIK|GBP2B|GBP4|GBP8|GM4841|GUCY2E|IGTP|IIGP1|IIGP1C|IRGM2|MX1|TGTP1|TGTP2|NLRC5|P2RX6</td></tr>
<tr><td>PROCESS UTILIZING AUTOPHAGIC MECHANISM</td><td>GO:0061919</td><td>0.033920611</td><td>F830016B08RIK|GM4841|IGTP|IIGP1|IIGP1C|IRGM2|TGTP1|TGTP2</td></tr>
<tr><td>AUTOPHAGY</td><td>GO:0006914</td><td>0.033920611</td><td>F830016B08RIK|GM4841|IGTP|IIGP1|IIGP1C|IRGM2|TGTP1|TGTP2</td></tr>
<tr><td>RESPONSE TO VIRUS</td><td>GO:0009615</td><td>0.040135545</td><td>GBP4|CXCL9|MX1|TGTP1|TGTP2|OASL1|NLRC5</td></tr>
<tr><td></td><td>ANTIGEN PROCESSING AND PRESENTATION</td><td>KEGG:04612</td><td>6.24879E-09</td><td>H2/AA|H2/AB1|H2/M2|H2/Q4|H2/Q6|H2/Q7|CD74|TAP1</td></tr>
</table>

<table>
<tr><td>Antigen Processing and Cytotoxic T-Cell Response</td><td>ANTIGEN PROCESSING AND PRESENTATION OF PEPTIDE ANTIGEN</td><td>GO:0048002</td><td>1.32644E-08</td><td>H2/AA|H2/AB1|H2/M2|H2/Q4|H2/Q6|H2/Q7|CD74|TAP1</td></tr>
<tr><td></td><td>PEPTIDE ANTIGEN BINDING</td><td>GO:0042605</td><td>1.1101E-07</td><td>H2/AA|H2/AB1|H2/M2|H2/Q4|H2/Q6|H2/Q7|TAP1</td></tr>
<tr><td></td><td>ANTIGEN PROCESSING AND PRESENTATION</td><td>GO:0019882</td><td>5.25055E-07</td><td>H2/AA|H2/AB1|H2/M2|H2/Q4|H2/Q6|H2/Q7|CD74|TAP1</td></tr>
<tr><td></td><td>ALLOGRAFT REJECTION</td><td>KEGG:05330</td><td>9.13234E-07</td><td>H2/AA|H2/AB1|H2/M2|H2/Q4|H2/Q6|H2/Q7</td></tr>
<tr><td></td><td>GRAFT-VERSUS-HOST DISEASE</td><td>KEGG:05332</td><td>9.13234E-07</td><td>H2/AA|H2/AB1|H2/M2|H2/Q4|H2/Q6|H2/Q7</td></tr>
<tr><td></td><td>TYPE I DIABETES MELLITUS</td><td>KEGG:04940</td><td>1.90802E-06</td><td>H2/AA|H2/AB1|H2/M2|H2/Q4|H2/Q6|H2/Q7</td></tr>
<tr><td></td><td>AUTOIMMUNE THYROID DISEASE</td><td>KEGG:05320</td><td>4.36105E-06</td><td>H2/AA|H2/AB1|H2/M2|H2/Q4|H2/Q6|H2/Q7</td></tr>
<tr><td></td><td>ER-PHAGOSOME PATHWAY</td><td>REAC:R-MMU-1236974</td><td>7.72635E-06</td><td>H2/M2|H2/Q4|H2/Q6|H2/Q7|TAP1</td></tr>
<tr><td></td><td>VIRAL MYOCARDITIS</td><td>KEGG:05416</td><td>1.4863E-05</td><td>H2/AA|H2/AB1|H2/M2|H2/Q4|H2/Q6|H2/Q7</td></tr>
<tr><td></td><td>ANTIGEN PRESENTATION: FOLDING, ASSEMBLY AND PEPTIDE LOADING OF CLASS I MHC</td><td>REAC:R-MMU-983170</td><td>2.57509E-05</td><td>H2/M2|H2/Q4|H2/Q6|H2/Q7|TAP1</td></tr>
<tr><td></td><td>PHAGOSOME</td><td>KEGG:04145</td><td>4.96923E-05</td><td>H2/AA|H2/AB1|H2/M2|H2/Q4|H2/Q6|H2/Q7|TAP1</td></tr>
<tr><td></td><td>ANTIGEN PROCESSING AND PRESENTATION OF ENDOGENOUS PEPTIDE ANTIGEN VIA MHC CLASS I</td><td>GO:0019885</td><td>5.84697E-05</td><td>H2/M2|H2/Q4|H2/Q6|H2/Q7|TAP1</td></tr>
<tr><td></td><td>ANTIGEN PROCESSING AND PRESENTATION OF ENDOGENOUS PEPTIDE ANTIGEN</td><td>GO:0002483</td><td>6.62557E-05</td><td>H2/M2|H2/Q4|H2/Q6|H2/Q7|TAP1</td></tr>
<tr><td></td><td>ANTIGEN PROCESSING AND PRESENTATION OF ENDOGENOUS ANTIGEN</td><td>GO:0019883</td><td>7.48434E-05</td><td>H2/M2|H2/Q4|H2/Q6|H2/Q7|TAP1</td></tr>
<tr><td></td><td>PEPTIDE BINDING</td><td>GO:0042277</td><td>9.4451E-05</td><td>H2/AA|H2/AB1|H2/M2|H2/Q4|H2/Q6|H2/Q7|CD74|TAP1</td></tr>
<tr><td></td><td>ENDOSOMAL/VACUOLAR PATHWAY</td><td>REAC:R-MMU-1236977</td><td>0.000157164</td><td>H2/M2|H2/Q4|H2/Q6|H2/Q7</td></tr>
<tr><td></td><td>POSITIVE REGULATION OF IMMUNE RESPONSE</td><td>GO:0050778</td><td>0.000167043</td><td>CFB|H2/AA|H2/AB1|H2/M2|H2/Q4|H2/Q6|H2/Q7|IGHG3|IRGM2|CD74|OASL1|NLRC5</td></tr>
<tr><td></td><td>REGULATION OF IMMUNE RESPONSE</td><td>GO:0050776</td><td>0.000198281</td><td>CFB|H2/AA|H2/AB1|H2/M2|H2/Q4|H2/Q6|H2/Q7|IGHG3|OASL1|TAP1|IRGM2|CD74|NLRC5</td></tr>
<tr><td></td><td>ANTIGEN PROCESSING AND PRESENTATION OF PEPTIDE ANTIGEN VIA MHC CLASS I</td><td>GO:0002474</td><td>0.000219279</td><td>H2/M2|H2/Q4|H2/Q6|H2/Q7|TAP1</td></tr>
<tr><td></td><td>EPSTEIN-BARR VIRUS INFECTION</td><td>KEGG:05169</td><td>0.000272134</td><td>H2/AA|H2/AB1|H2/M2|H2/Q4|H2/Q6|H2/Q7|TAP1</td></tr>
<tr><td></td><td>HERPES SIMPLEX VIRUS 1 INFECTION</td><td>KEGG:05168</td><td>0.000338064</td><td>H2/AA|H2/AB1|ZFP677|H2/M2|H2/Q4|H2/Q6|H2/Q7|CD74|TAP1</td></tr>
<tr><td></td><td>HUMAN PAPILLOMAVIRUS INFECTION</td><td>KEGG:05165</td><td>0.000557708</td><td>H2/M2|MX2|H2/Q4|H2/Q6|H2/Q7|OASL1|PTGER4|COL1A1</td></tr>
<tr><td></td><td>ADAPTIVE IMMUNE SYSTEM</td><td>REAC:R-MMU-1280218</td><td>0.000591885</td><td>H2/M2|H2/Q4|H2/Q6|H2/Q7|IGKV1/110|IGHV3/6|IGHV8/8|CD74|IGLC2|COL1A1|TAP1</td></tr>
<tr><td></td><td>CELL KILLING</td><td>GO:0001906</td><td>0.000726843</td><td>H2/M2|H2/Q4|H2/Q6|H2/Q7|IGTP|IRGM2|TAP1</td></tr>
<tr><td></td><td>CELL ADHESION MOLECULES</td><td>KEGG:04514</td><td>0.000863977</td><td>H2/AA|H2/AB1|H2/M2|H2/Q4|H2/Q6|H2/Q7</td></tr>
<tr><td></td><td>ANTIGEN PROCESSING-CROSS PRESENTATION</td><td>REAC:R-MMU-1236975</td><td>0.001495954</td><td>H2/M2|H2/Q4|H2/Q6|H2/Q7|TAP1</td></tr>
<tr><td></td><td>ANTIGEN PROCESSING AND PRESENTATION OF ENDOGENOUS PEPTIDE ANTIGEN VIA MHC CLASS I VIA ER P</td><td>GO:0002486</td><td>0.002182343</td><td>H2/M2|H2/Q4|H2/Q6|H2/Q7</td></tr>
<tr><td></td><td>ANTIGEN PROCESSING AND PRESENTATION OF ENDOGENOUS PEPTIDE ANTIGEN VIA MHC CLASS I VIA ER P</td><td>GO:0002484</td><td>0.002442718</td><td>H2/M2|H2/Q4|H2/Q6|H2/Q7</td></tr>
<tr><td></td><td>ANTIGEN PROCESSING AND PRESENTATION OF ENDOGENOUS PEPTIDE ANTIGEN VIA MHC CLASS IB</td><td>GO:0002476</td><td>0.002442718</td><td>H2/M2|H2/Q4|H2/Q6|H2/Q7</td></tr>
<tr><td></td><td>POSITIVE REGULATION OF ADAPTIVE IMMUNE RESPONSE</td><td>GO:0002821</td><td>0.00260314</td><td>H2/AB1|H2/M2|H2/Q4|H2/Q6|H2/Q7|CD74</td></tr>
<tr><td></td><td>ANTIGEN PROCESSING AND PRESENTATION OF PEPTIDE ANTIGEN VIA MHC CLASS IB</td><td>GO:0002428</td><td>0.002725465</td><td>H2/M2|H2/Q4|H2/Q6|H2/Q7</td></tr>
<tr><td></td><td>ANTIGEN PROCESSING AND PRESENTATION VIA MHC CLASS IB</td><td>GO:0002475</td><td>0.003362947</td><td>H2/M2|H2/Q4|H2/Q6|H2/Q7</td></tr>
<tr><td></td><td>HUMAN T-CELL LEUKEMIA VIRUS 1 INFECTION</td><td>KEGG:05166</td><td>0.005542549</td><td>H2/AA|H2/AB1|H2/M2|H2/Q4|H2/Q6|H2/Q7</td></tr>
<tr><td></td><td>DAP12 INTERACTIONS</td><td>REAC:R-MMU-2172127</td><td>0.006086019</td><td>H2/M2|H2/Q4|H2/Q6|H2/Q7</td></tr>
<tr><td></td><td>HUMAN CYTOMEGALOVIRUS INFECTION</td><td>KEGG:05163</td><td>0.00634763</td><td>H2/M2|H2/Q4|H2/Q6|H2/Q7|PTGER4|TAP1</td></tr>
<tr><td></td><td>REGULATION OF LEUKOCYTE MEDIATED CYTOTOXICITY</td><td>GO:0001910</td><td>0.00809986</td><td>H2/M2|H2/Q4|H2/Q6|H2/Q7|TAP1</td></tr>
<tr><td></td><td>POSITIVE REGULATION OF T CELL MEDIATED CYTOTOXICITY</td><td>GO:0001916</td><td>0.00902566</td><td>H2/M2|H2/Q4|H2/Q6|H2/Q7</td></tr>
<tr><td></td><td>REGULATION OF T CELL MEDIATED CYTOTOXICITY</td><td>GO:0001914</td><td>0.015142476</td><td>H2/M2|H2/Q4|H2/Q6|H2/Q7</td></tr>
<tr><td></td><td>REGULATION OF CELL KILLING</td><td>GO:0031341</td><td>0.015815101</td><td>H2/M2|H2/Q4|H2/Q6|H2/Q7|TAP1</td></tr>
<tr><td></td><td>REGULATION OF ADAPTIVE IMMUNE RESPONSE</td><td>GO:0002819</td><td>0.024809293</td><td>H2/AB1|H2/M2|H2/Q4|H2/Q6|H2/Q7|CD74</td></tr>
<tr><td></td><td>T CELL MEDIATED CYTOTOXICITY</td><td>GO:0001913</td><td>0.032065285</td><td>H2/M2|H2/Q4|H2/Q6|H2/Q7</td></tr>
<tr><td></td><td>LEUKOCYTE MEDIATED CYTOTOXICITY</td><td>GO:0001909</td><td>0.036990966</td><td>H2/M2|H2/Q4|H2/Q6|H2/Q7|TAP1</td></tr>
<tr><td></td><td>POSITIVE REGULATION OF ADAPTIVE IMMUNE RESPONSE BASED ON SOMATIC RECOMBINATION OF IMMUNE</td><td>GO:0002824</td><td>0.039439982</td><td>H2/AB1|H2/M2|H2/Q4|H2/Q6|H2/Q7</td></tr>
<tr><td></td><td>HUMAN IMMUNODEFICIENCY VIRUS 1 INFECTION</td><td>KEGG:05170</td><td>0.044664625</td><td>H2/M2|H2/Q4|H2/Q6|H2/Q7|TAP1</td></tr>
<tr><td>Immune Signaling and Inflammatory Response</td><td>IMMUNOREGULATORY INTERACTIONS BETWEEN A LYMPHOID AND A NON-LYMPHOID CELL</td><td>REAC:R-MMU-198933</td><td>6.76465E-08</td><td>H2/M2|H2/Q4|H2/Q6|H2/Q7|IGKV1/110|IGHV3/6|IGHV8/8|IGLC2|COL1A1</td></tr>
<tr><td></td><td>INITIAL TRIGGERING OF COMPLEMENT</td><td>REAC:R-MMU-166663</td><td>1.37078E-05</td><td>CFB|IGHG3|IGKV1/110|IGHV3/6|IGHV8/8|IGLC2</td></tr>
<tr><td></td><td>REGULATION OF COMPLEMENT CASCADE</td><td>REAC:R-MMU-977606</td><td>5.4618E-05</td><td>CFB|IGHG3|IGKV1/110|IGHV3/6|IGHV8/8|IGLC2</td></tr>
<tr><td></td><td>COMPLEMENT CASCADE</td><td>REAC:R-MMU-166658</td><td>9.77818E-05</td><td>CFB|IGHG3|IGKV1/110|IGHV3/6|IGHV8/8|IGLC2</td></tr>
<tr><td></td><td>CLASSICAL ANTIBODY-MEDIATED COMPLEMENT ACTIVATION</td><td>REAC:R-MMU-173623</td><td>0.000180409</td><td>IGHG3|IGKV1/110|IGHV3/6|IGHV8/8|IGLC2</td></tr>
<tr><td></td><td>FCGR ACTIVATION</td><td>REAC:R-MMU-2029481</td><td>0.000275938</td><td>IGHG3|IGKV1/110|IGHV3/6|IGHV8/8|IGLC2</td></tr>
<tr><td></td><td>CREATION OF C4 AND C2 ACTIVATORS</td><td>REAC:R-MMU-166786</td><td>0.000299105</td><td>IGHG3|IGKV1/110|IGHV3/6|IGHV8/8|IGLC2</td></tr>
<tr><td></td><td>ROLE OF PHOSPHOLIPIDS IN PHAGOCYTOSIS</td><td>REAC:R-MMU-2029485</td><td>0.000507604</td><td>IGHG3|IGKV1/110|IGHV3/6|IGHV8/8|IGLC2</td></tr>
<tr><td></td><td>CELL SURFACE INTERACTIONS AT THE VASCULAR WALL</td><td>REAC:R-MMU-202733</td><td>0.001714789</td><td>IGKV1/110|IGHV3/6|IGHV8/8|CD74|IGLC2|COL1A1</td></tr>
<tr><td></td><td>REGULATION OF ACTIN DYNAMICS FOR PHAGOCYTIC CUP FORMATION</td><td>REAC:R-MMU-2029482</td><td>0.003268704</td><td>IGHG3|IGKV1/110|IGHV3/6|IGHV8/8|IGLC2</td></tr>
<tr><td></td><td>CD22 MEDIATED BCR REGULATION</td><td>REAC:R-MMU-5690714</td><td>0.005663084</td><td>IGKV1/110|IGHV3/6|IGHV8/8|IGLC2</td></tr>
<tr><td></td><td>FCGAMMA RECEPTOR (FCGR) DEPENDENT PHAGOCYTOSIS</td><td>REAC:R-MMU-2029480</td><td>0.00780358</td><td>IGHG3|IGKV1/110|IGHV3/6|IGHV8/8|IGLC2</td></tr>
<tr><td></td><td>ROLE OF LAT2/NTAL/LAB ON CALCIUM MOBILIZATION</td><td>REAC:R-MMU-2730905</td><td>0.008557603</td><td>IGKV1/110|IGHV3/6|IGHV8/8|IGLC2</td></tr>
<tr><td></td><td>INNATE IMMUNE SYSTEM</td><td>REAC:R-MMU-168249</td><td>0.013851978</td><td>CFB|H2/M2|H2/Q4|H2/Q6|H2/Q7|IGHG3|IGKV1/110|IGHV3/6|IGHV8/8|IGLC2|NLRC5</td></tr>
<tr><td></td><td>SCAVENGING OF HEME FROM PLASMA</td><td>REAC:R-MMU-2168880</td><td>0.014756453</td><td>IGKV1/110|IGHV3/6|IGHV8/8|IGLC2</td></tr>
<tr><td></td><td>ANTIGEN ACTIVATES B CELL RECEPTOR (BCR) LEADING TO GENERATION OF SECOND MESSENGERS</td><td>REAC:R-MMU-983695</td><td>0.015604902</td><td>IGKV1/110|IGHV3/6|IGHV8/8|IGLC2</td></tr>
<tr><td></td><td>FCERI MEDIATED CA+2 MOBILIZATION</td><td>REAC:R-MMU-2871809</td><td>0.017408022</td><td>IGKV1/110|IGHV3/6|IGHV8/8|IGLC2</td></tr>
<tr><td></td><td>FCERI MEDIATED MAPK ACTIVATION</td><td>REAC:R-MMU-2871796</td><td>0.020391709</td><td>IGKV1/110|IGHV3/6|IGHV8/8|IGLC2</td></tr>
<tr><td></td><td>BINDING AND UPTAKE OF LIGANDS BY SCAVENGER RECEPTORS</td><td>REAC:R-MMU-2173782</td><td>0.027454466</td><td>IGKV1/110|IGHV3/6|IGHV8/8|IGLC2</td></tr>
<tr><td>Antibody-Mediated Immunity</td><td>ANTIGEN BINDING</td><td>GO:0003823</td><td>1.71893E-16</td><td>H2/AA|H2/AB1|H2/M2|H2/Q4|H2/Q6|H2/Q7|IGHG3|IGHV1/39|IGHV1/9|IGHV14/2|IGHV3/6|IGHV8/8|IGHV9/3|IGLC2|TAP1</td></tr>
<tr><td></td><td>LYMPHOCYTE MEDIATED IMMUNITY</td><td>GO:0002449</td><td>2.40262E-12</td><td>CFB|H2/AB1|H2/M2|H2/Q4|H2/Q6|H2/Q7|IGHG3|IGHV1/39|IGHV1/9|IGHV14/2|IGHV3/6|IGHV8/8|IGHV9/3|IGLC2|TAP1|CD74</td></tr>
<tr><td></td><td>ADAPTIVE IMMUNE RESPONSE</td><td>GO:0002250</td><td>2.78287E-11</td><td>CFB|H2/AA|H2/AB1|H2/M2|H2/Q4|H2/Q6|H2/Q7|IGHG3|IGHV1/39|IGHV1/9|IGHV14/2|IGHV3/6|IGHV8/8|IGHV9/3|IGLC2|TAP1|CD74</td></tr>
<tr><td></td><td>LEUKOCYTE MEDIATED IMMUNITY</td><td>GO:0002443</td><td>6.27318E-11</td><td>CFB|H2/AB1|H2/M2|H2/Q4|H2/Q6|H2/Q7|IGHG3|IGHV1/39|IGHV1/9|IGHV14/2|IGHV3/6|IGHV8/8|IGHV9/3|IGLC2|TAP1|CD74</td></tr>
<tr><td></td><td>ADAPTIVE IMMUNE RESPONSE BASED ON SOMATIC RECOMBINATION OF IMMUNE RECEPTORS BUILT FROM I</td><td>GO:0002460</td><td>1.14117E-10</td><td>CFB|H2/AB1|H2/M2|H2/Q4|H2/Q6|H2/Q7|IGHG3|IGHV1/39|IGHV1/9|IGHV14/2|IGHV3/6|IGHV8/8|IGHV9/3|IGLC2|CD74</td></tr>
<tr><td></td><td>IMMUNE EFFECTOR PROCESS</td><td>GO:0002252</td><td>2.2804E-09</td><td>CFB|H2/AB1|H2/M2|H2/Q4|H2/Q6|H2/Q7|IGHG3|IGHV1/39|IGHV1/9|IGHV14/2|IGHV3/6|IGHV8/8|IGHV9/3|IGLC2|PTGER4|TAP1|CD74</td></tr>
<tr><td></td><td>IMMUNOGLOBULIN MEDIATED IMMUNE RESPONSE</td><td>GO:0016064</td><td>2.01437E-08</td><td>CFB|H2/AB1|IGHG3|IGHV1/39|IGHV1/9|IGHV14/2|IGHV3/6|IGHV8/8|CD74|IGHV9/3|IGLC2</td></tr>
<tr><td></td><td>B CELL MEDIATED IMMUNITY</td><td>GO:0019724</td><td>2.2851E-08</td><td>CFB|H2/AB1|IGHG3|IGHV1/39|IGHV1/9|IGHV14/2|IGHV3/6|IGHV8/8|CD74|IGHV9/3|IGLC2</td></tr>
<tr><td></td><td>POSITIVE REGULATION OF IMMUNE SYSTEM PROCESS</td><td>GO:0002684</td><td>0.000226038</td><td>CFB|H2/AA|H2/AB1|H2/M2|H2/Q4|H2/Q6|H2/Q7|CXCL13|IGHG3|OASL1|PTGER4|IRGM2|CD74|NLRC5</td></tr>
<tr><td></td><td>REGULATION OF IMMUNE SYSTEM PROCESS</td><td>GO:0002682</td><td>0.002959165</td><td>CFB|H2/AA|H2/AB1|H2/M2|H2/Q4|H2/Q6|H2/Q7|CXCL13|IGHG3|OASL1|PTGER4|TAP1|IRGM2|CD74|NLRC5</td></tr>
<tr><td></td><td>IMMUNE SYSTEM</td><td>REAC:R-MMU-168256</td><td>0.003604231</td><td>CFB|H2/M2|H2/Q4|H2/Q6|H2/Q7|IGHG3|IGKV1/110|IGHV3/6|IGHV8/8|OASL1|IGLC2|COL1A1|TAP1|CD74|NLRC5</td></tr>
<tr><td>MHC protein complex</td><td>MHC CLASS II PROTEIN COMPLEX BINDING</td><td>GO:0023026</td><td>0.000841396</td><td>H2/AA|H2/AB1|CD74</td></tr>
<tr><td></td><td>MHC PROTEIN COMPLEX BINDING</td><td>GO:0023023</td><td>0.002305477</td><td>H2/AA|H2/AB1|CD74</td></tr>
<tr><td></td><td>TOXOPLASMOSIS</td><td>KEGG:05145</td><td>0.022494203</td><td>H2/AA|H2/AB1|IGTP|IRGM2</td></tr>
<tr><td></td><td>ANTIGEN PROCESSING AND PRESENTATION OF EXOGENOUS PEPTIDE ANTIGEN VIA MHC CLASS II</td><td>GO:0019886</td><td>0.023374217</td><td>H2/AA|H2/AB1|CD74</td></tr>
<tr><td></td><td>ANTIGEN PROCESSING AND PRESENTATION OF PEPTIDE ANTIGEN VIA MHC CLASS II</td><td>GO:0002495</td><td>0.040160459</td><td>H2/AA|H2/AB1|CD74</td></tr>
<tr><td></td><td>TYPE II INTERFERON SIGNALING IFNG</td><td>WP:WP1253</td><td>3.50151E-05</td><td>GBP2B|PSMB9|CXCL9|TAP1</td></tr>
<tr><td></td><td>CXCR3 CHEMOKINE RECEPTOR BINDING</td><td>GO:0048248</td><td>0.014270768</td><td>CXCL13|CXCL9</td></tr>
</table>

2Gy - Figure 6E

<table>
<tr><th>Cluster</th><th>DESCRIPTION</th><th>Name</th><th>FDR_qvalue</th><th>Genes</th></tr>
<tr><td></td><td>RNA PROCESSING</td><td>GO:0006396</td><td>6.90874E-33</td><td>GM22220|GM22739|GM22973|GM23330|GM23511|GM23849|GM23928|GM23971|GM24305|GM24497|GM24950|GM25360|GM26316|RBM24|RNU2-10|GM22505|GM24265|GM24407|CDKN2A|GM22422|GM22442|GM22620|GM22767|GM22806|GM23143|GM23262|GM23297|GM23442|GM23639|GM23751|GM24119|GM24201|GM24289|GM24451|GM24616|GM24698|GM25394|GM25636|GM25776|GM25777|GM25788|GM25835|GM25852|GM26165|GM26175|GM26397|GM26447|RPRL3|SNORA15|SNORA16A|SNORA17|SNORA21|SNORA23|SNORA24|SNORA26|SNORA3|SNORA43|SNORA44|SNORA52|SNORA64|SNORA68|SNORA70|SNORA81|SNORA9|SNORD118|SNORD15A|SNORD15B|SNORD16A|SNORD22|SNORD3A|SNORD94</td></tr>
</table>

RNA Metabolism and Gene Expression	RNA BIOSYNTHETIC PROCESS	GO:0032774	2.75679E-20	GM22220|GM22739|GM22973|GM23330|GM23511|GM23849|GM23928|GM23971|GM24305|GM24497|GM24950|GM25360|GM26316|RBM24|RNU2-10|GM22505|GM24265|GM24407|CDKN2A|GM22422|GM22442|GM22620|GM22767|GM22806|GM23143|GM23262|GM23297|GM23442|GM23639|GM23751|GM24119|GM24201|GM24289|GM24451|GM24616|GM24698|GM25394|GM25636|GM25776|GM25777|GM25788|GM25835|GM25852|GM26165|GM26175|GM26397|GM26447|RPRL3|SNORA15|SNORA16A|SNORA17|SNORA21|SNORA23|SNORA24|SNORA26|SNORA3|SNORA43|SNORA44|SNORA52|SNORA64|SNORA68|SNORA70|SNORA81|SNORA9|SNORD118|SNORD15A|SNORD15B|SNORD16A|SNORD22|SNORD3A|SNORD94|ELF3|LMO7|LUM|NEUROD4|PITX2|PLAGL1
	NUCLEIC ACID BIOSYNTHETIC PROCESS	GO:0141187	7.77357E-20	GM22220|GM22739|GM22973|GM23330|GM23511|GM23849|GM23928|GM23971|GM24305|GM24497|GM24950|GM25360|GM26316|RBM24|RNU2-10|GM22505|GM24265|GM24407|CDKN2A|GM22422|GM22442|GM22620|GM22767|GM22806|GM23143|GM23262|GM23297|GM23442|GM23639|GM23751|GM24119|GM24201|GM24289|GM24451|GM24616|GM24698|GM25394|GM25636|GM25776|GM25777|GM25788|GM25835|GM25852|GM26165|GM26175|GM26397|GM26447|RPRL3|SNORA15|SNORA16A|SNORA17|SNORA21|SNORA23|SNORA24|SNORA26|SNORA3|SNORA43|SNORA44|SNORA52|SNORA64|SNORA68|SNORA70|SNORA81|SNORA9|SNORD118|SNORD15A|SNORD15B|SNORD16A|SNORD22|SNORD3A|SNORD94|ELF3|LMO7|LUM|NEUROD4|PITX2|PLAGL1
	RNA METABOLIC PROCESS	GO:0016070	1.88234E-19	GM22220|GM22739|GM22973|GM23330|GM23511|GM23849|GM23928|GM23971|GM24305|GM24497|GM24950|GM25360|GM26316|RBM24|RNU2-10|GM22505|GM24265|GM24407|CDKN2A|GM22422|GM22442|GM22620|GM22767|GM22806|GM23143|GM23262|GM23297|GM23442|GM23639|GM23751|GM24119|GM24201|GM24289|GM24451|GM24616|GM24698|GM25394|GM25636|GM25776|GM25777|GM25788|GM25835|GM25852|GM26165|GM26175|GM26397|GM26447|RPRL3|SNORA15|SNORA16A|SNORA17|SNORA21|SNORA23|SNORA24|SNORA26|SNORA3|SNORA43|SNORA44|SNORA52|SNORA64|SNORA68|SNORA70|SNORA81|SNORA9|SNORD118|SNORD15A|SNORD15B|SNORD16A|SNORD22|SNORD3A|SNORD94|ELF3|LMO7|LUM|NEUROD4|PITX2|PLAGL1
	NUCLEOBASE-CONTAINING COMPOUND BIOSYNTHETIC PROCESS	GO:0034654	1.0988E-18	GM22220|GM22739|GM22973|GM23330|GM23511|GM23849|GM23928|GM23971|GM24305|GM24497|GM24950|GM25360|GM26316|RBM24|RNU2-10|GM22505|GM24265|GM24407|CDKN2A|GM22422|GM22442|GM22620|GM22767|GM22806|GM23143|GM23262|GM23297|GM23442|GM23639|GM23751|GM24119|GM24201|GM24289|GM24451|GM24616|GM24698|GM25394|GM25636|GM25776|GM25777|GM25788|GM25835|GM25852|GM26165|GM26175|GM26397|GM26447|RPRL3|SNORA15|SNORA16A|SNORA17|SNORA21|SNORA23|SNORA24|SNORA26|SNORA3|SNORA43|SNORA44|SNORA52|SNORA64|SNORA68|SNORA70|SNORA81|SNORA9|SNORD118|SNORD15A|SNORD15B|SNORD16A|SNORD22|SNORD3A|SNORD94|ELF3|LMO7|LUM|NEUROD4|PITX2|PLAGL1
	GENE EXPRESSION	GO:0010467	1.14086E-17	GM22220|GM22739|GM22973|GM23330|GM23511|GM23849|GM23928|GM23971|GM24305|GM24497|GM24950|GM25360|GM26316|RBM24|RNU2-10|GM22505|GM24265|GM24407|MT-TV|CDKN2A|GM22422|GM22442|GM22620|GM22767|GM22806|GM23143|GM23262|GM23297|GM23442|GM23639|GM23751|GM24119|GM24201|GM24289|GM24451|GM24616|GM24698|GM25394|GM25636|GM25776|GM25777|GM25788|GM25835|GM25852|GM26165|GM26175|GM26397|GM26447|RPRL3|SNORA15|SNORA16A|SNORA17|SNORA21|SNORA23|SNORA24|SNORA26|SNORA3|SNORA43|SNORA44|SNORA52|SNORA64|SNORA68|SNORA70|SNORA81|SNORA9|SNORD118|SNORD15A|SNORD15B|SNORD16A|SNORD22|SNORD3A|SNORD94|ELF3|LMO7|LUM|NEUROD4|PITX2|PLAGL1|ACTC1|CD5L|CXCL5|ERO1B|MGAT4C|PCSK9|SPON2
	NUCLEIC ACID METABOLIC PROCESS	GO:0090304	3.09817E-17	GM22220|GM22739|GM22973|GM23330|GM23511|GM23849|GM23928|GM23971|GM24305|GM24497|GM24950|GM25360|GM26316|RBM24|RNU2-10|GM22505|GM24265|GM24407|CDKN2A|GM22422|GM22442|GM22620|GM22767|GM22806|GM23143|GM23262|GM23297|GM23442|GM23639|GM23751|GM24119|GM24201|GM24289|GM24451|GM24616|GM24698|GM25394|GM25636|GM25776|GM25777|GM25788|GM25835|GM25852|GM26165|GM26175|GM26397|GM26447|RPRL3|SNORA15|SNORA16A|SNORA17|SNORA21|SNORA23|SNORA24|SNORA26|SNORA3|SNORA43|SNORA44|SNORA52|SNORA64|SNORA68|SNORA70|SNORA81|SNORA9|SNORD118|SNORD15A|SNORD15B|SNORD16A|SNORD22|SNORD3A|SNORD94|ELF3|LMO7|LUM|NEUROD4|PITX2|PLAGL1
	MACROMOLECULE BIOSYNTHETIC PROCESS	GO:0009059	2.78907E-16	GM22220|GM22739|GM22973|GM23330|GM23511|GM23849|GM23928|GM23971|GM24305|GM24497|GM24950|GM25360|GM26316|RBM24|RNU2-10|GM22505|GM24265|GM24407|MT-TV|CDKN2A|GM22422|GM22442|GM22620|GM22767|GM22806|GM23143|GM23262|GM23297|GM23442|GM23639|GM23751|GM24119|GM24201|GM24289|GM24451|GM24616|GM24698|GM25394|GM25636|GM25776|GM25777|GM25788|GM25835|GM25852|GM26165|GM26175|GM26397|GM26447|RPRL3|SNORA15|SNORA16A|SNORA17|SNORA21|SNORA23|SNORA24|SNORA26|SNORA3|SNORA43|SNORA44|SNORA52|SNORA64|SNORA68|SNORA70|SNORA81|SNORA9|SNORD118|SNORD15A|SNORD15B|SNORD16A|SNORD22|SNORD3A|SNORD94|ELF3|LMO7|LUM|NEUROD4|PITX2|PLAGL1|ACTC1|CD5L|CXCL5|ERO1B|MGAT4C|PCSK9|SPON2
	MACROMOLECULE METABOLIC PROCESS	GO:0043170	3.35383E-15	GM22220|GM22739|GM22973|GM23330|GM23511|GM23849|GM23928|GM23971|GM24305|GM24497|GM24950|GM25360|GM26316|RBM24|RNU2-10|GM22505|GM24265|GM24407|MT-TV|CDKN2A|GM22422|GM22442|GM22620|GM22767|GM22806|GM23143|GM23262|GM23297|GM23442|GM23639|GM23751|GM24119|GM24201|GM24289|GM24451|GM24616|GM24698|GM25394|GM25636|GM25776|GM25777|GM25788|GM25835|GM25852|GM26165|GM26175|GM26397|GM26447|RPRL3|SNORA15|SNORA16A|SNORA17|SNORA21|SNORA23|SNORA24|SNORA26|SNORA3|SNORA43|SNORA44|SNORA52|SNORA64|SNORA68|SNORA70|SNORA81|SNORA9|SNORD118|SNORD15A|SNORD15B|SNORD16A|SNORD22|SNORD3A|SNORD94|ELF3|LMO7|LUM|NEUROD4|PITX2|PLAGL1|ACTC1|CD5L|CXCL5|ERO1B|MGAT4C|PCSK9|SPON2|ADAMTS13|ADGRV1|CAPN12|MMP3|MMP9|PRTN3|ROR2
	NUCLEOBASE-CONTAINING COMPOUND METABOLIC PROCESS	GO:0006139	3.41158E-15	GM22220|GM22739|GM22973|GM23330|GM23511|GM23849|GM23928|GM23971|GM24305|GM24497|GM24950|GM25360|GM26316|RBM24|RNU2-10|GM22505|GM24265|GM24407|CDKN2A|GM22422|GM22442|GM22620|GM22767|GM22806|GM23143|GM23262|GM23297|GM23442|GM23639|GM23751|GM24119|GM24201|GM24289|GM24451|GM24616|GM24698|GM25394|GM25636|GM25776|GM25777|GM25788|GM25835|GM25852|GM26165|GM26175|GM26397|GM26447|RPRL3|SNORA15|SNORA16A|SNORA17|SNORA21|SNORA23|SNORA24|SNORA26|SNORA3|SNORA43|SNORA44|SNORA52|SNORA64|SNORA68|SNORA70|SNORA81|SNORA9|SNORD118|SNORD15A|SNORD15B|SNORD16A|SNORD22|SNORD3A|SNORD94|ELF3|LMO7|LUM|NEUROD4|PITX2|PLAGL1
	BIOSYNTHETIC PROCESS	GO:0009058	7.94063E-15	GM22220|GM22739|GM22973|GM23330|GM23511|GM23849|GM23928|GM23971|GM24305|GM24497|GM24950|GM25360|GM26316|RBM24|RNU2-10|GM22505|GM24265|GM24407|MT-TV|CDKN2A|GM22422|GM22442|GM22620|GM22767|GM22806|GM23143|GM23262|GM23297|GM23442|GM23639|GM23751|GM24119|GM24201|GM24289|GM24451|GM24616|GM24698|GM25394|GM25636|GM25776|GM25777|GM25788|GM25835|GM25852|GM26165|GM26175|GM26397|GM26447|RPRL3|SNORA15|SNORA16A|SNORA17|SNORA21|SNORA23|SNORA24|SNORA26|SNORA3|SNORA43|SNORA44|SNORA52|SNORA64|SNORA68|SNORA70|SNORA81|SNORA9|SNORD118|SNORD15A|SNORD15B|SNORD16A|SNORD22|SNORD3A|SNORD94|ELF3|LMO7|LUM|NEUROD4|PITX2|PLAGL1|ACTC1|CD5L|CXCL5|ERO1B|MGAT4C|PCSK9|SPON2|HMGCLL1|TPH2

<table>
<tr><th></th><th></th><th></th><th></th><th></th></tr>
<tr><td rowspan="3"></td><td>PRIMARY METABOLIC PROCESS</td><td>GO:0044238</td><td>4.82497E-14</td><td>GM22220|GM22739|GM22973|GM23330|GM23511|GM23849|GM23928|GM23971|GM24305|GM24497|GM24950|GM25360|GM26316|RBM24|RNU2-10|GM22505|GM24265|GM24407|MT-TV|CDKN2A|GM22422|GM22442|GM22620|GM22767|GM22806|GM23143|GM23262|GM23297|GM23442|GM23639|GM23751|GM24119|GM24201|GM24289|GM24451|GM24616|GM24698|GM25394|GM25636|GM25776|GM25777|GM25788|GM25835|GM25852|GM26165|GM26175|GM26397|GM26447|RPRL3|SNORA15|SNORA16A|SNORA17|SNORA21|SNORA23|SNORA24|SNORA26|SNORA3|SNORA43|SNORA44|SNORA52|SNORA64|SNORA68|SNORA70|SNORA81|SNORA9|SNORD118|SNORD15A|SNORD15B|SNORD16A|SNORD22|SNORD3A|SNORD94|ELF3|LMO7|LUM|NEUROD4|PITX2|PLAGL1|CD5L|ERO1B|MGAT4C|PCSK9|ADAMTS13|ADGRV1|CAPN12|MMP3|MMP9|PRTN3|ROR2|HMGCLL1|TPH2|ASPG</td></tr>
<tr><td>METABOLIC PROCESS</td><td>GO:0008152</td><td>3.39193E-12</td><td>GM22220|GM22739|GM22973|GM23330|GM23511|GM23849|GM23928|GM23971|GM24305|GM24497|GM24950|GM25360|GM26316|RBM24|RNU2-10|GM22505|GM24265|GM24407|MT-TV|CDKN2A|GM22422|GM22442|GM22620|GM22767|GM22806|GM23143|GM23262|GM23297|GM23442|GM23639|GM23751|GM24119|GM24201|GM24289|GM24451|GM24616|GM24698|GM25394|GM25636|GM25776|GM25777|GM25788|GM25835|GM25852|GM26165|GM26175|GM26397|GM26447|RPRL3|SNORA15|SNORA16A|SNORA17|SNORA21|SNORA23|SNORA24|SNORA26|SNORA3|SNORA43|SNORA44|SNORA52|SNORA64|SNORA68|SNORA70|SNORA81|SNORA9|SNORD118|SNORD15A|SNORD15B|SNORD16A|SNORD22|SNORD3A|SNORD94|ELF3|LMO7|LUM|NEUROD4|PITX2|PLAGL1|ACTC1|CD5L|CXCL5|ERO1B|MGAT4C|PCSK9|SPON2|ADAMTS13|ADGRV1|CAPN12|MMP3|MMP9|PRTN3|ROR2|HMGCLL1|TPH2|ASPG</td></tr>
<tr><td>CELLULAR PROCESS</td><td>GO:0009987</td><td>0.010976378</td><td>GM22220|GM22739|GM22973|GM23330|GM23511|GM23849|GM23928|GM23971|GM24305|GM24497|GM24950|GM25360|GM26316|RBM24|RNU2-10|GM22505|GM24265|GM24407|MT-TV|CDKN2A|GM22422|GM22442|GM22620|GM22767|GM22806|GM23143|GM23262|GM23297|GM23442|GM23639|GM23751|GM24119|GM24201|GM24289|GM24451|GM24616|GM24698|GM25394|GM25636|GM25776|GM25777|GM25788|GM25835|GM25852|GM26165|GM26175|GM26397|GM26447|RPRL3|SNORA15|SNORA16A|SNORA17|SNORA21|SNORA23|SNORA24|SNORA26|SNORA3|SNORA43|SNORA44|SNORA52|SNORA64|SNORA68|SNORA70|SNORA81|SNORA9|SNORD118|SNORD15A|SNORD15B|SNORD16A|SNORD22|SNORD3A|SNORD94|ELF3|LMO7|LUM|NEUROD4|PITX2|PLAGL1|ACTC1|CD5L|CXCL5|ERO1B|MGAT4C|PCSK9|SPON2|ADAMTS13|ADGRV1|CAPN12|MMP3|MMP9|PRTN3|ROR2|HMGCLL1|TPH2|ASPG|CRB2|GM20458|H3F4|ISLR2|MEI1|OLFML2B|STEAP4</td></tr>
<tr><td rowspan="14">Spliceosome and Pre-mRNA Splicing</td><td>PRE-MRNA BINDING</td><td>GO:0036002</td><td>7.86198E-12</td><td>GM22220|GM22739|GM22973|GM23330|GM23511|GM23849|GM23928|GM23971|GM24305|GM24497|GM24950|GM25360|GM26316|RBM24|RNU2-10</td></tr>
<tr><td>PRE-MRNA BRANCH POINT BINDING</td><td>GO:0045131</td><td>3.40671E-09</td><td>GM22973|GM23849|GM23971|GM24497|GM24950|GM25360|GM26316|RNU2-10</td></tr>
<tr><td>SPLICEOSOMAL COMPLEX ASSEMBLY</td><td>GO:0000245</td><td>8.19269E-08</td><td>GM22220|GM22739|GM22973|GM23330|GM23511|GM23849|GM23928|GM23971|GM24305|GM24497|GM24950|GM25360|GM26316|RNU2-10</td></tr>
<tr><td>MRNA BRANCH SITE RECOGNITION</td><td>GO:0000348</td><td>1.25503E-07</td><td>GM22973|GM23849|GM23971|GM24497|GM24950|GM25360|GM26316|RNU2-10</td></tr>
<tr><td>SPLICEOSOME</td><td>KEGG:03040</td><td>3.05279E-06</td><td>GM22505|GM24265|GM24407|GM22973|GM23143|GM25360|GM26316|RNU2-10</td></tr>
<tr><td>RNA SPLICING, VIA TRANSESTERIFICATION REACTIONS</td><td>GO:0000375</td><td>0.010832189</td><td>GM22220|GM22739|GM22973|GM23330|GM23511|GM23849|GM23928|GM23971|GM24305|GM24497|GM24950|GM25360|GM26316|RBM24|RNU2-10|GM22505|GM24265|GM24407|GM23143</td></tr>
<tr><td>MRNA SPLICING, VIA SPLICEOSOME</td><td>GO:0000398</td><td>0.010832189</td><td>GM22220|GM22739|GM22973|GM23330|GM23511|GM23849|GM23928|GM23971|GM24305|GM24497|GM24950|GM25360|GM26316|RBM24|RNU2-10|GM22505|GM24265|GM24407|GM23143</td></tr>
<tr><td>RNA SPLICING, VIA TRANSESTERIFICATION REACTIONS WITH BULGED ADENOSINE AS NUCLEOPHILE</td><td>GO:0000377</td><td>0.010832189</td><td>GM22220|GM22739|GM22973|GM23330|GM23511|GM23849|GM23928|GM23971|GM24305|GM24497|GM24950|GM25360|GM26316|RBM24|RNU2-10|GM22505|GM24265|GM24407|GM23143</td></tr>
<tr><td>PROTEIN-RNA COMPLEX ASSEMBLY</td><td>GO:0022618</td><td>0.012230382</td><td>GM22220|GM22739|GM22973|GM23330|GM23511|GM23849|GM23928|GM23971|GM24305|GM24497|GM24950|GM25360|GM26316|RNU2-10|GM22505|GM24265|GM24407|GM23143</td></tr>
<tr><td>PROTEIN-RNA COMPLEX ORGANIZATION</td><td>GO:0071826</td><td>0.013428153</td><td>GM22220|GM22739|GM22973|GM23330|GM23511|GM23849|GM23928|GM23971|GM24305|GM24497|GM24950|GM25360|GM26316|RNU2-10|GM22505|GM24265|GM24407|GM23143</td></tr>
<tr><td>PRE-MRNA 5'-SPLICE SITE BINDING</td><td>GO:0030627</td><td>0.025185495</td><td>GM22220|GM22739|GM23330|GM23511|GM23928|GM24305</td></tr>
<tr><td>RNA BINDING</td><td>GO:0003723</td><td>0.033849064</td><td>GM22220|GM22739|GM22973|GM23330|GM23511|GM23849|GM23928|GM23971|GM24305|GM24497|GM24950|GM25360|GM26316|RBM24|RNU2-10|GM22505|GM24265|GM24407|MT-TV</td></tr>
<tr><td>RIBONUCLEOPROTEIN COMPLEX BIOGENESIS</td><td>GO:0022613</td><td>0.037681664</td><td>GM22220|GM22739|GM22973|GM23330|GM23511|GM23849|GM23928|GM23971|GM24305|GM24497|GM24950|GM25360|GM26316|RNU2-10|GM22505|GM24265|GM24407|CDKN2A|GM23143</td></tr>
<tr><td>RNA SPLICING</td><td>GO:0008380</td><td>0.043730639</td><td>GM22220|GM22739|GM22973|GM23330|GM23511|GM23849|GM23928|GM23971|GM24305|GM24497|GM24950|GM25360|GM26316|RBM24|RNU2-10|GM22505|GM24265|GM24407|GM23143</td></tr>
</table>
