# Supplementary material for: Multi-omics characterization of radiation-induced cerebellar remodeling and tumorigenic transcriptional programs
Source: Neoplasia. 2026 Jun 29;79:101333. doi: 10.1016/j.neo.2026.101333 (PMC13330529; doi:10.1016/j.neo.2026.101333)
Supplement: Supplementary file 9 [file mmc9.docx]

**Supplementary Fig. S1.** **Modulated proteins in the cerebellar proteome of irradiated mice after 1 week.** Proteins **(A)** commonly modulated between 0.1 Gy and 2 Gy, or specifically modulated by **(B)** 0.1 Gy, **(C)** 2 Gy are shown as protein-protein interaction networks generated using the STRING database. Node fill colors represent the direction and magnitude of regulation (red: upregulated; blue: downregulated) relative to SHAM controls. Border thickness indicates statistical significance (thinner borders = lower p-values; range: p = 0 to 0.05).

**Fig. S2. Modulated proteins in the cerebellar proteome of irradiated mice after 6 weeks.** Proteins **(A)** commonly modulated between 0.1 Gy and 2 Gy, or specifically modulated by **(B)** 0.1 Gy, **(C)** 2 Gy are shown as protein-protein interaction networks generated using the STRING database. Node fill colors represent the direction and magnitude of regulation (red: upregulated; blue: downregulated) relative to SHAM controls. Border thickness indicates statistical significance (thinner borders = lower p-values; range: p = 0 to 0.05).

**Fig. S3. Early transcriptional responses of the cerebellum 1 week after γ-irradiation**.
Whole-cerebellum gene expression analysis was performed in *Ptch1^+/−^* mice 1 week after exposure to 0.1 Gy or 2 Gy γ-irradiation. (A) mRNA expression of granule cell–specific markers (Math1, Zic, NeuN) and markers of other cerebellar cell types, including immature neurons (βIII-tubulin), Purkinje cells (Calbindin-1), glial cells (GFAP), and Bergmann glia (S100β).(B) mRNA expression of pro-inflammatory cytokines (TNF-α, IL-6), the anti-inflammatory cytokine TGF-β, components of the Sonic Hedgehog (SHH) signaling pathway (Gli1), the chemokine CXCL5, and angiogenic markers (CD31, VEGF).Data are presented as mean ± SEM (N = 8 animals). Statistical significance was determined using one-way ANOVA followed by Tukey’s multiple comparisons test; *p < 0.05.

**Fig. S4. Modulated genes in MB transcriptome. (A)** genes commonly modulated between 0.1Gy and 2Gy, or genes specifically modulated in MB induced in **(B)** 0.1 Gy irradiated mice, **(C)** 2 Gy irradiated mice are shown as RNA–RNA interaction network based on the NPInter database (Black edges) and as Protein-Protein interaction based on the STRING database (Red edges). Node fill color represents direction and magnitude of expression change compared with SHAM controls (red = upregulated; blue = downregulated). Border thickness reflects statistical significance (thinner borders = lower p-values, range p = 0–0.05). Node shapes indicate RNA class: diamonds = snRNA/snoRNA, octagons = lncRNA, triangles = miRNA, and ellipses = mRNA.
