# Supplementary material for: Multi-omics characterization of radiation-induced cerebellar remodeling and tumorigenic transcriptional programs
Source: Neoplasia. 2026 Jun 29;79:101333. doi: 10.1016/j.neo.2026.101333 (PMC13330529; doi:10.1016/j.neo.2026.101333)
Supplement: Supplementary file 10 [file mmc10.pdf]

Supplementary Table 1: Methylome

Deregulated DNA regions 1 week 0.1Gy vs Sham

Chromosome	Start	End	P-Value	Q-Value	Difference	Nearest Transcript ID	Nearest Gene Name	Distance to Gene
chr1	152730001	152731000	1.3E-136	2.0E-134	0.589908476953168	XM_006529266.4	Rgl1	0
chr1	184793001	184794000	5.3E-129	7.3E-127	0.417577690956665	NM_001290273.1	Mtarc1	0
chr7	63803001	63804000	1.3E-76	1.2E-74	0.240986148185304	XR_882151.2	Gm39023	19238
chr14	63193001	63194000	1.5E-32	1.1E-30	0.241465058181579	NM_201610.2	Neil2	0
chr4	137723001	137724000	3.3E-31	2.3E-29	-0.208328561940316	NM_001331215.1	Rap1gap	0

Deregulated DNA regions 1 week 2Gy vs Sham

Chromosome	Start	End	P-Value	Q-Value	Difference	Nearest Transcript ID	Nearest Gene Name	Distance to Gene
chr13	119488001	119489000	0	0	0.246776070717265	NM_001370627.1	Tmem267	0
chr12	80168001	80169000	1.1E-220	3.2E-217	0.312274970698117	XM_030246514.1	Actn1	0
chr7	63803001	63804000	2.6E-36	2.1E-33	-0.204278527680184	XR_882151.2	Gm39023	19238
chr11	32926001	32927000	1.2E-30	8.0E-28	0.208473223129205	NR_166841.1	Gm12111	0
chr14	34565001	34566000	2.0E-25	1.2E-22	-0.217464761222696	XM_006519019.4	Ldb3	0
chr4	152299001	152300000	1.4E-21	6.4E-19	-0.200688515184219	NR_161307.1	Icmt	0

Deregulated DNA regions 6 weeks 0.1Gy vs Sham

Chromosome	Start	End	P-Value	Q-Value	Difference	Nearest Transcript ID	Nearest Gene Name	Distance to Gene
chr5	127259001	127260000	7.2E-59	8.0E-57	-0.311230344589384	NR_038129.1	Tmem132cos	0
chr4	14845001	14846000	1.3E-54	1.3E-52	0.267604144830201	NM_028264.4	Pip4p2	18218

Deregulated DNA regions 6 weeks 2Gy vs Sham

Chromosome	Start	End	P-Value	Q-Value	Difference	Nearest Transcript ID	Nearest Gene Name	Distance to Gene
chr12	84571001	84572000	3.9E-196	1.6E-193	0.24016554160508	NM_007701.3	Vsx2	0
chr12	109033001	109034000	2.1E-191	7.8E-189	0.251598289405993	NM_001374200.1	Begain	0
chr5	5574001	5575000	1.7E-138	3.5E-136	0.458396855812132	NM_001384231.1	Gm8773	0
chr2	93647001	93648000	5.3E-71	5.1E-69	0.230782839058388	XR_374395.2	Alx4	0
chr9	30951001	30952000	2.3E-62	2.1E-60	-0.275002607774599	NM_013906.3	Adamts8	0
chr7	28747001	28748000	1.3E-52	1.1E-50	-0.221808268087047	NM_023637.3	Sars2	0
chr5	5573001	5574000	1.8E-49	1.3E-47	0.349727994623824	NM_001384231.1	Gm8773	3
chr12	108089001	108090000	1.8E-49	1.4E-47	-0.245196124493657	NM_028262.3	Setd3	16430
chr11	104186001	104187000	3.1E-46	2.2E-44	-0.242121848739496	NM_001082535.1	Sppl2c	0
chr9	20889001	20890000	2.0E-45	1.4E-43	-0.25908909447762	XR_003948524.1	LOC115487308	0
chr10	44447001	44448000	1.8E-44	1.3E-42	-0.239894766696985	XM_006512503.3	Prdm1	0
chr5	146736001	146737000	1.4E-42	9.8E-41	-0.214836993312109	NM_011669.3	Usp12	0
chr17	28007001	28008000	1.3E-41	8.9E-40	-0.257407384075227	XM_006524091.4	Anks1	0
chr4	150101001	150102000	4.6E-40	3.0E-38	-0.273420074349442	NM_177366.3	Gpr157	0
chr10	8263001	8264000	7.3E-40	4.8E-38	-0.316025641025641	XM_006512772.3	Ust	0
chr15	27508001	27509000	1.5E-39	1.0E-37	0.198655096247081	NM_020332.4	Ank	0
chr4	14845001	14846000	9.2E-38	5.8E-36	0.222516390396501	NM_028264.4	Pip4p2	18218
chr6	149353001	149354000	3.3E-37	2.1E-35	-0.246534470338571	XR_869628.2	Gm38930	265
chr1	152730001	152731000	9.6E-36	5.9E-34	-0.271104508607529	XM_006529266.4	Rgl1	0
chr4	142572001	142573000	1.2E-35	7.2E-34	-0.215161227733707	XR_003955304.1	Gm42342	58737
chr2	93648001	93649000	5.5E-35	3.3E-33	0.204183585578934	XR_374395.2	Alx4	0
chr9	54946001	54947000	1.1E-34	6.4E-33	-0.237996966653311	NM_177351.4	Hykk	0
chr5	128782001	128783000	8.1E-33	4.7E-31	-0.206942698847612	XM_006504295.4	Rimbp2	0
chr5	120627001	120628000	9.9E-33	5.7E-31	-0.204928194545322	NM_028041.2	Ddx54	0
chr2	165613001	165614000	2.5E-32	1.4E-30	-0.21815572161299	NM_001271963.1	Eya2	0
chr8	120523001	120524000	3.7E-32	2.1E-30	-0.199421568459175	NM_198671.2	Gse1	0
chr3	101455001	101456000	1.4E-31	7.7E-30	-0.212527492668622	NM_207205.2	Igsf3	0
chr4	107629001	107630000	4.6E-30	2.5E-28	-0.196186408678754	XM_006502975.1	Glis1	0
chr5	112719001	112720000	5.1E-30	2.8E-28	-0.246268712546852	XM_006535262.3	Myo18b	0

Chromosome	Start	End	P-Value	Q-Value	Difference	Nearest Transcript ID	Nearest Gene Name	Distance to Gene
chr4	120096001	120097000	3.1E-29	1.7E-27	-0.2221474999196	XM_011240453.2	Hivep3	0
chr11	43344001	43345000	2.0E-28	1.1E-26	-0.227905362199689	NR_029558.1	Mir146	29396
chr9	58439001	58440000	2.2E-28	1.2E-26	-0.233694556451613	NR_040736.1	4930461G14Rik	15172
chr12	108285001	108286000	3.3E-28	1.7E-26	-0.21071789149264	XM_006516158.4	Ccdc85c	7148
chr3	9624001	9625000	4.1E-28	2.2E-26	-0.209904474610357	NM_133218.2	Zfp704	13915
chr15	89385001	89386000	4.9E-28	2.6E-26	-0.221412555326878	NM_001160178.2	Klhdc7b	0
chr11	118330001	118331000	2.3E-27	1.2E-25	-0.219820260296617	NM_011594.3	Timp2	0
chr4	139499001	139500000	5.7E-27	2.9E-25	-0.19959561707886	NM_001160319.1	Ubr4	9467
chr19	47281001	47282000	6.5E-27	3.4E-25	-0.256251405058893	XM_006526669.4	Sh3pxd2a	0
chr18	66461001	66462000	1.5E-26	7.8E-25	0.204681786103804	NM_021451.2	Pmaip1	0
chr15	86277001	86278000	1.8E-26	9.0E-25	-0.217741211364617	NM_001358062.1	Tbc1d22a	0
chr8	120450001	120451000	2.1E-26	1.0E-24	-0.215341201889944	XM_006531147.4	Gse1	0
chr5	117572001	117573000	2.4E-26	1.2E-24	-0.210248008066103	XM_006530393.4	Ksr2	0
chr5	120179001	120180000	5.6E-26	2.8E-24	-0.23726856316297	NM_028762.1	Rbm19	0
chr12	84650001	84651000	6.5E-26	3.3E-24	-0.199458442642129	NM_001033776.2	Vrtn	0
chr10	38888001	38889000	9.0E-26	4.5E-24	-0.205756391529455	NM_001177783.1	Rfpl4b	66221
chr9	61439001	61440000	9.7E-26	4.9E-24	-0.243704198372669	XR_379516.1	Gm34322	2690
chr5	121982001	121983000	1.5E-25	7.2E-24	-0.20865630115248	XM_030254117.1	Cux2	0
chr4	141022001	141023000	2.2E-25	1.1E-23	-0.195197736055047	XR_001784132.2	Crocc	0
chr14	70508001	70509000	2.7E-25	1.3E-23	0.197140475763582	XM_030247614.1	Bmp1	0
chr16	94121001	94122000	6.4E-25	3.1E-23	-0.202449395542208	XM_006522968.3	Sim2	0
chr5	35073001	35074000	7.4E-25	3.5E-23	-0.238933827459644	XM_006503900.3	Dok7	0
chr15	31266001	31267000	7.4E-25	3.6E-23	-0.218045112781955	NM_146057.3	Dap	0
chr12	12685001	12686000	2.6E-24	1.2E-22	-0.236739713774598	XR_872489.1	Gm34696	2360
chr19	58219001	58220000	4.4E-24	2.0E-22	-0.204335976918376	XR_877965.3	Gm41875	11332
chr16	89513001	89514000	8.3E-24	3.8E-22	-0.222449151693714	NM_027771.1	Krtap7-1	4677
chr1	22296001	22297000	1.6E-23	7.5E-22	-0.219155296478978	NM_183018.4	Rims1	0
chr11	51410001	51411000	2.1E-23	9.7E-22	-0.220688337433423	NM_153393.2	Col23a1	0
chr17	87661001	87662000	9.6E-23	4.3E-21	-0.196189839572192	NM_008532.2	Epcam	9873
chr2	163879001	163880000	1.7E-22	7.6E-21	-0.195344734499452	NM_183023.2	Rims4	0

Chromosome	Start	End	P-Value	Q-Value	Difference	Nearest Transcript ID	Nearest Gene Name	Distance to Gene
chr9	77426001	77427000	3.2E-22	1.4E-20	-0.2264639874419	XM_030244218.1	Lrrc1	3820
chr5	124951001	124952000	7.0E-20	2.9E-18	-0.199079595599007	XR_868508.3	Gm40321	2249
chr9	79639001	79640000	4.7E-18	1.8E-16	-0.231482075780221	XM_006510797.4	Col12a1	0
chr19	6412001	6413000	6.6E-18	2.4E-16	-0.227408895994263	XR_879495.2	Gm14965	0
chr16	38636001	38637000	1.2E-17	4.5E-16	-0.210555188688264	XR_003951955.1	Gm36482	0
chr15	67040001	67041000	3.2E-17	1.2E-15	-0.204495325463067	XR_384160.3	Gm31342	0
chr15	100175001	100176000	4.3E-17	1.5E-15	0.198110084791947	NM_172819.3	Dip2b	0
chr8	72762001	72763000	2.7E-16	9.2E-15	-0.196816224537605	NM_007975.4	F2rl3	0
chr5	148965001	148966000	3.4E-16	1.2E-14	-0.235829931879923	XR_003956013.1	Gm15411	0
chr4	63256001	63257000	4.1E-16	1.4E-14	-0.207852395064209	Mir455_1	Mir455	0
chr15	88701001	88702000	4.7E-16	1.6E-14	-0.207097130387917	XR_384008.3	Brd1	0
chr17	45371001	45372000	6.3E-16	2.1E-14	-0.23392372684288	NM_152810.2	Cdc5l	19886
chr19	41287001	41288000	8.6E-16	2.8E-14	-0.197368653890393	NM_031376.4	Pik3ap1	0
chr1	34379001	34380000	4.3E-15	1.4E-13	-0.202198056580877	NR_039562.1	Mir5103	53120
chr8	87785001	87786000	8.8E-15	2.7E-13	-0.20460783079441	XM_030243876.1	Zfp423	0
chr7	142551001	142552000	5.2E-14	1.5E-12	-0.195873338016808	NR_002452.2	Nctc1	0
chr10	60935001	60936000	7.0E-13	1.9E-11	-0.204597244303127	XR_003948873.1	Gm26947	0
chr8	124306001	124307000	1.0E-12	2.6E-11	-0.206402094191999	NM_139272.2	Galnt2	0
chr10	88631001	88632000	4.3E-12	1.0E-10	-0.22689387956106	XR_001779416.2	Mybpc1	25771
chr12	70540001	70541000	5.8E-12	1.4E-10	-0.248539319656731	XR_381646.2	Gm32219	0
chr6	120173001	120174000	4.2E-10	8.3E-09	-0.198423423423423	NM_016718.2	Ninj2	0
chr11	118447001	118448000	1.5E-08	2.5E-07	-0.202535175560908	NR_045902.1	Gm11747	0
