# Supplementary material for: Multi-omics characterization of radiation-induced cerebellar remodeling and tumorigenic transcriptional programs
Source: Neoplasia. 2026 Jun 29;79:101333. doi: 10.1016/j.neo.2026.101333 (PMC13330529; doi:10.1016/j.neo.2026.101333)
Supplement: Supplementary file 11 [file mmc11.pdf]

Supplementary Table 2: Proteome

CB Proteome 1 week 0.1Gy vs Sham

Protein Accessions	Gene_name	log2(FC)	p-value	q-value	Protein Description
O35071	Kif1c	2.14	2.6E-11	4.0E-10	Kinesin-like protein KIF1C
Q9ER35	Fn3k	0.64	1.4E-09	7.4E-09	Fructosamine-3-kinase
Q9Z239	Fxyd1	-0.87	3.8E-08	9.8E-08	Phospholemman
Q08331	Calb2	-1.17	2.6E-07	4.8E-07	Calretinin
P19157	Gstp1	-1.14	5.3E-07	8.7E-07	Glutathione S-transferase P 1
P50114	S100b	-0.67	1.6E-06	2.3E-06	Protein S100-B
Q9JKL5	Tesc	-0.73	2.9E-06	3.8E-06	Calcineurin B homologous protein 3
P01863;P01865	Ighg;Igh-1a	0.80	3.5E-06	4.4E-06	Ig gamma-2A chain C region, A allele;Ig gamma-2A chain C region, membrane-bound form
Q9CR76	Tmem186	0.90	3.9E-06	4.8E-06	Transmembrane protein 186
Q8CC88	Vwa8	0.59	5.1E-06	6.0E-06	von Willebrand factor A domain-containing protein 8
Q9Z329	Itpr2	0.58	5.3E-06	6.3E-06	Inositol 1,4,5-trisphosphate receptor type 2
Q7TMB8	Cyfip1	0.55	9.8E-06	1.1E-05	Cytoplasmic FMR1-interacting protein 1
O55142	Rpl35a	1.76	1.4E-05	1.4E-05	60S ribosomal protein L35a
Q62388	Atm	0.57	1.4E-05	1.5E-05	Serine-protein kinase ATM
P02104	Hbb-y	-1.37	1.8E-05	1.8E-05	Hemoglobin subunit epsilon-Y2
Q9D173	Tomm7	-0.66	1.9E-05	1.9E-05	Mitochondrial import receptor subunit TOM7 homolog
A2AF47	Dock11	1.20	2.3E-05	2.2E-05	Dedicator of cytokinesis protein 11
Q45VK7	Dync2h1	0.57	2.8E-05	2.7E-05	Cytoplasmic dynein 2 heavy chain 1
Q9JLN9	Mtor	0.54	3.7E-05	3.4E-05	Serine/threonine-protein kinase mTOR
Q6P8J7	Ckmt2	0.83	3.7E-05	3.4E-05	Creatine kinase S-type, mitochondrial
P97313	Prkdc	0.55	3.7E-05	3.5E-05	DNA-dependent protein kinase catalytic subunit
Q9R0N9	Syt9	-0.71	4.9E-05	4.4E-05	Synaptotagmin-9
P23927	Cryab	0.78	5.3E-05	4.7E-05	Alpha-crystallin B chain
Q571H0	Urb1	0.75	5.4E-05	4.8E-05	Nucleolar pre-ribosomal-associated protein 1
P61264	Stx1b	-0.72	5.8E-05	5.1E-05	Syntaxin-1B
P70181	Pip5k1b	0.65	7.5E-05	6.4E-05	Phosphatidylinositol 4-phosphate 5-kinase type-1 beta
Q9JKC6	Cend1	-0.62	8.2E-05	6.8E-05	Cell cycle exit and neuronal differentiation protein 1
P63040	Cplx1	-0.72	1.0E-04	8.3E-05	Complexin-1
Q9JLC8	Sacs	0.55	1.1E-04	8.7E-05	Sacsin
Q9CXT7	Tmem192	0.55	1.5E-04	1.2E-04	Transmembrane protein 192
Q3UD82	Parp8	-0.86	2.2E-04	1.6E-04	Protein mono-ADP-ribosyltransferase PARP8
Q9CR37	Ppdpf	-0.64	2.6E-04	1.9E-04	Pancreatic progenitor cell differentiation and proliferation factor
Q91ZZ3	Sncb	-0.74	2.9E-04	2.1E-04	Beta-synuclein

Protein Accessions	Gene_name	log2(FC)	p-value	q-value	Protein Description
Q61696	Hspa1a	-0.89	3.2E-04	2.3E-04	Heat shock 70 kDa protein 1A
Q6DFV1	Ncapg2	0.54	3.7E-04	2.6E-04	Condensin-2 complex subunit G2
Q9JLY7	Dusp14	0.55	4.0E-04	2.8E-04	Dual specificity protein phosphatase 14
Q6PHN7	Tmem164	0.55	4.1E-04	2.8E-04	Transmembrane protein 164
Q80YQ2	Med23	0.68	4.2E-04	2.9E-04	Mediator of RNA polymerase II transcription subunit 23
Q62283	Tspan7	-1.58	4.8E-04	3.3E-04	Tetraspanin-7
Q9R269	Ppl	-0.98	5.3E-04	3.6E-04	Periplakin
Q8CHP6	Phc3	-1.45	5.4E-04	3.6E-04	Polyhomeotic-like protein 3
A2A559	Pgap3	0.57	5.4E-04	3.7E-04	Post-GPI attachment to proteins factor 3
Q99JB7	Amn	0.59	5.6E-04	3.8E-04	Protein amnionless
Q7TQF7	Amph	-0.93	6.9E-04	4.5E-04	Amphiphysin
Q9WV70	Noc2l	0.62	7.0E-04	4.6E-04	Nucleolar complex protein 2 homolog
P0DP60	Lynx1	-0.67	7.3E-04	4.7E-04	Ly-6/neurotoxin-like protein 1
P60603	Romo1	-1.17	9.9E-04	6.2E-04	Reactive oxygen species modulator 1
Q3TT38	Ilrun	-0.66	1.1E-03	7.0E-04	Protein ILRUN
Q9D6J5	Ndufb8	-0.94	1.1E-03	7.1E-04	NADH dehydrogenase [ubiquinone] 1 beta subcomplex subunit 8, mitochondrial
P48543	Kcnj9	-0.97	1.2E-03	7.2E-04	G protein-activated inward rectifier potassium channel 3
P14069	S100a6	-0.74	1.2E-03	7.3E-04	Protein S100-A6
Q8CB12	Gsdmc3	-0.63	1.2E-03	7.5E-04	Gasdermin-C3
O08539	Bin1	-0.68	1.3E-03	7.7E-04	Myc box-dependent-interacting protein 1
P61458	Pcbd1	-0.66	1.4E-03	8.3E-04	Pterin-4-alpha-carbinolamine dehydratase
Q9CWQ8	Castor1	0.65	1.4E-03	8.3E-04	Cytosolic arginine sensor for mTORC1 subunit 1
Q9D8T4	Tvp23b	-0.64	1.4E-03	8.3E-04	Golgi apparatus membrane protein TVP23 homolog B
P00416	mt-Co3	0.75	1.4E-03	8.5E-04	Cytochrome c oxidase subunit 3
Q63810	Ppp3r1	-0.70	1.4E-03	8.7E-04	Calcineurin subunit B type 1
Q9JK45	Kcnq5	0.54	1.6E-03	9.5E-04	Potassium voltage-gated channel subfamily KQT member 5
Q9JJR5	Akip1	-0.72	1.6E-03	9.6E-04	A-kinase-interacting protein 1
Q64704	Stx3	-0.74	1.8E-03	1.1E-03	Syntaxin-3
Q80Y14	Glrx5	-0.79	1.9E-03	1.1E-03	Glutaredoxin-related protein 5, mitochondrial
P38575	Upk2	-0.67	1.9E-03	1.1E-03	Uroplakin-2
Q8K4F6	Nsun5	0.68	2.2E-03	1.3E-03	Probable 28S rRNA (cytosine-C(5))-methyltransferase
Q9D8Y8	Ing5	-0.62	2.2E-03	1.3E-03	Inhibitor of growth protein 5
Q62093	Srsf2	-0.67	2.3E-03	1.3E-03	Serine/arginine-rich splicing factor 2
Q8BK30	Ndufv3	-1.02	2.4E-03	1.4E-03	NADH dehydrogenase [ubiquinone] flavoprotein 3, mitochondrial
P0DP26;P0DP27;P0DP28	Calm1;Calm2;Calm3	-0.80	2.6E-03	1.5E-03	Calmodulin-1;Calmodulin-2;Calmodulin-3
Q00897	Serpina1d	-0.88	2.6E-03	1.5E-03	Alpha-1-antitrypsin 1-4

Protein Accessions	Gene_name	log2(FC)	p-value	q-value	Protein Description
P56212	Arpp19	-0.64	2.6E-03	1.5E-03	cAMP-regulated phosphoprotein 19
Q9CQ19	Myl9	-0.63	3.2E-03	1.8E-03	Myosin regulatory light polypeptide 9
Q9QY76	Vapb	-0.63	3.8E-03	2.1E-03	Vesicle-associated membrane protein-associated protein B
Q62252	Spa17	-0.64	3.9E-03	2.1E-03	Sperm surface protein Sp17
Q921H9	Coa7	-0.79	4.3E-03	2.3E-03	Cytochrome c oxidase assembly factor 7
Q6T264	Maml1	-0.90	4.3E-03	2.3E-03	Mastermind-like protein 1
Q8VHN7	Adgrv1	-0.66	4.4E-03	2.3E-03	Adhesion G-protein coupled receptor V1
P63054	Pcp4	-0.62	4.7E-03	2.5E-03	Calmodulin regulator protein PCP4
Q9R0P4	Smap	-1.56	4.7E-03	2.5E-03	Small acidic protein
Q6P069	Sri	-0.63	4.8E-03	2.6E-03	Sorcin
Q9CR20	Ier3ip1	-1.48	5.0E-03	2.6E-03	Immediate early response 3-interacting protein 1
P08207	S100a10	-0.73	5.0E-03	2.6E-03	Protein S100-A10
Q3UFS4	Gpatch11	-0.62	5.8E-03	3.0E-03	G patch domain-containing protein 11
Q811W2	Cyp26b1	0.75	6.1E-03	3.2E-03	Cytochrome P450 26B1
Q9Z0F7	Sncg	-0.63	6.4E-03	3.3E-03	Gamma-synuclein
Q8JZX9	Cdc42ep2	-0.95	6.6E-03	3.4E-03	Cdc42 effector protein 2
P13808	Slc4a2	-0.78	7.2E-03	3.6E-03	Anion exchange protein 2
Q8BSU7	Mob3a	-1.38	7.2E-03	3.6E-03	MOB kinase activator 3A
P51859	Hdgf	-0.61	7.3E-03	3.7E-03	Hepatoma-derived growth factor
Q9D7S9	Chmp5	-0.63	7.7E-03	3.9E-03	Charged multivesicular body protein 5
P04104	Krt1	-0.73	8.0E-03	4.0E-03	Keratin, type II cytoskeletal 1
O08740	Polr2j	-0.65	8.3E-03	4.1E-03	DNA-directed RNA polymerase II subunit RPB11
Q9JMG7	Hdgfl3	-0.64	8.3E-03	4.1E-03	Hepatoma-derived growth factor-related protein 3
Q9WU63	Hebp2	-0.76	8.7E-03	4.3E-03	Heme-binding protein 2
Q9WTZ8	Bex2	-0.63	9.9E-03	4.8E-03	Protein BEX2
Q8C0C4	Ccser1	-0.78	9.9E-03	4.8E-03	Serine-rich coiled-coil domain-containing protein 1
Q61206	Pafah1b2	-0.78	1.0E-02	4.9E-03	Platelet-activating factor acetylhydrolase IB subunit beta
Q9D8B3	Chmp4b	-0.64	1.0E-02	4.9E-03	Charged multivesicular body protein 4b
Q80VJ2	Sra1	-0.65	1.1E-02	5.2E-03	Steroid receptor RNA activator 1
O89116	Vti1a	-0.70	1.1E-02	5.2E-03	Vesicle transport through interaction with t-SNAREs homolog 1A
P63248	Pkia	-0.66	1.1E-02	5.3E-03	cAMP-dependent protein kinase inhibitor alpha
Q9CQ13	Coprs	-1.57	1.2E-02	5.6E-03	Coordinator of PRMT5 and differentiation stimulator
Q62393	Tpd52	-0.62	1.2E-02	5.7E-03	Tumor protein D52
O35717	Socs2	-1.75	1.2E-02	5.8E-03	Suppressor of cytokine signaling 2
O55042	Snca	-0.65	1.2E-02	5.8E-03	Alpha-synuclein
P61216	Taf13	-0.66	1.3E-02	5.9E-03	Transcription initiation factor TFIID subunit 13

Protein Accessions	Gene_name	log2(FC)	p-value	q-value	Protein Description
Q8VEE0	Rpe	-0.65	1.3E-02	6.3E-03	Ribulose-phosphate 3-epimerase
O08997	Atox1	-0.63	1.4E-02	6.4E-03	Copper transport protein ATOX1
Q3UHX2	Pdap1	-0.63	1.4E-02	6.4E-03	28 kDa heat- and acid-stable phosphoprotein
P21107	Tpm3	-0.65	1.4E-02	6.6E-03	Tropomyosin alpha-3 chain
P15066	Jund	-0.74	1.4E-02	6.8E-03	Transcription factor jun-D
P56390	Cks2	-0.75	1.5E-02	6.8E-03	Cyclin-dependent kinases regulatory subunit 2
Q9D0V7	Ebag9	-0.65	1.5E-02	7.0E-03	Receptor-binding cancer antigen expressed on SiSo cells
Q921K9	Bcl7b	-0.93	1.5E-02	7.1E-03	B-cell CLL/lymphoma 7 protein family member B
Q6PGD0	Atraid	-0.81	1.5E-02	7.1E-03	All-trans retinoic acid-induced differentiation factor
Q9CQ39	Med21	-0.65	1.6E-02	7.2E-03	Mediator of RNA polymerase II transcription subunit 21
Q9CWZ3	Rbm8a	-0.72	1.6E-02	7.5E-03	RNA-binding protein 8A
Q60867	Neurod1	-0.63	1.6E-02	7.5E-03	Neurogenic differentiation factor 1
Q61048	Wbp4	-0.62	1.6E-02	7.5E-03	WW domain-binding protein 4
Q80Y55	Bsdc1	-0.74	1.7E-02	7.6E-03	BSD domain-containing protein 1
Q62313	Tgoln1	-0.75	1.7E-02	7.8E-03	Trans-Golgi network integral membrane protein 1
Q2EMV9	Parp14	-0.67	1.7E-02	7.8E-03	Protein mono-ADP-ribosyltransferase PARP14
Q91WK5	Gcsh	-0.63	1.7E-02	7.9E-03	Glycine cleavage system H protein, mitochondrial
P70274	Selenop	-0.72	1.7E-02	7.9E-03	Selenoprotein P
P18572	Bsg	-0.62	1.8E-02	8.3E-03	Basigin
Q9CZG9	Pdzd11	-0.78	1.9E-02	8.6E-03	PDZ domain-containing protein 11
P62774	Mtpn	-0.60	1.9E-02	8.6E-03	Myotrophin
Q9WTQ5	Akap12	-0.64	1.9E-02	8.6E-03	A-kinase anchor protein 12
Q9DAT2	Mrgbp	-0.74	1.9E-02	8.6E-03	MRG/MORF4L-binding protein
Q99LB0	Dnttip1	-0.66	2.0E-02	9.1E-03	Deoxynucleotidyltransferase terminal-interacting protein 1
P11087	Col1a1	-0.62	2.1E-02	9.3E-03	Collagen alpha-1(I) chain
Q8C7Q4	Rbm4	-1.39	2.1E-02	9.4E-03	RNA-binding protein 4
Q9D824	Fip1l1	-0.66	2.2E-02	9.6E-03	Pre-mRNA 3'-end-processing factor FIP1
P47955	Rplp1	-0.62	2.3E-02	1.0E-02	60S acidic ribosomal protein P1
Q8R3E3	Wipi1	-0.79	2.4E-02	1.0E-02	WD repeat domain phosphoinositide-interacting protein 1
Q9CQM2	Kdelr2	0.72	2.4E-02	1.0E-02	ER lumen protein-retaining receptor 2
Q9CYA6	Zcchc8	-0.61	2.4E-02	1.1E-02	Zinc finger CCHC domain-containing protein 8
Q8K5B2	Mcfd2	-0.92	2.4E-02	1.1E-02	Multiple coagulation factor deficiency protein 2 homolog
Q8BRH3	Arhgap19	-0.72	2.5E-02	1.1E-02	Rho GTPase-activating protein 19
Q78ZA7	Nap1l4	-0.65	2.5E-02	1.1E-02	Nucleosome assembly protein 1-like 4
Q00420	Gabpb1	-0.71	2.5E-02	1.1E-02	GA-binding protein subunit beta-1
Q9CQH7	Btf3l4	-0.61	2.5E-02	1.1E-02	Transcription factor BTF3 homolog 4

Protein Accessions	Gene_name	log2(FC)	p-value	q-value	Protein Description
P84089	Erh	-0.68	2.6E-02	1.1E-02	Enhancer of rudimentary homolog
Q9D7M8	Polr2d	-0.64	2.6E-02	1.1E-02	DNA-directed RNA polymerase II subunit RPB4
Q62190	Mst1r	-2.53	2.6E-02	1.1E-02	Macrophage-stimulating protein receptor
Q9D8C8	Ppp1r35	-0.69	2.7E-02	1.1E-02	Protein phosphatase 1 regulatory subunit 35
Q3TQI7	NaN	-0.65	2.7E-02	1.2E-02	Telomere length and silencing protein 1 homolog
Q8BH75	Rnf41	-0.68	2.7E-02	1.2E-02	E3 ubiquitin-protein ligase NRDP1
Q80U04	Pja2	-0.63	2.8E-02	1.2E-02	E3 ubiquitin-protein ligase Praja-2
Q8BP92	Rcn2	-0.78	2.8E-02	1.2E-02	Reticulocalbin-2
Q99JH1	Rpp25l	0.96	2.8E-02	1.2E-02	Ribonuclease P protein subunit p25-like protein
Q8R0P8	Abhd8	0.55	2.8E-02	1.2E-02	Protein ABHD8
Q9DAY5	NaN	-0.72	2.8E-02	1.2E-02	UPF0669 protein C6orf120 homolog
Q6PAL1	Draxin	-0.67	2.8E-02	1.2E-02	Draxin
O70494	Sp3	-0.98	2.9E-02	1.2E-02	Transcription factor Sp3
Q8BHB9	Clic6	-1.19	2.9E-02	1.2E-02	Chloride intracellular channel protein 6
Q9CQK7	Rwdd1	-0.64	2.9E-02	1.2E-02	RWD domain-containing protein 1
Q91Z38	Ttc1	-0.64	2.9E-02	1.2E-02	Tetratricopeptide repeat protein 1
Q3THE2	Myl12b	-0.68	2.9E-02	1.2E-02	Myosin regulatory light chain 12B
Q9QXP6	Mkrn1	-0.63	2.9E-02	1.2E-02	E3 ubiquitin-protein ligase makorin-1
Q3TXT3	Inip	-0.74	2.9E-02	1.2E-02	SOSS complex subunit C
Q3U898	Cops9	-0.65	2.9E-02	1.3E-02	COP9 signalosome complex subunit 9
Q80UU9	Pgrmc2	-0.67	2.9E-02	1.3E-02	Membrane-associated progesterone receptor component 2
Q5SQF8	Sap30l	-0.60	3.0E-02	1.3E-02	Histone deacetylase complex subunit SAP30L
Q9WU01	Khdrbs2	-0.64	3.0E-02	1.3E-02	KH domain-containing, RNA-binding, signal transduction-associated protein 2
P34928	Apoc1	-0.70	3.0E-02	1.3E-02	Apolipoprotein C-I
Q9D554	Sf3a3	-0.60	3.0E-02	1.3E-02	Splicing factor 3A subunit 3
P63147	Ube2b	-0.68	3.1E-02	1.3E-02	Ubiquitin-conjugating enzyme E2 B
Q9WV03	Fam50a	-0.64	3.1E-02	1.3E-02	Protein FAM50A
Q61189	Clns1a	-0.63	3.1E-02	1.3E-02	Methylosome subunit pICln
Q9CWK3	Cd2bp2	-0.72	3.1E-02	1.3E-02	CD2 antigen cytoplasmic tail-binding protein 2
Q8R1N0	Znf830	-0.65	3.2E-02	1.3E-02	Zinc finger protein 830
Q99LW6	Yaf2	-1.09	3.2E-02	1.3E-02	YY1-associated factor 2
P54728	Rad23b	-0.64	3.2E-02	1.4E-02	UV excision repair protein RAD23 homolog B
Q60865	Caprin1	-0.65	3.4E-02	1.4E-02	Caprin-1
P58044	Idi1	-0.62	3.5E-02	1.5E-02	Isopentenyl-diphosphate Delta-isomerase 1
Q921W0	Chmp1a	-0.63	3.6E-02	1.5E-02	Charged multivesicular body protein 1a
Q61937	Npm1	-0.74	3.6E-02	1.5E-02	Nucleophosmin

Protein Accessions	Gene_name	log2(FC)	p-value	q-value	Protein Description
P21995	Emb	-0.63	3.7E-02	1.5E-02	Embigin
Q60973	Rbbp7	-0.63	3.7E-02	1.5E-02	Histone-binding protein RBBP7
Q9CR35	Ctrb1	-0.64	3.9E-02	1.6E-02	Chymotrypsinogen B
Q9CXU9	Eif1b	-0.67	3.9E-02	1.6E-02	Eukaryotic translation initiation factor 1b
Q9WU00	Nrf1	-0.73	3.9E-02	1.6E-02	Nuclear respiratory factor 1
Q9D6V8	Paip2	-0.78	4.0E-02	1.6E-02	Polyadenylate-binding protein-interacting protein 2
Q60772	Cdkn2c	-0.75	4.1E-02	1.7E-02	Cyclin-dependent kinase 4 inhibitor C
Q9ERD8	Parvg	-0.61	4.1E-02	1.7E-02	Gamma-parvin
Q60972	Rbbp4	-0.97	4.1E-02	1.7E-02	Histone-binding protein RBBP4
Q8BW22	Ss18l1	-0.61	4.1E-02	1.7E-02	Calcium-responsive transactivator
Q8BIS8	Ccdc126	-0.63	4.3E-02	1.7E-02	Coiled-coil domain-containing protein 126
Q9QXV3	Ing1	-0.68	4.4E-02	1.8E-02	Inhibitor of growth protein 1
P0CG49;P0CG50	Ubb;Ubc	-0.99	4.4E-02	1.8E-02	Polyubiquitin-B;Polyubiquitin-C
Q61391	Mme	-0.61	4.6E-02	1.8E-02	Neprilysin
E9PV86	Mctp1	-1.40	4.6E-02	1.8E-02	Multiple C2 and transmembrane domain-containing protein 1
P28656	Nap1l1	-0.67	4.8E-02	1.9E-02	Nucleosome assembly protein 1-like 1
P70339;Q8K025	Frat1;Frat2	-0.64	4.8E-02	1.9E-02	Proto-oncogene FRAT1;GSK-3-binding protein FRAT2
Q03958	Pfdn6	-0.60	4.8E-02	1.9E-02	Prefoldin subunit 6
O88811	Stam2	-0.61	4.8E-02	1.9E-02	Signal transducing adapter molecule 2
Q8CH62	Lto1	-0.78	4.9E-02	1.9E-02	Protein LTO1 homolog
Q9D593	Atp6v1e2	0.65	4.9E-02	1.9E-02	V-type proton ATPase subunit E 2
Q61205	Pafah1b3	-0.62	5.0E-02	2.0E-02	Platelet-activating factor acetylhydrolase IB subunit gamma
Q9JM83	Calm4	-2.01	5.2E-02	2.0E-02	Calmodulin-4
P07309	Ttr	-0.64	5.2E-02	2.1E-02	Transthyretin
Q9CZB6	Med7	-0.63	5.2E-02	2.1E-02	Mediator of RNA polymerase II transcription subunit 7
P62311	Lsm3	-0.79	5.3E-02	2.1E-02	U6 snRNA-associated Sm-like protein LSm3
Q05722	Col9a1	-0.62	5.4E-02	2.1E-02	Collagen alpha-1(IX) chain
P99027	Rplp2	-0.69	5.5E-02	2.2E-02	60S acidic ribosomal protein P2
Q00915	Rbp1	-0.75	5.6E-02	2.2E-02	Retinol-binding protein 1
Q05186	Rcn1	-0.66	5.7E-02	2.2E-02	Reticulocalbin-1
Q01147	Creb1	-0.78	6.0E-02	2.3E-02	Cyclic AMP-responsive element-binding protein 1
Q8K3P5	Cnot6	0.55	6.0E-02	2.3E-02	CCR4-NOT transcription complex subunit 6
Q01815	Cacna1c	-0.72	6.4E-02	2.5E-02	Voltage-dependent L-type calcium channel subunit alpha-1C
Q7TQA1	Igsf1	-0.72	6.4E-02	2.5E-02	Immunoglobulin superfamily member 1
Q02013	Aqp1	-0.62	6.4E-02	2.5E-02	Aquaporin-1
Q99JP4	Cdc26	-0.63	6.5E-02	2.5E-02	Anaphase-promoting complex subunit CDC26

Protein Accessions	Gene_name	log2(FC)	p-value	q-value	Protein Description
Q3TAS6	Emc10	-0.75	6.6E-02	2.5E-02	ER membrane protein complex subunit 10
Q9CPU2	Ndufb2	-0.74	6.6E-02	2.5E-02	NADH dehydrogenase [ubiquinone] 1 beta subcomplex subunit 2, mitochondrial
Q8VCE4	NaN	-0.62	6.6E-02	2.5E-02	Uncharacterized protein C9orf40 homolog
Q8BP27	Sfr1	-0.66	6.6E-02	2.5E-02	Swi5-dependent recombination DNA repair protein 1 homolog
Q9JJ93	MNCb-2990	-0.75	7.2E-02	2.7E-02	Uncharacterized protein C14orf119 homolog
P55821	Stmn2	-0.61	7.5E-02	2.8E-02	Stathmin-2
Q8BGZ2	Fam168a	-0.60	7.6E-02	2.9E-02	Protein FAM168A
P97825	Jpt1	-0.62	8.0E-02	3.0E-02	Jupiter microtubule associated homolog 1
Q8R4S0	Ppp1r14c	-0.62	8.2E-02	3.0E-02	Protein phosphatase 1 regulatory subunit 14C
E9QAM5	Helz2	-0.69	8.4E-02	3.1E-02	Helicase with zinc finger domain 2
Q9DCB1	Hmgn3	-0.89	1.1E-01	3.8E-02	High mobility group nucleosome-binding domain-containing protein 3
Q920F6	Smc1b	1.04	1.1E-01	3.9E-02	Structural maintenance of chromosomes protein 1B
Q8BGH7	Cdc42se2	-0.64	1.1E-01	3.9E-02	CDC42 small effector protein 2
Q8K1L2	Spin4	0.72	1.1E-01	4.1E-02	Spindlin-4
Q80V24	Vgll4	-0.62	1.3E-01	4.4E-02	Transcription cofactor vestigial-like protein 4
Q9D032	Ssbp3	-0.69	1.4E-01	4.7E-02	Single-stranded DNA-binding protein 3
P28667	Marcksl1	-0.96	1.4E-01	4.8E-02	MARCKS-related protein
Q8C1M2	Znf428	-0.78	1.4E-01	4.8E-02	Zinc finger protein 428
Q9JJ11	Tacc3	-0.61	1.5E-01	5.0E-02	Transforming acidic coiled-coil-containing protein 3
A3KG59	Pm20d2	-0.67	1.5E-01	5.2E-02	Peptidase M20 domain-containing protein 2
P0C8B4	Gon7	-0.66	1.7E-01	5.6E-02	EKC/KEOPS complex subunit GON7
Q8BMK5	Ccdc184	-0.68	1.8E-01	6.0E-02	Coiled-coil domain-containing protein 184
Q80YA8	Crb2	-0.70	1.8E-01	6.0E-02	Protein crumbs homolog 2
O08918	Ccng2	-0.63	1.8E-01	6.1E-02	Cyclin-G2
Q5I1X5	Ppp1r13l	-0.85	2.0E-01	6.5E-02	RelA-associated inhibitor
Q8R1B0	Stac2	0.70	2.1E-01	6.8E-02	SH3 and cysteine-rich domain-containing protein 2
Q6P1G6	Fam167a	-0.65	2.1E-01	7.0E-02	Protein FAM167A
Q61781	Krt14	-1.96	2.2E-01	7.0E-02	Keratin, type I cytoskeletal 14
A4Q9F6	Ttll13	0.66	2.3E-01	7.4E-02	Tubulin polyglutamylase TTLL13
Q80UZ0	Fgd5	-2.71	2.5E-01	7.9E-02	FYVE, RhoGEF and PH domain-containing protein 5
Q9D8Z2	Triap1	-0.64	2.7E-01	8.5E-02	TP53-regulated inhibitor of apoptosis 1
Q80TT2	Baiap3	-0.73	2.9E-01	9.1E-02	BAI1-associated protein 3
Q8R344	Ccdc12	0.57	3.0E-01	9.4E-02	Coiled-coil domain-containing protein 12
Q91ZE5	Adgre4	-0.91	3.6E-01	1.1E-01	Adhesion G protein-coupled receptor E4
Q9EQU3	Tlr9	-0.66	3.8E-01	1.1E-01	Toll-like receptor 9
B1AVH7	Tbc1d2	0.62	4.3E-01	1.3E-01	TBC1 domain family member 2A

Protein Accessions	Gene_name	log2(FC)	p-value	q-value	Protein Description
Q9Z126	Pf4	0.73	5.3E-01	1.5E-01	Platelet factor 4
Q8K207	NaN	0.61	6.3E-01	1.7E-01	Uncharacterized protein C1orf21 homolog
P46425	Gstp2	-0.91	6.5E-01	1.8E-01	Glutathione S-transferase P 2

CB Proteome 1 week 2Gy vs Sham

Protein Accessions	Gene_name	log2(FC)	p-value	q-value	Protein Description
P11881	Itpr1	0.67	1.9E-09	9.5E-09	Inositol 1,4,5-trisphosphate receptor type 1
Q8BH86	Dglucy	0.63	3.7E-09	1.6E-08	D-glutamate cyclase, mitochondrial
Q9ER35	Fn3k	0.62	2.2E-08	6.4E-08	Fructosamine-3-kinase
Q8BVD5	Mpp7	0.58	3.9E-08	1.0E-07	MAGUK p55 subfamily member 7
Q8VE38	Oxnad1	0.56	5.2E-08	1.3E-07	Oxidoreductase NAD-binding domain-containing protein 1
Q9Z239	Fxyd1	-0.67	2.0E-07	3.8E-07	Phospholemman
O35071	Kif1c	2.76	2.3E-07	4.4E-07	Kinesin-like protein KIF1C
Q9JKL5	Tesc	-0.78	3.0E-07	5.4E-07	Calcineurin B homologous protein 3
Q5EBJ4	Ermn	-0.72	9.5E-07	1.4E-06	Ermin
Q62283	Tspan7	-2.67	1.2E-06	1.7E-06	Tetraspanin-7
Q9Z268	Rasal1	0.65	1.3E-06	1.8E-06	RasGAP-activating-like protein 1
Q8BVW0	Ganc	0.59	1.4E-06	2.0E-06	Neutral alpha-glucosidase C
Q9CWY3	Setd6	0.54	2.3E-06	3.0E-06	N-lysine methyltransferase SETD6
Q8CC88	Vwa8	0.68	2.3E-06	3.1E-06	von Willebrand factor A domain-containing protein 8
Q9Z329	Itpr2	0.63	2.6E-06	3.4E-06	Inositol 1,4,5-trisphosphate receptor type 2
Q08331	Calb2	-0.74	4.0E-06	4.9E-06	Calretinin
Q8CCT4	Tceal5	-0.61	7.5E-06	8.5E-06	Transcription elongation factor A protein-like 5
Q45VK7	Dync2h1	0.61	9.1E-06	1.0E-05	Cytoplasmic dynein 2 heavy chain 1
Q62388	Atm	0.57	1.1E-05	1.2E-05	Serine-protein kinase ATM
Q9WUJ8	Orc6	0.58	1.1E-05	1.2E-05	Origin recognition complex subunit 6
Q9CR76	Tmem186	0.95	1.2E-05	1.2E-05	Transmembrane protein 186
P16460	Ass1	0.69	1.3E-05	1.4E-05	Argininosuccinate synthase
P31725	S100a9	-0.76	1.4E-05	1.5E-05	Protein S100-A9
Q9R0N9	Syt9	-1.04	1.8E-05	1.8E-05	Synaptotagmin-9
Q8R4G0	Ntng1	0.66	2.5E-05	2.4E-05	Netrin-G1
P19157	Gstp1	-0.87	2.5E-05	2.5E-05	Glutathione S-transferase P 1
P61264	Stx1b	-0.85	2.8E-05	2.7E-05	Syntaxin-1B
P02104	Hbb-y	-1.18	2.8E-05	2.7E-05	Hemoglobin subunit epsilon-Y2
Q9CQ91	Ndufa3	1.06	3.0E-05	2.8E-05	NADH dehydrogenase [ubiquinone] 1 alpha subcomplex subunit 3
O55142	Rpl35a	1.95	3.8E-05	3.5E-05	60S ribosomal protein L35a

Protein Accessions	Gene_name	log2(FC)	p-value	q-value	Protein Description
P63040	Cplx1	-0.86	3.8E-05	3.5E-05	Complexin-1
Q3THF9	Coq10b	0.65	4.1E-05	3.7E-05	Coenzyme Q-binding protein COQ10 homolog B, mitochondrial
Q9JLC8	Sacs	0.58	4.8E-05	4.3E-05	Sacsin
A8C756	Thada	0.56	5.6E-05	4.9E-05	Thyroid adenoma-associated protein homolog
Q571H0	Urb1	0.75	6.5E-05	5.6E-05	Nucleolar pre-ribosomal-associated protein 1
Q62313	Tgoln1	-1.21	6.8E-05	5.8E-05	Trans-Golgi network integral membrane protein 1
Q9Z2A7	Dgat1	0.56	7.4E-05	6.3E-05	Diacylglycerol O-acyltransferase 1
Q8JZS0	Lin7a	-0.67	7.5E-05	6.4E-05	Protein lin-7 homolog A
Q9CQE3	Mrps17	0.57	9.9E-05	8.1E-05	28S ribosomal protein S17, mitochondrial
Q8CHP6	Phc3	-2.38	1.0E-04	8.5E-05	Polyhomeotic-like protein 3
Q504N2	Slc22a15	0.66	1.2E-04	9.5E-05	Solute carrier family 22 member 15
Q99N28	Cadm3	-0.63	1.3E-04	1.0E-04	Cell adhesion molecule 3
Q80YQ2	Med23	0.66	1.5E-04	1.2E-04	Mediator of RNA polymerase II transcription subunit 23
P01786	NaN	0.57	2.5E-04	1.8E-04	Ig heavy chain V region MOPC 47A
Q8CDJ8	Ston1	0.60	2.6E-04	1.9E-04	Stonin-1
Q6P8J7	Ckmt2	0.57	2.7E-04	1.9E-04	Creatine kinase S-type, mitochondrial
P60603	Romo1	-1.61	3.0E-04	2.1E-04	Reactive oxygen species modulator 1
Q9D6I9	Lurap1	-0.68	3.1E-04	2.2E-04	Leucine rich adaptor protein 1
Q6PHN7	Tmem164	0.55	3.5E-04	2.5E-04	Transmembrane protein 164
Q9D173	Tomm7	-0.72	3.7E-04	2.6E-04	Mitochondrial import receptor subunit TOM7 homolog
Q9JLY7	Dusp14	0.56	4.0E-04	2.8E-04	Dual specificity protein phosphatase 14
Q6DFV1	Ncapg2	0.60	4.0E-04	2.8E-04	Condensin-2 complex subunit G2
Q8CHC8	Adnp2	0.61	4.0E-04	2.8E-04	Activity-dependent neuroprotector homeobox protein 2
P0C7L0	Wipf3	-0.63	4.6E-04	3.2E-04	WAS/WASL-interacting protein family member 3
Q9Z0H1	Wdr46	0.57	4.8E-04	3.3E-04	WD repeat-containing protein 46
Q99J21	Mcoln1	-0.77	5.1E-04	3.5E-04	Mucolipin-1
P17095	Hmga1	-0.76	5.5E-04	3.7E-04	High mobility group protein HMG-I/HMG-Y
P09066	En2	-0.77	5.7E-04	3.8E-04	Homeobox protein engrailed-2
P27005	S100a8	-0.65	6.0E-04	4.0E-04	Protein S100-A8
P09041	Pgk2	0.62	6.0E-04	4.0E-04	Phosphoglycerate kinase 2
Q9JKC6	Cend1	-0.63	6.1E-04	4.0E-04	Cell cycle exit and neuronal differentiation protein 1
Q7TQF7	Amph	-0.94	6.8E-04	4.4E-04	Amphiphysin
Q9JHT5	Ammecr1	0.60	6.9E-04	4.5E-04	AMME syndrome candidate gene 1 protein homolog
Q91W69	Epn3	-0.67	7.4E-04	4.8E-04	Epsin-3
P38575	Upk2	-0.75	7.4E-04	4.8E-04	Uroplakin-2
Q8C8M1	Sinhcaf	0.54	8.7E-04	5.6E-04	SIN3-HDAC complex-associated factor

Protein Accessions	Gene_name	log2(FC)	p-value	q-value	Protein Description
Q3UD82	Parp8	-0.80	1.0E-03	6.4E-04	Protein mono-ADP-ribosyltransferase PARP8
Q9WV70	Noc2l	0.63	1.0E-03	6.4E-04	Nucleolar complex protein 2 homolog
Q9R0P4	Smap	-2.19	1.1E-03	6.7E-04	Small acidic protein
Q62445	Sp4	-0.63	1.1E-03	6.7E-04	Transcription factor Sp4
Q9CR20	Ier3ip1	-1.90	1.3E-03	7.7E-04	Immediate early response 3-interacting protein 1
P0DP26;P0DP27;P0DP28	Calm1;Calm2;Calm3	-0.90	1.3E-03	8.0E-04	Calmodulin-1;Calmodulin-2;Calmodulin-3
Q921L3	Tmco1	0.55	1.4E-03	8.7E-04	Calcium load-activated calcium channel
Q921K9	Bcl7b	-1.41	1.4E-03	8.7E-04	B-cell CLL/lymphoma 7 protein family member B
Q63912	Omg	-0.65	1.5E-03	9.2E-04	Oligodendrocyte-myelin glycoprotein
P0DP60	Lynx1	-0.67	1.7E-03	1.0E-03	Ly-6/neurotoxin-like protein 1
Q63810	Ppp3r1	-0.69	1.8E-03	1.1E-03	Calcineurin subunit B type 1
Q61010	Dtx1	0.72	1.9E-03	1.1E-03	E3 ubiquitin-protein ligase DTX1
O88643	Pak1	-0.60	1.9E-03	1.1E-03	Serine/threonine-protein kinase PAK 1
Q9CXT7	Tmem192	0.59	2.0E-03	1.2E-03	Transmembrane protein 192
Q64704	Stx3	-0.66	2.2E-03	1.2E-03	Syntaxin-3
P32114	Pax2	-0.65	2.5E-03	1.4E-03	Paired box protein Pax-2
O08539	Bin1	-0.62	2.7E-03	1.5E-03	Myc box-dependent-interacting protein 1
A2A5Z6	Smurf2	0.68	2.9E-03	1.6E-03	E3 ubiquitin-protein ligase SMURF2
Q8BSU7	Mob3a	-1.41	3.1E-03	1.7E-03	MOB kinase activator 3A
Q8K4F6	Nsun5	0.57	3.1E-03	1.7E-03	Probable 28S rRNA (cytosine-C(5))-methyltransferase
Q8C0C4	Ccser1	-0.99	3.2E-03	1.8E-03	Serine-rich coiled-coil domain-containing protein 1
Q99P65	Slc29a3	0.55	3.2E-03	1.8E-03	Equilibrative nucleoside transporter 3
Q9CQ13	Coprs	-2.15	3.4E-03	1.9E-03	Coordinator of PRMT5 and differentiation stimulator
Q7TNE3	Spag7	-0.62	3.6E-03	2.0E-03	Sperm-associated antigen 7
Q99NF8	Ranbp17	0.73	4.1E-03	2.2E-03	Ran-binding protein 17
Q7TNR6	Igsf21	-0.63	4.2E-03	2.2E-03	Immunoglobulin superfamily member 21
P58269	Dpf3	-0.62	4.2E-03	2.3E-03	Zinc finger protein DPF3
Q9CX66	D10Wsu102e	-0.73	4.3E-03	2.3E-03	Uncharacterized protein C12orf45 homolog
P13516	Scd1	0.55	4.5E-03	2.4E-03	Acyl-CoA desaturase 1
Q64707	Zrsr1	-0.67	4.5E-03	2.4E-03	U2 small nuclear ribonucleoprotein auxiliary factor 35 kDa subunit-related protein 1
Q6P069	Sri	-0.65	4.5E-03	2.4E-03	Sorcin
A2A559	Pgap3	0.63	4.6E-03	2.4E-03	Post-GPI attachment to proteins factor 3
P51859	Hdgf	-0.68	4.7E-03	2.5E-03	Hepatoma-derived growth factor
O55003	Bnip3	-0.88	5.0E-03	2.6E-03	BCL2/adenovirus E1B 19 kDa protein-interacting protein 3
Q9JJZ6	Klf13	-0.68	5.2E-03	2.7E-03	Krueppel-like factor 13
Q99JF8	Psip1	-0.62	5.3E-03	2.8E-03	PC4 and SFRS1-interacting protein

Protein Accessions	Gene_name	log2(FC)	p-value	q-value	Protein Description
Q3UFS4	Gpatch11	-0.66	5.3E-03	2.8E-03	G patch domain-containing protein 11
P56484	Ccr8	-0.86	5.3E-03	2.8E-03	C-C chemokine receptor type 8
O88992	C1ql1	-0.62	5.3E-03	2.8E-03	C1q-related factor
Q8BR63	Fam177a1	-0.80	5.4E-03	2.8E-03	Protein FAM177A1
Q8CB12	Gsdmc3	-0.66	5.4E-03	2.8E-03	Gasdermin-C3
Q3ULM0	Ccdc106	-0.85	5.4E-03	2.8E-03	Coiled-coil domain-containing protein 106
Q8C7Q4	Rbm4	-1.98	5.5E-03	2.8E-03	RNA-binding protein 4
Q8BTR5	Dusp28	-0.66	6.0E-03	3.1E-03	Dual specificity phosphatase 28
P60710	Actb	-0.67	6.5E-03	3.3E-03	Actin, cytoplasmic 1
Q6P8J2	Sat2	0.83	6.5E-03	3.3E-03	Diamine acetyltransferase 2
Q9R1Q7	Plp2	0.97	6.8E-03	3.4E-03	Proteolipid protein 2
Q921H9	Coa7	-0.69	7.0E-03	3.5E-03	Cytochrome c oxidase assembly factor 7
Q9Z0F7	Sncg	-0.63	7.1E-03	3.6E-03	Gamma-synuclein
Q9D273	Mmab	-0.71	7.3E-03	3.7E-03	Corrinoid adenosyltransferase
Q8BIF0	Cd99l2	-0.61	7.4E-03	3.7E-03	CD99 antigen-like protein 2
O89116	Vti1a	-0.82	7.5E-03	3.8E-03	Vesicle transport through interaction with t-SNAREs homolog 1A
O70200	Aif1	-0.61	7.5E-03	3.8E-03	Allograft inflammatory factor 1
Q8BK30	Ndufv3	-0.86	7.8E-03	3.9E-03	NADH dehydrogenase [ubiquinone] flavoprotein 3, mitochondrial
P16879	Fes	0.68	8.0E-03	4.0E-03	Tyrosine-protein kinase Fes/Fps
Q9D7S9	Chmp5	-0.63	8.0E-03	4.0E-03	Charged multivesicular body protein 5
Q9WTZ8	Bex2	-0.82	8.3E-03	4.1E-03	Protein BEX2
Q9JMG7	Hdgfl3	-0.68	8.4E-03	4.2E-03	Hepatoma-derived growth factor-related protein 3
P16951	Atf2	-0.65	8.7E-03	4.3E-03	Cyclic AMP-dependent transcription factor ATF-2
Q8K070	Samd14	-0.83	8.9E-03	4.4E-03	Sterile alpha motif domain-containing protein 14
Q9WU63	Hebp2	-0.73	9.2E-03	4.5E-03	Heme-binding protein 2
P46414	Cdkn1b	-0.64	9.2E-03	4.5E-03	Cyclin-dependent kinase inhibitor 1B
P15066	Jund	-0.74	1.0E-02	4.9E-03	Transcription factor jun-D
Q9D826	Pipox	0.97	1.0E-02	4.9E-03	Peroxisomal sarcosine oxidase
Q8BXQ8	Fam53c	-0.63	1.0E-02	5.1E-03	Protein FAM53C
Q8VEE0	Rpe	-0.73	1.1E-02	5.1E-03	Ribulose-phosphate 3-epimerase
O08740	Polr2j	-0.62	1.1E-02	5.3E-03	DNA-directed RNA polymerase II subunit RPB11
Q8VCG9	Rfxap	-0.63	1.1E-02	5.4E-03	Regulatory factor X-associated protein
Q9CQW0	Emc6	0.56	1.1E-02	5.5E-03	ER membrane protein complex subunit 6
P08207	S100a10	-0.62	1.1E-02	5.5E-03	Protein S100-A10
Q9CZC8	Scrn1	-0.64	1.2E-02	5.6E-03	Secernin-1
Q8K1D8	Enho	-0.71	1.2E-02	5.6E-03	Adropin

Protein Accessions	Gene_name	log2(FC)	p-value	q-value	Protein Description
Q9JJW6	Alyref2	-0.65	1.3E-02	6.1E-03	Aly/REF export factor 2
Q9D3L0	Fam174a	-0.71	1.4E-02	6.4E-03	Membrane protein FAM174A
Q9D6J5	Ndufb8	-0.61	1.5E-02	6.8E-03	NADH dehydrogenase [ubiquinone] 1 beta subcomplex subunit 8, mitochondrial
Q61206	Pafah1b2	-0.72	1.5E-02	6.9E-03	Platelet-activating factor acetylhydrolase IB subunit beta
Q62190	Mst1r	-3.17	1.5E-02	6.9E-03	Macrophage-stimulating protein receptor
Q9D0V7	Ebag9	-0.76	1.5E-02	7.2E-03	Receptor-binding cancer antigen expressed on SiSo cells
Q91Y20	Pcdha10	-0.65	1.6E-02	7.2E-03	Protocadherin alpha-10
P70181	Pip5k1b	0.59	1.6E-02	7.4E-03	Phosphatidylinositol 4-phosphate 5-kinase type-1 beta
P11087	Col1a1	-0.75	1.6E-02	7.4E-03	Collagen alpha-1(I) chain
P32507	Nectin2	-0.68	1.6E-02	7.5E-03	Nectin-2
P10404	NaN	-0.65	1.7E-02	7.7E-03	MLV-related proviral Env polyprotein
Q9D110	Mthfs	1.01	1.7E-02	7.7E-03	5-formyltetrahydrofolate cyclo-ligase
Q80Y55	Bsdc1	-0.70	1.7E-02	7.8E-03	BSD domain-containing protein 1
Q9CR83	Rbm18	0.54	1.7E-02	7.8E-03	Probable RNA-binding protein 18
Q9CYA6	Zcchc8	-0.71	1.8E-02	8.1E-03	Zinc finger CCHC domain-containing protein 8
O70494	Sp3	-1.02	1.8E-02	8.1E-03	Transcription factor Sp3
Q5SUF2	Luc7l3	-0.64	1.8E-02	8.2E-03	Luc7-like protein 3
Q80WJ7	Mtdh	-0.65	1.8E-02	8.2E-03	Protein LYRIC
Q9ERD8	Parvg	-0.90	1.9E-02	8.5E-03	Gamma-parvin
Q9D735	Trir	-0.66	1.9E-02	8.7E-03	Telomerase RNA component interacting RNase
P70339;Q8K025	Frat1;Frat2	-0.78	2.0E-02	9.1E-03	Proto-oncogene FRAT1;GSK-3-binding protein FRAT2
Q8BXL9	Iffo1	-0.79	2.1E-02	9.5E-03	Intermediate filament family orphan 1
Q80UU9	Pgrmc2	-0.73	2.1E-02	9.5E-03	Membrane-associated progesterone receptor component 2
O08997	Atox1	-0.61	2.1E-02	9.6E-03	Copper transport protein ATOX1
Q9DB91	Med29	-0.73	2.2E-02	9.6E-03	Mediator of RNA polymerase II transcription subunit 29
P0CL69	Znf703	-1.30	2.2E-02	9.7E-03	Zinc finger protein 703
Q8VE99	Ccdc115	-0.61	2.2E-02	9.7E-03	Coiled-coil domain-containing protein 115
Q8R0L9	Tada3	-0.62	2.2E-02	9.8E-03	Transcriptional adapter 3
Q9CXU9	Eif1b	-0.76	2.2E-02	9.9E-03	Eukaryotic translation initiation factor 1b
Q9JL19	Ncoa6	-0.60	2.3E-02	1.0E-02	Nuclear receptor coactivator 6
Q91XZ4	Pcdhb6	-0.62	2.4E-02	1.0E-02	Protocadherin beta-6
Q8BW22	Ss18l1	-0.73	2.4E-02	1.0E-02	Calcium-responsive transactivator
Q9D7M8	Polr2d	-0.64	2.4E-02	1.1E-02	DNA-directed RNA polymerase II subunit RPB4
Q8R1N0	Znf830	-0.72	2.4E-02	1.1E-02	Zinc finger protein 830
Q14AM7	NaN	-0.63	2.5E-02	1.1E-02	UPF0472 protein C16orf72 homolog
O35491	Clk2	0.57	2.5E-02	1.1E-02	Dual specificity protein kinase CLK2

Protein Accessions	Gene_name	log2(FC)	p-value	q-value	Protein Description
Q8K194	Snrnp27	-0.66	2.6E-02	1.1E-02	U4/U6.U5 small nuclear ribonucleoprotein 27 kDa protein
P18572	Bsg	-0.62	2.6E-02	1.1E-02	Basigin
Q8BHE0	Prr11	0.58	2.7E-02	1.2E-02	Proline-rich protein 11
Q8BU11	Tox4	-0.61	2.7E-02	1.2E-02	TOX high mobility group box family member 4
Q05722	Col9a1	-0.72	2.7E-02	1.2E-02	Collagen alpha-1(IX) chain
Q63850	Nup62	-0.64	2.8E-02	1.2E-02	Nuclear pore glycoprotein p62
Q91Z38	Ttc1	-0.78	2.8E-02	1.2E-02	Tetratricopeptide repeat protein 1
Q9QXV3	Ing1	-0.75	2.9E-02	1.2E-02	Inhibitor of growth protein 1
Q62481	Vps72	-0.75	2.9E-02	1.2E-02	Vacuolar protein sorting-associated protein 72 homolog
Q9DAT2	Mrgbp	-0.74	2.9E-02	1.2E-02	MRG/MORF4L-binding protein
Q80YC5	F12	-0.68	2.9E-02	1.2E-02	Coagulation factor XII
E9PV86	Mctp1	-1.40	2.9E-02	1.2E-02	Multiple C2 and transmembrane domain-containing protein 1
Q99LQ4	Svbp	-0.73	2.9E-02	1.2E-02	Small vasohibin-binding protein
Q8VEK2	Rhbdd2	0.71	2.9E-02	1.2E-02	Rhomboid domain-containing protein 2
Q8CHH5	Bicral	-0.65	3.0E-02	1.3E-02	BRD4-interacting chromatin-remodeling complex-associated protein-like
Q9WTQ5	Akap12	-0.62	3.0E-02	1.3E-02	A-kinase anchor protein 12
Q9CZE3	Rab32	-0.80	3.1E-02	1.3E-02	Ras-related protein Rab-32
Q78ZA7	Nap1l4	-0.61	3.2E-02	1.3E-02	Nucleosome assembly protein 1-like 4
Q01147	Creb1	-1.00	3.2E-02	1.3E-02	Cyclic AMP-responsive element-binding protein 1
Q9JL35	Hmgn5	-0.62	3.2E-02	1.4E-02	High mobility group nucleosome-binding domain-containing protein 5
Q60772	Cdkn2c	-0.74	3.3E-02	1.4E-02	Cyclin-dependent kinase 4 inhibitor C
Q9CQM2	Kdelr2	0.89	3.3E-02	1.4E-02	ER lumen protein-retaining receptor 2
Q9WU01	Khdrbs2	-0.69	3.5E-02	1.5E-02	KH domain-containing, RNA-binding, signal transduction-associated protein 2
Q9D8C6	Med11	-0.62	3.5E-02	1.5E-02	Mediator of RNA polymerase II transcription subunit 11
Q9CWZ3	Rbm8a	-0.66	3.6E-02	1.5E-02	RNA-binding protein 8A
Q80XU3	Nucks1	-0.65	3.6E-02	1.5E-02	Nuclear ubiquitous casein and cyclin-dependent kinase substrate 1
Q5SV06	Spata22	0.91	3.7E-02	1.5E-02	Spermatogenesis-associated protein 22
Q61074	Ppm1g	-0.61	3.7E-02	1.5E-02	Protein phosphatase 1G
Q9D164	Fxyd6	-0.60	3.8E-02	1.6E-02	FXYD domain-containing ion transport regulator 6
Q62241	Snrpc	-0.61	3.8E-02	1.6E-02	U1 small nuclear ribonucleoprotein C
Q8VE92	Rbm4b	-0.80	3.8E-02	1.6E-02	RNA-binding protein 4B
Q05AH6	Spindoc	-0.62	4.0E-02	1.6E-02	Spindlin interactor and repressor of chromatin-binding protein
Q9D287	Bcas2	-0.60	4.0E-02	1.6E-02	Pre-mRNA-splicing factor SPF27
P63147	Ube2b	-0.67	4.3E-02	1.8E-02	Ubiquitin-conjugating enzyme E2 B
Q8BP92	Rcn2	-0.73	4.3E-02	1.8E-02	Reticulocalbin-2
Q61502	E2f5	-0.62	4.4E-02	1.8E-02	Transcription factor E2F5

Protein Accessions	Gene_name	log2(FC)	p-value	q-value	Protein Description
Q91W82	Ube2e2	-0.76	4.5E-02	1.8E-02	Ubiquitin-conjugating enzyme E2 E2
Q8K5B2	Mcfd2	-0.79	4.5E-02	1.8E-02	Multiple coagulation factor deficiency protein 2 homolog
Q9CR02	Tma16	-0.71	4.8E-02	1.9E-02	Translation machinery-associated protein 16
Q9WU00	Nrf1	-0.64	4.8E-02	1.9E-02	Nuclear respiratory factor 1
Q61146	Ocln	-0.60	4.8E-02	1.9E-02	Occludin
O88665	Brd7	-0.61	4.8E-02	1.9E-02	Bromodomain-containing protein 7
Q8VDY9	Caap1	-0.60	4.8E-02	1.9E-02	Caspase activity and apoptosis inhibitor 1
Q62093	Srsf2	-0.62	4.9E-02	1.9E-02	Serine/arginine-rich splicing factor 2
Q00420	Gabpb1	-0.60	5.1E-02	2.0E-02	GA-binding protein subunit beta-1
Q61189	Clns1a	-0.60	5.1E-02	2.0E-02	Methylosome subunit pICln
Q8C1M2	Znf428	-1.12	5.1E-02	2.0E-02	Zinc finger protein 428
O55042	Snca	-0.68	5.2E-02	2.0E-02	Alpha-synuclein
Q9DAY5	NaN	-0.61	5.3E-02	2.1E-02	UPF0669 protein C6orf120 homolog
Q8K1L2	Spin4	0.78	5.3E-02	2.1E-02	Spindlin-4
Q8VCE4	NaN	-0.73	5.4E-02	2.1E-02	Uncharacterized protein C9orf40 homolog
Q8BRH3	Arhgap19	-0.74	5.4E-02	2.1E-02	Rho GTPase-activating protein 19
P27661	H2ax	0.64	5.6E-02	2.2E-02	Histone H2AX
Q99JP4	Cdc26	-0.69	5.7E-02	2.2E-02	Anaphase-promoting complex subunit CDC26
P70670	Naca	0.67	5.8E-02	2.2E-02	Nascent polypeptide-associated complex subunit alpha, muscle-specific form
Q60973	Rbbp7	-0.62	5.8E-02	2.3E-02	Histone-binding protein RBBP7
P70274	Selenop	-0.66	5.9E-02	2.3E-02	Selenoprotein P
Q60972	Rbbp4	-0.94	5.9E-02	2.3E-02	Histone-binding protein RBBP4
Q61205	Pafah1b3	-0.61	5.9E-02	2.3E-02	Platelet-activating factor acetylhydrolase IB subunit gamma
Q8R3E3	Wipi1	-0.97	6.2E-02	2.4E-02	WD repeat domain phosphoinositide-interacting protein 1
Q3TAS6	Emc10	-0.67	6.2E-02	2.4E-02	ER membrane protein complex subunit 10
Q9D6V8	Paip2	-0.70	6.4E-02	2.5E-02	Polyadenylate-binding protein-interacting protein 2
Q9CWK3	Cd2bp2	-0.64	7.1E-02	2.7E-02	CD2 antigen cytoplasmic tail-binding protein 2
Q7TQI8	Tspyl2	-1.05	7.2E-02	2.7E-02	Testis-specific Y-encoded-like protein 2
Q9EQU5	Set	-0.62	7.8E-02	2.9E-02	Protein SET
Q8VE98	Cd276	-0.78	8.1E-02	3.0E-02	CD276 antigen
P97825	Jpt1	-0.69	8.2E-02	3.1E-02	Jupiter microtubule associated homolog 1
Q0VBL1	Tigd2	-0.75	8.7E-02	3.2E-02	Tigger transposable element-derived protein 2
Q3TXT3	Inip	-0.63	9.0E-02	3.3E-02	SOSS complex subunit C
Q8BWH0	Slc38a7	0.81	9.9E-02	3.6E-02	Putative sodium-coupled neutral amino acid transporter 7
P55821	Stmn2	-0.63	1.1E-01	3.8E-02	Stathmin-2
Q91ZF0	Dnajc24	-0.62	1.1E-01	3.9E-02	DnaJ homolog subfamily C member 24

Protein Accessions	Gene_name	log2(FC)	p-value	q-value	Protein Description
Q9Z180	Setbp1	-0.68	1.1E-01	3.9E-02	SET-binding protein
Q8BGH7	Cdc42se2	-0.69	1.1E-01	4.0E-02	CDC42 small effector protein 2
Q99LW6	Yaf2	-0.82	1.2E-01	4.2E-02	YY1-associated factor 2
Q9DCB1	Hmgn3	-0.84	1.2E-01	4.2E-02	High mobility group nucleosome-binding domain-containing protein 3
Q99KG7	Hps4	-0.67	1.2E-01	4.3E-02	Hermansky-Pudlak syndrome 4 protein homolog
Q8CI85	Ca12	0.75	1.3E-01	4.5E-02	Carbonic anhydrase 12
Q7TPD7	Lrrc75b	-0.63	1.4E-01	4.9E-02	Leucine-rich repeat-containing protein 75B
P51675	Ccr1	-0.61	1.6E-01	5.3E-02	C-C chemokine receptor type 1
Q80V24	Vgll4	-0.69	1.6E-01	5.4E-02	Transcription cofactor vestigial-like protein 4
Q7TN60	Tmc6	0.61	1.7E-01	5.6E-02	Transmembrane channel-like protein 6
P28667	Marcksl1	-0.92	1.7E-01	5.7E-02	MARCKS-related protein
Q922U2	Krt5	0.62	1.8E-01	6.0E-02	Keratin, type II cytoskeletal 5
Q5I1X5	Ppp1r13l	-0.68	1.9E-01	6.2E-02	RelA-associated inhibitor
Q9D945	Llph	0.69	2.1E-01	6.8E-02	Protein LLP homolog
Q9ES18	Foxj2	-0.73	2.1E-01	6.9E-02	Forkhead box protein J2
Q9D593	Atp6v1e2	0.56	2.2E-01	7.1E-02	V-type proton ATPase subunit E 2
Q9D032	Ssbp3	-0.65	2.2E-01	7.1E-02	Single-stranded DNA-binding protein 3
P0C8B4	Gon7	-0.74	2.4E-01	7.7E-02	EKC/KEOPS complex subunit GON7
Q03391	Grin2d	0.78	2.5E-01	8.0E-02	Glutamate receptor ionotropic, NMDA 2D
Q8VED5	Krt79	0.69	2.9E-01	9.1E-02	Keratin, type II cytoskeletal 79
Q920F6	Smc1b	1.30	3.1E-01	9.6E-02	Structural maintenance of chromosomes protein 1B
Q61781	Krt14	-1.11	3.8E-01	1.1E-01	Keratin, type I cytoskeletal 14
Q80UZ0	Fgd5	-1.51	4.1E-01	1.2E-01	FYVE, RhoGEF and PH domain-containing protein 5
Q6DIA2	Exoc3l4	0.65	4.6E-01	1.3E-01	Exocyst complex component 3-like protein 4
P46425	Gstp2	-1.15	4.9E-01	1.4E-01	Glutathione S-transferase P 2
Q91ZE5	Adgre4	-0.61	5.0E-01	1.4E-01	Adhesion G protein-coupled receptor E4
Q6IFZ9	Krt74	1.44	5.1E-01	1.5E-01	Keratin, type II cytoskeletal 74
Q8R4U0	Stab2	0.65	5.5E-01	1.6E-01	Stabilin-2
Q9WUH1	Tmem115	0.56	7.2E-01	1.9E-01	Transmembrane protein 115
P61327	Magoh	-0.77	7.4E-01	2.0E-01	Protein mago nashi homolog
P43135	Nr2f2	-0.61	7.8E-01	2.1E-01	COUP transcription factor 2
P08730	Krt13	0.65	9.9E-01	2.5E-01	Keratin, type I cytoskeletal 13

CB Proteome 6 weeks 0.1Gy vs Sham

Protein Accessions	Gene_name	log2(FC)	p-value	q-value	Protein Description
O88809	Dcx	1.27	8.0E-09	2.8E-08	Neuronal migration protein doublecortin
Q9CPP0	Npm3	0.71	4.7E-06	5.7E-06	Nucleoplasmin-3
Q9DBW3	Natd1	0.55	4.8E-06	5.7E-06	Protein NATD1
Q61133	Gstt2	0.65	1.3E-05	1.4E-05	Glutathione S-transferase theta-2
Q8R4P9	Abcc10	0.88	2.6E-05	2.6E-05	Multidrug resistance-associated protein 7
Q9R0P4	Smap	0.55	3.3E-05	3.1E-05	Small acidic protein
Q8CG48	Smc2	0.77	5.4E-05	4.7E-05	Structural maintenance of chromosomes protein 2
P97310	Mcm2	1.12	9.8E-05	8.1E-05	DNA replication licensing factor MCM2
Q8K2Z4	Ncapd2	0.70	1.1E-04	8.8E-05	Condensin complex subunit 1
Q8VE92	Rbm4b	-0.68	1.2E-04	9.8E-05	RNA-binding protein 4B
Q61881	Mcm7	0.80	1.6E-04	1.2E-04	DNA replication licensing factor MCM7
P33610	Prim2	0.91	2.0E-04	1.5E-04	DNA primase large subunit
P49717	Mcm4	0.59	2.1E-04	1.6E-04	DNA replication licensing factor MCM4
Q8VC69	Slc22a6	0.56	2.9E-04	2.1E-04	Solute carrier family 22 member 6
P97311	Mcm6	1.50	3.4E-04	2.4E-04	DNA replication licensing factor MCM6
Q00519	Xdh	0.79	4.3E-04	3.0E-04	Xanthine dehydrogenase/oxidase
P37913	Lig1	0.65	4.9E-04	3.3E-04	DNA ligase 1
Q9D826	Pipox	0.62	4.9E-04	3.4E-04	Peroxisomal sarcosine oxidase
P11440	Cdk1	0.68	5.8E-04	3.9E-04	Cyclin-dependent kinase 1
Q80WC1	Ubn2	0.61	5.9E-04	4.0E-04	Ubinuclein-2
Q61704	Itih3	0.58	7.0E-04	4.6E-04	Inter-alpha-trypsin inhibitor heavy chain H3
Q3UZW7	Eef2kmt	0.71	7.8E-04	5.0E-04	Protein-lysine N-methyltransferase EEF2KMT
Q7TNS2	Micos10	-0.75	7.8E-04	5.1E-04	MICOS complex subunit Mic10
Q9R0H0	Acox1	0.66	8.0E-04	5.1E-04	Peroxisomal acyl-coenzyme A oxidase 1
Q8C4U3	Sfrp1	1.36	1.5E-03	8.7E-04	Secreted frizzled-related protein 1
P47880	Igfbp6	0.61	1.9E-03	1.1E-03	Insulin-like growth factor-binding protein 6
Q80UU9	Pgrmc2	-0.72	2.1E-03	1.2E-03	Membrane-associated progesterone receptor component 2
Q64471	Gstt1	-0.63	2.3E-03	1.3E-03	Glutathione S-transferase theta-1
P61148	Fgf1	0.58	2.3E-03	1.3E-03	Fibroblast growth factor 1
Q8K0W9	Dph3	-0.62	2.5E-03	1.4E-03	DPH3 homolog
Q9D174	Ss18l2	0.58	5.4E-03	2.8E-03	SS18-like protein 2
O35071	Kif1c	-2.20	5.6E-03	2.9E-03	Kinesin-like protein KIF1C
P50153	Gng4	-0.88	8.8E-03	4.3E-03	Guanine nucleotide-binding protein G(I)/G(S)/G(O) subunit gamma-4
Q61188	Ezh2	0.66	9.8E-03	4.8E-03	Histone-lysine N-methyltransferase EZH2
Q8R1H0	Hopx	-0.63	1.1E-02	5.1E-03	Homeodomain-only protein

Protein Accessions	Gene_name	log2(FC)	p-value	q-value	Protein Description
Q6PFH3	Dcaf15	0.63	1.1E-02	5.2E-03	DDB1- and CUL4-associated factor 15
P62322	Lsm5	-0.69	1.1E-02	5.5E-03	U6 snRNA-associated Sm-like protein LSm5
P16110	Lgals3	0.67	1.2E-02	5.8E-03	Galectin-3
Q9JLY0	Socs6	-1.04	1.7E-02	7.7E-03	Suppressor of cytokine signaling 6
E9Q2Z1	Cecr2	0.78	1.9E-02	8.5E-03	Cat eye syndrome critical region protein 2 homolog
Q8BGD6	Slc38a9	-0.61	2.0E-02	8.8E-03	Sodium-coupled neutral amino acid transporter 9
P47212	Gal	-0.67	2.7E-02	1.2E-02	Galanin peptides
Q673H1	Tusc1	1.01	3.5E-02	1.4E-02	Tumor suppressor candidate gene 1 protein homolog
Q8C4U2	Tmem145	-0.66	3.9E-02	1.6E-02	Transmembrane protein 145
Q62230	Siglec1	0.63	4.1E-02	1.7E-02	Sialoadhesin
P04247	Mb	-0.97	5.0E-02	2.0E-02	Myoglobin
Q3USH1	Insyn2a	-0.60	5.3E-02	2.1E-02	Inhibitory synaptic factor 2A
P15331	Prph	-1.44	5.8E-02	2.3E-02	Peripherin
Q60771	Cldn11	-0.64	6.1E-02	2.4E-02	Claudin-11
P61588	Rnd3	1.44	6.5E-02	2.5E-02	Rho-related GTP-binding protein RhoE
Q8CGV2	Tph2	-0.68	6.6E-02	2.5E-02	Tryptophan 5-hydroxylase 2
Q3U1Z5	Gpsm3	0.61	7.1E-02	2.7E-02	G-protein-signaling modulator 3
O08832	Galnt4	0.61	7.9E-02	3.0E-02	Polypeptide N-acetylgalactosaminyltransferase 4
P06880	Gh1	4.56	8.4E-02	3.1E-02	Somatotropin
Q64261	Cdk6	1.05	8.7E-02	3.2E-02	Cyclin-dependent kinase 6
Q61696	Hspa1a	-0.61	8.9E-02	3.3E-02	Heat shock 70 kDa protein 1A
Q62313	Tgoln1	-0.81	1.0E-01	3.7E-02	Trans-Golgi network integral membrane protein 1
Q761V0	Slc6a5	-0.79	1.1E-01	3.8E-02	Sodium- and chloride-dependent glycine transporter 2
Q14B80	Kcnc2	-0.71	1.1E-01	3.8E-02	Potassium voltage-gated channel subfamily C member 2
P07091	S100a4	0.64	1.1E-01	4.0E-02	Protein S100-A4
Q61762	Kcna5	-0.61	1.2E-01	4.2E-02	Potassium voltage-gated channel subfamily A member 5
Q8VE98	Cd276	0.57	1.4E-01	4.8E-02	CD276 antigen
P27005	S100a8	0.96	1.4E-01	4.9E-02	Protein S100-A8
P52019	Sqle	1.10	1.6E-01	5.4E-02	Squalene monooxygenase
Q5GH67	Xkr4	0.98	1.6E-01	5.4E-02	XK-related protein 4
Q62283	Tspan7	-1.25	1.7E-01	5.6E-02	Tetraspanin-7
Q8BYW1	Arhgap25	0.62	1.7E-01	5.8E-02	Rho GTPase-activating protein 25
P70377	Fgf13	1.15	1.8E-01	5.9E-02	Fibroblast growth factor 13
O09053	Wrn	-0.73	2.1E-01	6.8E-02	Werner syndrome ATP-dependent helicase homolog
Q9DCB1	Hmgn3	0.83	2.1E-01	6.8E-02	High mobility group nucleosome-binding domain-containing protein 3
Q62190	Mst1r	0.74	2.2E-01	7.2E-02	Macrophage-stimulating protein receptor

Protein Accessions	Gene_name	log2(FC)	p-value	q-value	Protein Description
P31725	S100a9	0.79	2.2E-01	7.2E-02	Protein S100-A9
O70176	Adcyap1	-0.73	2.2E-01	7.2E-02	Pituitary adenylate cyclase-activating polypeptide
Q69ZT1	Fan1	0.60	2.5E-01	7.8E-02	Fanconi-associated nuclease 1
P08905	Lyz2	1.83	2.8E-01	8.7E-02	Lysozyme C-2
O35744	Chil3	1.59	2.9E-01	9.0E-02	Chitinase-like protein 3
[illegible];B5X0G2;P02762;P04938;	[illegible]18;Mup17;Mup6;Mup11;M[illegible]	0.73	3.1E-01	9.6E-02	Major urinary protein 18; 17; 6; 11; 1
Q9D8T4	Tvp23b	0.67	3.2E-01	1.0E-01	Golgi apparatus membrane protein TVP23 homolog B
C6KI89	Catsperg2	0.76	3.4E-01	1.0E-01	Cation channel sperm-associated protein subunit gamma 2
Q9D593	Atp6v1e2	0.62	4.1E-01	1.2E-01	V-type proton ATPase subunit E 2
Q8C796	Rcor2	1.30	4.2E-01	1.2E-01	REST corepressor 2
P70670	Naca	1.52	4.8E-01	1.4E-01	Nascent polypeptide-associated complex subunit alpha, muscle-specific form

CB Proteome 6 weeks 2Gy vs Sham

Protein Accessions	Gene_name	log2(FC)	p-value	q-value	Protein Description
Q6ZWY8	Tmsb10	0.57	1.4E-11	2.6E-10	Thymosin beta-10
Q8R4S0	Ppp1r14c	0.68	2.8E-11	4.2E-10	Protein phosphatase 1 regulatory subunit 14C
O88809	Dcx	3.22	6.7E-11	7.8E-10	Neuronal migration protein doublecortin
Q9JIP4	Panx1	0.69	7.3E-10	4.5E-09	Pannexin-1
Q80WC1	Ubn2	0.67	3.9E-09	1.6E-08	Ubinuclein-2
P97310	Mcm2	1.94	5.6E-09	2.1E-08	DNA replication licensing factor MCM2
Q8CG48	Smc2	0.92	9.9E-09	3.4E-08	Structural maintenance of chromosomes protein 2
Q921H8	Acaa1a	-0.65	1.3E-08	4.2E-08	3-ketoacyl-CoA thiolase A, peroxisomal
P49717	Mcm4	1.09	1.6E-08	4.9E-08	DNA replication licensing factor MCM4
Q05186	Rcn1	0.54	2.7E-08	7.4E-08	Reticulocalbin-1
Q3UFB2	Znhit6	0.67	7.0E-08	1.6E-07	Box C/D snoRNA protein 1
Q9CPP0	Npm3	1.45	9.1E-08	2.0E-07	Nucleoplasmin-3
P70211	Dcc	0.85	1.1E-07	2.3E-07	Netrin receptor DCC
Q9JIM1	Slc29a1	0.86	1.2E-07	2.6E-07	Equilibrative nucleoside transporter 1
Q61881	Mcm7	1.93	1.3E-07	2.7E-07	DNA replication licensing factor MCM7
P49718	Mcm5	0.89	1.3E-07	2.7E-07	DNA replication licensing factor MCM5
Q8BY71	Hat1	0.55	1.4E-07	2.9E-07	Histone acetyltransferase type B catalytic subunit
Q62084	Ppp1r14b	0.80	2.1E-07	4.0E-07	Protein phosphatase 1 regulatory subunit 14B
P17918	Pcna	1.11	2.2E-07	4.2E-07	Proliferating cell nuclear antigen
P97311	Mcm6	2.45	2.4E-07	4.5E-07	DNA replication licensing factor MCM6
E9Q2Z1	Cecr2	1.10	2.9E-07	5.3E-07	Cat eye syndrome critical region protein 2 homolog
P33610	Prim2	1.48	3.1E-07	5.5E-07	DNA primase large subunit

Protein Accessions	Gene_name	log2(FC)	p-value	q-value	Protein Description
Q9CXK8	Nip7	0.55	3.4E-07	6.1E-07	60S ribosome subunit biogenesis protein NIP7 homolog
P97792	Cxadr	0.60	3.5E-07	6.3E-07	Coxsackievirus and adenovirus receptor homolog
P11609	Cd1d1	1.04	3.6E-07	6.3E-07	Antigen-presenting glycoprotein CD1d1
P97496	Smarcc1	0.77	4.7E-07	7.9E-07	SWI/SNF complex subunit SMARCC1
Q80YS6	Afap1	0.61	5.1E-07	8.4E-07	Actin filament-associated protein 1
P11440	Cdk1	0.70	7.4E-07	1.2E-06	Cyclin-dependent kinase 1
Q80TS5	Znf423	0.56	7.8E-07	1.2E-06	Zinc finger protein 423
Q60772	Cdkn2c	0.68	7.9E-07	1.2E-06	Cyclin-dependent kinase 4 inhibitor C
P37913	Lig1	1.25	9.1E-07	1.4E-06	DNA ligase 1
P26350	Ptma	0.70	9.5E-07	1.4E-06	Prothymosin alpha
Q7TPB0	Plppr3	0.65	1.2E-06	1.7E-06	Phospholipid phosphatase-related protein type 3
Q8C4U3	Sfrp1	2.06	1.4E-06	2.0E-06	Secreted frizzled-related protein 1
Q8VE10	Naa40	1.07	1.4E-06	2.0E-06	N-alpha-acetyltransferase 40
Q6P5G3	Mbtd1	0.77	1.7E-06	2.3E-06	MBT domain-containing protein 1
O35464	Sema6a	0.56	1.8E-06	2.5E-06	Semaphorin-6A
P07742	Rrm1	0.66	2.0E-06	2.7E-06	Ribonucleoside-diphosphate reductase large subunit
P12025	Mdk	0.62	2.0E-06	2.7E-06	Midkine
Q5RL79	Krtcap2	-0.64	2.7E-06	3.5E-06	Keratinocyte-associated protein 2
Q9WUK4	Rfc2	0.56	3.0E-06	3.8E-06	Replication factor C subunit 2
Q64261	Cdk6	1.00	3.2E-06	4.0E-06	Cyclin-dependent kinase 6
P30285	Cdk4	0.61	3.2E-06	4.1E-06	Cyclin-dependent kinase 4
P17225	Ptbp1	0.62	4.0E-06	4.9E-06	Polypyrimidine tract-binding protein 1
P20664	Prim1	0.79	4.4E-06	5.4E-06	DNA primase small subunit
Q6PFE7	Tmeff1	0.94	5.1E-06	6.1E-06	Tomoregulin-1
Q9CZG9	Pdzd11	-0.73	5.7E-06	6.7E-06	PDZ domain-containing protein 11
Q9CQ18	Rnaseh2c	0.58	6.0E-06	7.0E-06	Ribonuclease H2 subunit C
Q922W5	Pycr1	0.73	6.3E-06	7.3E-06	Pyrroline-5-carboxylate reductase 1, mitochondrial
Q8VE92	Rbm4b	-1.71	6.5E-06	7.5E-06	RNA-binding protein 4B
Q3UGP9	Lrrc58	0.72	1.1E-05	1.2E-05	Leucine-rich repeat-containing protein 58
Q80YP6	Zik1	1.21	1.2E-05	1.2E-05	Zinc finger protein interacting with ribonucleoprotein K
P01872	Ighm	-0.85	1.3E-05	1.3E-05	Immunoglobulin heavy constant mu
Q8K0W9	Dph3	-1.14	1.4E-05	1.4E-05	DPH3 homolog
Q0VBL1	Tigd2	-0.64	1.4E-05	1.5E-05	Tigger transposable element-derived protein 2
Q9D826	Pipox	0.57	1.4E-05	1.5E-05	Peroxisomal sarcosine oxidase
Q14DH7	Acss3	-0.92	1.7E-05	1.8E-05	Acyl-CoA synthetase short-chain family member 3, mitochondrial
Q8K2Z4	Ncapd2	0.73	3.8E-05	3.5E-05	Condensin complex subunit 1

Protein Accessions	Gene_name	log2(FC)	p-value	q-value	Protein Description
Q9D0F6	Rfc5	0.76	4.4E-05	4.0E-05	Replication factor C subunit 5
Q7TNS2	Micos10	-1.31	4.6E-05	4.1E-05	MICOS complex subunit Mic10
Q921J4	Ube2s	0.59	5.7E-05	5.0E-05	Ubiquitin-conjugating enzyme E2 S
P63054	Pcp4	-1.13	8.4E-05	7.0E-05	Calmodulin regulator protein PCP4
KE6;C0HKE7;C0HKE8;C0H	;Hist1h2an;Hist1h2ao;Hist	0.56	9.0E-05	7.5E-05	Histone H2A type 1-B; 1-C; 1-D; 1-E; 1-G; 1-I; 1-N; 1-O; 1-P; type 3; 1-F; 1-H; 1-K; Histone H2A,J
Q61133	Gstt2	0.58	1.0E-04	8.4E-05	Glutathione S-transferase theta-2
O08832	Galnt4	0.74	1.1E-04	8.8E-05	Polypeptide N-acetylgalactosaminyltransferase 4
Q9QY93	Dctpp1	0.54	1.1E-04	9.3E-05	dCTP pyrophosphatase 1
Q52KG4	Zbtb45	0.71	1.2E-04	9.4E-05	Zinc finger and BTB domain-containing protein 45
P80206	Otx2	0.84	1.5E-04	1.2E-04	Homeobox protein OTX2
P84228	H3c2	-0.65	1.8E-04	1.4E-04	Histone H3,2
Q9DCF9	Ssr3	-0.64	1.9E-04	1.5E-04	Translocon-associated protein subunit gamma
P47880	Igfbp6	0.61	2.4E-04	1.7E-04	Insulin-like growth factor-binding protein 6
Q64471	Gstt1	-0.83	2.5E-04	1.8E-04	Glutathione S-transferase theta-1
P48301	Tead2	-0.75	2.6E-04	1.9E-04	Transcriptional enhancer factor TEF-4
Q9R2B6	St6galnac4	-0.69	2.6E-04	1.9E-04	Alpha-N-acetyl-neuraminyl-2,3-beta-galactosyl-1,3-N-acetyl-galactosaminide alpha-2,6-sialyltransferase
Q6P5U8	Ccdc148	-0.61	3.3E-04	2.4E-04	Coiled-coil domain-containing protein 148
Q62192	Cd180	0.56	3.6E-04	2.5E-04	CD180 antigen
Q9D174	Ss18l2	0.59	3.7E-04	2.6E-04	SS18-like protein 2
P03899	Mtnd3	-0.87	3.9E-04	2.7E-04	NADH-ubiquinone oxidoreductase chain 3
Q62230	Siglec1	0.67	4.1E-04	2.9E-04	Sialoadhesin
Q9DBW3	Natd1	0.70	5.6E-04	3.7E-04	Protein NATD1
P11157	Rrm2	-0.78	5.9E-04	3.9E-04	Ribonucleoside-diphosphate reductase subunit M2
Q9Z0J1	Reck	0.62	6.0E-04	4.0E-04	Reversion-inducing cysteine-rich protein with Kazal motifs
Q3UZW7	Eef2kmt	0.57	6.3E-04	4.1E-04	Protein-lysine N-methyltransferase EEF2KMT
P16110	Lgals3	0.59	6.3E-04	4.2E-04	Galectin-3
P70274	Selenop	-0.62	7.0E-04	4.6E-04	Selenoprotein P
P61588	Rnd3	1.49	8.9E-04	5.7E-04	Rho-related GTP-binding protein RhoE
Q9CYW4	Hdhd3	-0.61	9.5E-04	6.0E-04	Haloacid dehalogenase-like hydrolase domain-containing protein 3
Q9CQ85	Timm22	-0.61	1.0E-03	6.3E-04	Mitochondrial import inner membrane translocase subunit Tim22
Q8C4U2	Tmem145	-1.37	1.3E-03	7.9E-04	Transmembrane protein 145
Q8BN78	Zbtb33	0.57	1.5E-03	9.0E-04	Transcriptional regulator Kaiso
A1L314	Mpeg1	1.09	1.7E-03	1.0E-03	Macrophage-expressed gene 1 protein
Q8R4P9	Abcc10	0.70	1.7E-03	1.0E-03	Multidrug resistance-associated protein 7
Q9D0S9	Hint2	0.70	1.7E-03	1.0E-03	Histidine triad nucleotide-binding protein 2, mitochondrial
Q9R1Q7	Plp2	1.05	2.0E-03	1.2E-03	Proteolipid protein 2

Protein Accessions	Gene_name	log2(FC)	p-value	q-value	Protein Description
Q9D0V7	Ebag9	-0.63	2.5E-03	1.4E-03	Receptor-binding cancer antigen expressed on SiSo cells
Q8CGB6	Tns2	0.55	2.6E-03	1.5E-03	Tensin-2
Q6P8J7	Ckmt2	1.19	3.5E-03	1.9E-03	Creatine kinase S-type, mitochondrial
P31266	Rbpj	0.59	4.7E-03	2.5E-03	Recombining binding protein suppressor of hairless
Q62226	Shh	0.64	4.7E-03	2.5E-03	Sonic hedgehog protein
Q6PFH3	Dcaf15	0.56	4.8E-03	2.5E-03	DDB1- and CUL4-associated factor 15
Q8VHQ4	Rab40c	0.66	5.4E-03	2.8E-03	Ras-related protein Rab-40C
O35071	Kif1c	-2.15	5.8E-03	3.0E-03	Kinesin-like protein KIF1C
P46425	Gstp2	-0.90	7.0E-03	3.6E-03	Glutathione S-transferase P 2
Q9D593	Atp6v1e2	0.99	7.1E-03	3.6E-03	V-type proton ATPase subunit E 2
Q8VCE2	Gpn1	0.56	7.2E-03	3.6E-03	GPN-loop GTPase 1
Q19LI2	A1bg	-1.13	7.7E-03	3.8E-03	Alpha-1B-glycoprotein
Q62407	Speg	-0.60	8.4E-03	4.2E-03	Striated muscle-specific serine/threonine-protein kinase
O54992	Mapkapk5	0.62	8.8E-03	4.4E-03	MAP kinase-activated protein kinase 5
Q8R527	Rhoq	-0.75	1.1E-02	5.4E-03	Rho-related GTP-binding protein RhoQ
Q9Z2E2	Mbd1	0.56	1.1E-02	5.5E-03	Methyl-CpG-binding domain protein 1
Q6DFX2	Antxr2	0.55	1.2E-02	5.7E-03	Anthrax toxin receptor 2
Q9JLY0	Socs6	-1.62	1.2E-02	5.8E-03	Suppressor of cytokine signaling 6
E9Q4F2	Klhl18	0.61	1.5E-02	6.8E-03	Kelch-like protein 18
Q99LX5	Mmtag2	0.61	1.9E-02	8.7E-03	Multiple myeloma tumor-associated protein 2 homolog
P61205	Arf3	-0.70	2.0E-02	8.9E-03	ADP-ribosylation factor 3
Q60980	Klf3	-0.62	2.1E-02	9.3E-03	Krueppel-like factor 3
Q8BYW1	Arhgap25	0.76	2.2E-02	9.6E-03	Rho GTPase-activating protein 25
O35744	Chil3	0.81	2.5E-02	1.1E-02	Chitinase-like protein 3
Q62313	Tgoln1	-1.22	2.9E-02	1.2E-02	Trans-Golgi network integral membrane protein 1
Q922U2	Krt5	-0.66	4.1E-02	1.7E-02	Keratin, type II cytoskeletal 5
Q8BLB7	L3mbtl3	0.63	4.2E-02	1.7E-02	Lethal(3)malignant brain tumor-like protein 3
P08905	Lyz2	1.24	4.6E-02	1.8E-02	Lysozyme C-2
Q8BKT8	Haus7	0.70	4.9E-02	1.9E-02	HAUS augmin-like complex subunit 7
O54983	Crym	0.69	5.0E-02	2.0E-02	Ketimine reductase mu-crystallin
Q8K248	Hpdl	0.66	5.2E-02	2.1E-02	4-hydroxyphenylpyruvate dioxygenase-like protein
Q80WR5	NaN	-0.86	5.8E-02	2.3E-02	UPF0688 protein C1orf174 homolog
Q6Q2Z6	Acot5	0.62	5.9E-02	2.3E-02	Acyl-coenzyme A thioesterase 5
P07091	S100a4	0.69	6.3E-02	2.4E-02	Protein S100-A4
Q2TL60	Znf667	0.93	7.7E-02	2.9E-02	Zinc finger protein 667
Q9R226	Khdrbs3	0.66	8.1E-02	3.0E-02	KH domain-containing, RNA-binding, signal transduction-associated protein 3

Protein Accessions	Gene_name	log2(FC)	p-value	q-value	Protein Description
Q8VDR5	Tmem267	1.14	8.6E-02	3.2E-02	Transmembrane protein 267
P07607	Tyms	-1.23	8.9E-02	3.3E-02	Thymidylate synthase
Q5DTY9	Kctd16	0.55	9.7E-02	3.5E-02	BTB/POZ domain-containing protein KCTD16
Q9D1M7	Fkbp11	0.60	1.1E-01	3.8E-02	Peptidyl-prolyl cis-trans isomerase FKBP11
O88398	Avil	-0.64	1.1E-01	3.9E-02	Advillin
Q07243	Mtf1	-0.72	1.1E-01	3.9E-02	Metal regulatory transcription factor 1
Q3THF9	Coq10b	0.66	1.2E-01	4.2E-02	Coenzyme Q-binding protein COQ10 homolog B, mitochondrial
Q99MB1	Tlr3	0.61	1.2E-01	4.3E-02	Toll-like receptor 3
P04104	Krt1	-0.96	1.2E-01	4.4E-02	Keratin, type II cytoskeletal 1
O88992	C1ql1	0.84	1.4E-01	4.9E-02	C1q-related factor
Q61762	Kcna5	-1.02	1.5E-01	5.2E-02	Potassium voltage-gated channel subfamily A member 5
P07146	Prss2	0.54	1.5E-01	5.3E-02	Anionic trypsin-2
P02535	Krt10	-0.77	1.6E-01	5.5E-02	Keratin, type I cytoskeletal 10
Q9ESF1	Otof	0.76	1.9E-01	6.4E-02	Otoferlin
Q8CGN4	Bcor	0.66	2.2E-01	7.1E-02	BCL-6 corepressor
P03953	Cfd	0.64	2.3E-01	7.3E-02	Complement factor D
Q80W88	Homez	0.66	2.4E-01	7.7E-02	Homeobox and leucine zipper protein Homez
;B5X0G2;P02762;P04938;	18;Mup17;Mup6;Mup11;M	1.06	2.5E-01	7.9E-02	Major urinary protein 18; 17; 6; 11; 1
P15331	Prph	-0.88	2.6E-01	8.3E-02	Peripherin
P27573	Mpz	-0.81	2.8E-01	8.7E-02	Myelin protein P0
Q9CW42	Mtarc1	0.60	2.8E-01	8.7E-02	Mitochondrial amidoxime-reducing component 1
Q62190	Mst1r	0.70	3.5E-01	1.1E-01	Macrophage-stimulating protein receptor
Q6IFZ9	Krt74	-1.11	3.6E-01	1.1E-01	Keratin, type II cytoskeletal 74
Q9D2Y6	Fbxo25	0.61	4.2E-01	1.2E-01	F-box only protein 25
Q80YA8	Crb2	-1.30	4.2E-01	1.2E-01	Protein crumbs homolog 2
P70380	Il18	0.60	4.3E-01	1.3E-01	Interleukin-18
P70377	Fgf13	0.65	5.4E-01	1.5E-01	Fibroblast growth factor 13
Q8CFZ4	Gpc3	0.75	6.3E-01	1.7E-01	Glypican-3
