# Supplementary material for: Multi-omics characterization of radiation-induced cerebellar remodeling and tumorigenic transcriptional programs
Source: Neoplasia. 2026 Jun 29;79:101333. doi: 10.1016/j.neo.2026.101333 (PMC13330529; doi:10.1016/j.neo.2026.101333)
Supplement: Supplementary file 13 [file mmc13.pdf]

Supplementary Table 4: Transcriptome MB

MB Transcriptome 0.1Gy vs Sham

GeneID	Gene_name	log2(FC)	p-value	p-adj
ENSMUSG00000118012	Gm46620	-6.76	6.0E-32	1.4E-27
ENSMUSG00000105263	Gm42427	-4.64	7.1E-20	5.5E-16
ENSMUSG00000035929	H2-Q4	-1.94	1.2E-14	7.1E-11
ENSMUSG00000029814	Igf2bp3	3.79	8.2E-14	3.8E-10
ENSMUSG00000078922	Tgtp1	-3.29	4.8E-13	1.8E-09
ENSMUSG00000078853	Igtp	-2.26	2.5E-11	8.2E-08
ENSMUSG00000054072	Iigp1	-2.78	3.5E-11	1.0E-07
ENSMUSG00000019779	Frk	1.77	5.0E-10	1.1E-06
ENSMUSG00000126291	ENSMUSG00000126291	-8.29	7.9E-10	1.5E-06
ENSMUSG00000035493	Tgfbi	-1.52	2.8E-09	4.6E-06
ENSMUSG00000075010	AW112010	-1.52	7.3E-09	1.1E-05
ENSMUSG00000141894	ENSMUSG00000141894	-3.73	1.2E-08	1.5E-05
ENSMUSG00000060550	H2-Q7	-2.00	1.1E-08	1.5E-05
ENSMUSG00000030117	Gdf3	2.65	1.2E-08	1.5E-05
ENSMUSG00000037321	Tap1	-1.72	1.3E-08	1.5E-05
ENSMUSG00000029417	Cxcl9	-3.40	2.6E-08	2.8E-05
ENSMUSG00000031636	Pdlim3	1.85	3.5E-08	3.5E-05
ENSMUSG00000128598	ENSMUSG00000128598	-2.67	4.0E-08	3.9E-05
ENSMUSG00000041481	Serpina3g	-3.76	2.8E-07	2.4E-04
ENSMUSG00000090942	F830016B08Rik	-3.94	3.5E-07	2.9E-04
ENSMUSG00000038173	Enpp6	-1.72	3.8E-07	2.9E-04
ENSMUSG00000001506	Col1a1	-1.59	3.8E-07	2.9E-04
ENSMUSG00000036594	H2-Aa	-1.86	4.4E-07	3.1E-04
ENSMUSG00000079363	Gbp4	-1.95	5.4E-07	3.6E-04
ENSMUSG00000023078	Cxcl13	-3.10	7.5E-07	4.7E-04
ENSMUSG00000133301	ENSMUSG00000133301	-1.89	1.1E-06	6.6E-04
ENSMUSG00000058488	Kl	-1.65	1.2E-06	7.1E-04
ENSMUSG00000074151	Nlrc5	-1.93	1.7E-06	9.4E-04
ENSMUSG00000078302	Foxd1	-3.95	2.4E-06	1.3E-03
ENSMUSG00000021743	Fezf2	4.66	4.7E-06	2.4E-03
ENSMUSG00000069727	Zfp975	1.90	5.7E-06	2.7E-03
ENSMUSG00000121042	Gm33887	-4.27	6.4E-06	2.8E-03
ENSMUSG00000094930	Igkv6-25	-7.39	7.8E-06	3.3E-03
ENSMUSG00000024610	Cd74	-2.17	7.9E-06	3.3E-03
ENSMUSG00000014704	Hoxa2	-5.38	8.1E-06	3.3E-03
ENSMUSG00000096775	Gm5796	-5.29	8.8E-06	3.6E-03
ENSMUSG00000078921	Tgtp2	-2.08	9.7E-06	3.7E-03
ENSMUSG00000024112	Cacna1h	-1.58	9.7E-06	3.7E-03
ENSMUSG00000016756	Cmah	-2.29	1.3E-05	4.7E-03
ENSMUSG00000096727	Psmb9	-1.75	1.4E-05	4.9E-03
ENSMUSG00000073409	H2-Q6	-1.57	1.7E-05	5.6E-03
ENSMUSG00000068606	Gm4841	-4.10	2.0E-05	6.3E-03
ENSMUSG00000023341	Mx2	-1.83	2.1E-05	6.3E-03

GeneID	Gene_name	log2(FC)	p-value	p-adj
ENSMUSG00000119227	Gm25099	-2.15	2.2E-05	6.5E-03
ENSMUSG00000095105	Edaradd	-2.30	2.5E-05	7.3E-03
ENSMUSG00000073555	Iigp1c	-2.79	3.3E-05	9.3E-03
ENSMUSG00000073421	H2-Ab1	-1.70	3.3E-05	9.3E-03
ENSMUSG00000076672	Ighv3-6	-5.34	5.0E-05	1.3E-02
ENSMUSG00000097565	Gm26965	-5.27	5.7E-05	1.3E-02
ENSMUSG00000119286	Rnu5g	-2.52	5.3E-05	1.3E-02
ENSMUSG00000031506	Ptpn7	-2.28	5.2E-05	1.3E-02
ENSMUSG00000069874	Irgm2	-1.54	5.5E-05	1.3E-02
ENSMUSG00000140754	ENSMUSG00000140754	6.73	5.4E-05	1.3E-02
ENSMUSG00000127259	ENSMUSG00000127259	-2.64	6.0E-05	1.3E-02
ENSMUSG00000000386	Mx1	-2.25	6.2E-05	1.3E-02
ENSMUSG00000106334	Gm43549	-1.94	7.0E-05	1.5E-02
ENSMUSG00000096632	Igkv9-124	-5.60	8.7E-05	1.7E-02
ENSMUSG00000020890	Gucy2e	-2.54	8.8E-05	1.7E-02
ENSMUSG00000062743	Zfp677	1.63	9.2E-05	1.7E-02
ENSMUSG00000094694	Ighv1-9	-5.50	9.8E-05	1.8E-02
ENSMUSG00000110537	Gm4316	-2.49	1.1E-04	2.0E-02
ENSMUSG00000142387	ENSMUSG00000142387	-2.31	1.1E-04	2.1E-02
ENSMUSG00000093861	Igkv1-110	-3.27	1.2E-04	2.1E-02
ENSMUSG00000090231	Cfb	-1.79	1.4E-04	2.4E-02
ENSMUSG00000076937	Iglc2	-4.50	1.4E-04	2.4E-02
ENSMUSG00000139073	ENSMUSG00000139073	-3.97	1.4E-04	2.4E-02
ENSMUSG00000103692	4930503O07Rik	-4.04	1.5E-04	2.5E-02
ENSMUSG00000128875	ENSMUSG00000128875	-3.54	1.5E-04	2.5E-02
ENSMUSG00000016283	H2-M2	-3.08	1.6E-04	2.6E-02
ENSMUSG00000022758	P2rx6	-1.70	1.7E-04	2.7E-02
ENSMUSG00000081593	Gm11841	-2.23	1.7E-04	2.7E-02
ENSMUSG00000022768	Ccdc116	-2.08	1.7E-04	2.7E-02
ENSMUSG00000095130	Ighv1-39	-5.49	2.1E-04	3.1E-02
ENSMUSG00000105437	Gm42450	2.09	2.1E-04	3.1E-02
ENSMUSG00000095583	Ighv14-2	-5.17	2.3E-04	3.1E-02
ENSMUSG00000066113	Adamtsl1	-1.81	2.2E-04	3.1E-02
ENSMUSG00000104452	Ighv8-8	-4.72	2.4E-04	3.2E-02
ENSMUSG00000039942	Ptger4	-1.59	2.6E-04	3.4E-02
ENSMUSG00000095335	Igkv3-5	-5.14	2.6E-04	3.4E-02
ENSMUSG00000096459	Ighv9-3	-5.30	2.8E-04	3.5E-02
ENSMUSG00000041827	Oasl1	-2.74	2.9E-04	3.6E-02
ENSMUSG00000076615	Ighg3	-3.71	3.1E-04	3.9E-02
ENSMUSG00000034438	Gbp8	-1.74	3.2E-04	3.9E-02
ENSMUSG00000036098	Myrf	-2.22	3.3E-04	4.0E-02
ENSMUSG00000056445	Hoxaas2	-4.09	3.4E-04	4.1E-02
ENSMUSG00000090063	Dlx6os1	-3.54	3.5E-04	4.1E-02
ENSMUSG00000121571	ENSMUSG00000121571	-5.04	3.5E-04	4.2E-02
ENSMUSG00000076430	Hus1b	-1.69	3.9E-04	4.4E-02
ENSMUSG00000115529	9630013A20Rik	-1.94	3.9E-04	4.4E-02
ENSMUSG00000029754	Dlx6	-3.82	4.2E-04	4.6E-02

GeneID	Gene_name	log2(FC)	p-value	p-adj
ENSMUSG00000040264	Gbp2b	-1.96	4.4E-04	4.8E-02
ENSMUSG00000070407	Hs3st3b1	-2.77	4.6E-04	4.9E-02

MB Transcriptome 2Gy vs Sham

GeneID	Gene_name	log2(FC)	p-value	p-adj
ENSMUSG00000029814	Igf2bp3	4.05	5.2E-21	1.1E-16
ENSMUSG00000028023	Pitx2	-3.22	1.2E-13	1.3E-09
ENSMUSG00000057069	Ero1b	-1.58	2.1E-10	9.1E-07
ENSMUSG00000019888	Mgat4c	-2.02	5.9E-10	2.2E-06
ENSMUSG00000069170	Adgrv1	1.55	7.3E-10	2.3E-06
ENSMUSG00000021464	Ror2	-1.69	5.9E-09	1.3E-05
ENSMUSG00000031636	Pdlim3	1.99	3.9E-08	4.7E-05
ENSMUSG00000064672	Gm22806	-3.66	6.4E-08	6.7E-05
ENSMUSG00000051243	Islr2	2.20	1.7E-07	1.4E-04
ENSMUSG00000019779	Frk	1.53	2.3E-07	1.7E-04
ENSMUSG00000121171	Gm54112	4.10	2.9E-07	1.9E-04
ENSMUSG00000095105	Edaradd	-3.23	3.4E-07	2.0E-04
ENSMUSG00000080152	H3f4	4.05	3.2E-07	2.0E-04
ENSMUSG00000044303	Cdkn2a	-1.80	4.4E-07	2.4E-04
ENSMUSG00000038463	Olfml2b	-1.56	5.0E-07	2.6E-04
ENSMUSG00000030117	Gdf3	1.91	5.3E-07	2.7E-04
ENSMUSG00000029754	Dlx6	-6.80	8.7E-07	4.0E-04
ENSMUSG00000127716	ENSMUSG00000127716	-9.05	9.6E-07	4.4E-04
ENSMUSG00000124611	ENSMUSG00000124611	-5.79	1.6E-06	6.5E-04
ENSMUSG00000029371	Cxcl5	-1.84	2.8E-06	1.0E-03
ENSMUSG00000048015	Neurod4	-1.64	2.7E-06	1.0E-03
ENSMUSG00002076329	Gm54464	-5.82	3.0E-06	1.1E-03
ENSMUSG00000088208	Gm23751	-2.22	3.9E-06	1.3E-03
ENSMUSG00000064724	Gm25852	-2.49	4.3E-06	1.4E-03
ENSMUSG00000065371	Gm22739	-3.19	5.4E-06	1.7E-03
ENSMUSG00000035298	Klhl35	-2.19	6.8E-06	2.1E-03
ENSMUSG00000119670	Gm24305	-2.26	7.9E-06	2.4E-03
ENSMUSG00000123548	ENSMUSG00000123548	-1.54	7.9E-06	2.4E-03
ENSMUSG00000019817	Plagl1	1.51	9.4E-06	2.7E-03
ENSMUSG00000089837	Npcd	1.62	1.3E-05	3.4E-03
ENSMUSG00000038132	Rbm24	-1.76	1.4E-05	3.5E-03
ENSMUSG00000097052	Snora43	-2.55	1.4E-05	3.5E-03
ENSMUSG00000078302	Foxd1	-3.77	1.6E-05	4.0E-03
ENSMUSG00000021743	Fezf2	4.47	1.7E-05	4.2E-03
ENSMUSG00000065822	Snord15a	-2.37	1.8E-05	4.3E-03
ENSMUSG00000007908	Hmgcll1	1.51	1.9E-05	4.4E-03
ENSMUSG00000093413	Snora15	-2.31	3.3E-05	6.8E-03
ENSMUSG00000065725	Gm26165	-2.09	3.4E-05	6.9E-03
ENSMUSG00000064899	Snord118	-2.09	3.5E-05	7.1E-03
ENSMUSG00000119227	Gm25099	-3.00	3.7E-05	7.3E-03

GeneID	Gene_name	log2(FC)	p-value	p-adj
ENSMUSG00000064403	Gm23928	-3.50	3.9E-05	7.6E-03
ENSMUSG00000014704	Hoxa2	-5.78	3.9E-05	7.6E-03
ENSMUSG00000089542	Gm25835	-2.35	4.0E-05	7.7E-03
ENSMUSG00000044254	Pcsk9	2.15	4.2E-05	7.7E-03
ENSMUSG00000090063	Dlx6os1	-5.60	4.3E-05	7.9E-03
ENSMUSG00000142387	ENSMUSG00000142387	-2.62	4.4E-05	8.1E-03
ENSMUSG00000088948	Gm23262	-2.23	4.6E-05	8.3E-03
ENSMUSG00000119106	Gm23330	-3.23	4.6E-05	8.3E-03
ENSMUSG00000087963	Gm25394	-2.66	4.8E-05	8.5E-03
ENSMUSG00000012428	Steap4	-1.85	5.6E-05	9.6E-03
ENSMUSG00000118718	Gm24950	-1.97	6.0E-05	1.0E-02
ENSMUSG00000080465	Snord94	-2.42	6.1E-05	1.0E-02
ENSMUSG00000119776	Gm23849	-1.97	6.3E-05	1.1E-02
ENSMUSG00000140944	ENSMUSG00000140944	-2.09	6.6E-05	1.1E-02
ENSMUSG00000088025	Rprl3	-2.07	8.8E-05	1.4E-02
ENSMUSG00000065097	Snora16a	-2.38	9.1E-05	1.4E-02
ENSMUSG00000118057	B020010K11Rik	-2.17	9.3E-05	1.4E-02
ENSMUSG00000136853	Gm6653	-6.58	9.5E-05	1.4E-02
ENSMUSG00000112324	Gm47939	-3.28	9.5E-05	1.4E-02
ENSMUSG00000065208	Gm24616	-2.32	9.6E-05	1.4E-02
ENSMUSG00000037379	Spon2	-1.88	9.7E-05	1.4E-02
ENSMUSG00000119520	Gm24265	-1.81	1.0E-04	1.5E-02
ENSMUSG00000077192	Snora17	-2.06	1.1E-04	1.5E-02
ENSMUSG00000085129	5031425F14Rik	3.93	1.3E-04	1.8E-02
ENSMUSG00000092730	Snora24	-2.30	1.3E-04	1.8E-02
ENSMUSG00000037686	Aspg	-1.80	1.3E-04	1.8E-02
ENSMUSG00000103692	4930503O07Rik	-4.28	1.4E-04	1.9E-02
ENSMUSG00000003051	Elf3	5.09	1.4E-04	1.9E-02
ENSMUSG00000118837	Rnu2-10	-1.89	1.5E-04	1.9E-02
ENSMUSG00000065087	Snord22	-2.23	1.5E-04	1.9E-02
ENSMUSG00000064853	Gm23442	-1.89	1.5E-04	2.0E-02
ENSMUSG00000080365	Gm25776	-2.72	1.7E-04	2.2E-02
ENSMUSG00000064600	Gm25636	-2.00	1.9E-04	2.3E-02
ENSMUSG00000036446	Lum	-1.91	1.8E-04	2.3E-02
ENSMUSG00000069727	Zfp975	1.59	2.0E-04	2.4E-02
ENSMUSG00000107682	Gm33024	-4.72	2.2E-04	2.5E-02
ENSMUSG00000140640	ENSMUSG00000140640	-3.77	2.1E-04	2.5E-02
ENSMUSG00000118871	Gm23511	-3.03	2.2E-04	2.5E-02
ENSMUSG00000113811	Gm47882	-2.76	2.2E-04	2.5E-02
ENSMUSG00000065728	Gm26175	-2.60	2.2E-04	2.5E-02
ENSMUSG00000118859	Gm26316	-2.21	2.2E-04	2.5E-02
ENSMUSG00000064966	Snord15b	-2.18	2.0E-04	2.5E-02
ENSMUSG00000087935	Snora81	-2.13	2.2E-04	2.5E-02
ENSMUSG00000119142	Gm23143	-2.11	2.2E-04	2.5E-02
ENSMUSG00000087881	Gm22442	-2.09	2.2E-04	2.5E-02
ENSMUSG00000119320	Snora70	-2.06	2.1E-04	2.5E-02
ENSMUSG00000043613	Mmp3	-2.04	2.2E-04	2.5E-02

GeneID	Gene_name	log2(FC)	p-value	p-adj
ENSMUSG00000119132	Gm24407	-1.91	2.1E-04	2.5E-02
ENSMUSG00000064451	Snora23	-1.53	2.2E-04	2.5E-02
ENSMUSG00000090996	Gm20458	6.37	2.2E-04	2.5E-02
ENSMUSG00000064655	Gm25788	-2.00	2.3E-04	2.6E-02
ENSMUSG00000111829	Gm47475	-1.75	2.4E-04	2.6E-02
ENSMUSG00000077709	Snora64	-2.05	2.4E-04	2.6E-02
ENSMUSG00000064880	Gm24201	-2.40	2.5E-04	2.7E-02
ENSMUSG00000017737	Mmp9	-1.66	2.5E-04	2.7E-02
ENSMUSG00000064634	Gm22620	-1.99	2.5E-04	2.7E-02
ENSMUSG00000119873	Gm24497	-2.01	2.6E-04	2.7E-02
ENSMUSG00000035403	Crb2	2.97	2.7E-04	2.8E-02
ENSMUSG00000057729	Prtn3	-2.15	2.9E-04	2.9E-02
ENSMUSG00000103183	Gm37090	1.53	2.9E-04	2.9E-02
ENSMUSG00000056445	Hoxaas2	-4.86	2.9E-04	2.9E-02
ENSMUSG00000131748	ENSMUSG00000131748	-2.74	3.0E-04	2.9E-02
ENSMUSG00000006764	Tph2	3.29	3.0E-04	2.9E-02
ENSMUSG00000064901	Snora21	-1.98	3.1E-04	3.0E-02
ENSMUSG00000054083	Capn12	3.52	3.1E-04	3.0E-02
ENSMUSG00000068614	Actc1	-3.18	3.1E-04	3.0E-02
ENSMUSG00000064890	Gm22505	-4.01	3.1E-04	3.0E-02
ENSMUSG00000088990	Gm22767	-2.04	3.3E-04	3.1E-02
ENSMUSG00000064513	Snora9	-1.91	3.3E-04	3.1E-02
ENSMUSG00000088254	Gm24289	-1.98	3.7E-04	3.4E-02
ENSMUSG00000080364	Gm25777	-2.23	3.7E-04	3.4E-02
ENSMUSG00000065287	Gm24451	-1.77	3.8E-04	3.5E-02
ENSMUSG00000065016	Snora3	-1.82	4.1E-04	3.6E-02
ENSMUSG00000065232	Gm22973	-2.24	4.2E-04	3.7E-02
ENSMUSG00000119892	Gm23971	-1.77	4.2E-04	3.7E-02
ENSMUSG00000100454	Gm28901	-2.60	4.3E-04	3.8E-02
ENSMUSG00000103354	Gm38083	1.57	4.4E-04	3.8E-02
ENSMUSG00000077167	Gm24119	-1.61	4.5E-04	3.8E-02
ENSMUSG00000119388	Snord16a	-2.24	4.5E-04	3.9E-02
ENSMUSG00000093355	Snora26	-2.00	4.6E-04	4.0E-02
ENSMUSG00000064605	Gm22220	-2.57	4.7E-04	4.0E-02
ENSMUSG00000064994	Gm22422	-2.13	4.8E-04	4.0E-02
ENSMUSG00000065817	Gm24698	-2.14	5.0E-04	4.1E-02
ENSMUSG00000077563	Snora68	-1.70	5.1E-04	4.2E-02
ENSMUSG00000068117	Mei1	4.07	5.2E-04	4.2E-02
ENSMUSG00000044566	Cage1	-1.64	5.2E-04	4.3E-02
ENSMUSG00000118839	Gm25360	-1.87	5.3E-04	4.3E-02
ENSMUSG00000033060	Lmo7	-1.51	5.4E-04	4.4E-02
ENSMUSG00000064338	mt-Tv	-1.98	5.5E-04	4.5E-02
ENSMUSG00000106147	Snord3a	-1.74	5.6E-04	4.5E-02
ENSMUSG00000064382	Gm26447	-2.02	5.6E-04	4.5E-02
ENSMUSG00000064604	Snora44	-2.07	5.7E-04	4.5E-02
ENSMUSG00000014852	Adamts13	-4.04	5.7E-04	4.6E-02
ENSMUSG00000064666	Snora52	-1.90	5.8E-04	4.6E-02

GeneID	Gene_name	log2(FC)	p-value	p-adj
ENSMUSG00000065637	Gm26397	-2.06	6.0E-04	4.7E-02
ENSMUSG00000092819	Gm23639	-1.69	6.1E-04	4.7E-02
ENSMUSG00000015854	Cd5l	-2.11	6.4E-04	4.9E-02
ENSMUSG00000065118	Gm23297	-1.90	6.5E-04	4.9E-02
